# The effects of aftercare/resettlement services on crime and violence in children and youth: A systematic review

**DOI:** 10.1002/cl2.1404

**Published:** 2024-05-25

**Authors:** Jennifer S. Wong, Chelsey Lee, Natalie Beck

**Affiliations:** ^1^ School of Criminology Burnaby British Columbia Canada

**Keywords:** aftercare, meta‐analysis, qualitative, recidivism, re‐entry, resettlement, youth

## Abstract

**Background:**

High rates of youth re‐offending indicate that young custody‐leavers face challenges when reintegrating into their communities. Aftercare and resettlement programs can occur pre‐, during, and post‐release and generally provide multiple forms of support services to address youths' transitional needs.

**Objectives:**

The present review examines (1) the impact of youth aftercare/resettlement programs on crime‐related outcomes, (2) how treatment effect is moderated by participant, program, and study characteristics, (3) whether some types of interventions are more effective than others, (4) barriers/facilitators to effective program implementation, (5) the theory of change underlying resettlement interventions, and (6) available research on intervention cost.

**Search Methods:**

A comprehensive set of keywords and synonyms was combined in a Boolean search across 26 electronic databases. Multiple gray literature sources were also searched, including 23 journals, 4 meeting archives, 11 organization websites, 3 open access journal websites, and the CVs of 8 well‐known researchers in the field. The search was completed in January 2023.

**Selection Criteria:**

For objectives 1–3, studies were included if they utilized a randomized controlled design or quasi‐experimental comparison group design in which participants were matched on at least some baseline variables and included at least one quantitative individual‐measure of crime. For objective 4, included studies presented process evaluations of aftercare/reentry programs, clearly stated their research goals, and used qualitative methods in an appropriate way to answer the stated research question. For objectives 5 and 6, no specific methods were required; any study meeting the criteria for objectives 1–4 which presented findings on theory of change or cost data were included. For all outcomes, only studies conducted in a westernized country, and published after 1991 in English, French, or German were considered.

**Data Collection and Analysis:**

Two coders conducted primary data extraction for the included studies. Data were entered into a Microsoft Excel database. After data extraction, the two coders validated the coding by cross‐checking the database with each research report. Discrepancies between coders were discussed until consensus was reached. Where consensus could not be reached, a third coder was consulted. Study risk of bias was addressed using the ROBINS‐I (Sterne et al., 2016), ROB‐2 (Higgins et al., 2019), and the critical appraisal skills programme (CASP, 2018). Objectives 1–3 were addressed by synthesizing quantitative outcomes from rigorous impact evaluations of aftercare interventions using random effects models and meta‐regression. Thematic and narrative analysis was conducted to address objectives 4–6.

**Results:**

The search resulted in 15 impact studies, representing 4,718 participants across 21 program sites, and 35 effect sizes. The 21 impact evaluations were rated as having either low/moderate bias (*k* = 11) or serious bias (*k* = 10). The synthesis of 15 impact studies found no significant effects for arrest (*k* = 14; OR = 1.044, 95% prediction interval [0.527, 2.075], *t* = 0.335) or incarceration (*k* = 8, OR = 0.806, 95% prediction interval [2.203, 1.433], *t* = −1.674). A significant pooled effect was found for conviction (*k* = 13, OR = 1.209, 95% prediction interval [1.000, 1.462], *t* = 2.256), but results were highly sensitive to the inclusion of specific studies. No meaningful pattern of results emerged in moderator analyses with respect to study, sample, program component, or program delivery characteristics. The 19 process studies were rated as either high quality (*k* = 12) or moderate quality (*k* = 7). Thematic synthesis of the process evaluations revealed 15 themes related to the strengths/challenges of program implementation. The assessment of program cost (*k* = 7) determined a lack of data within the literature, preventing any summative analysis.

**Authors' Conclusions:**

Current evidence is promising with respect to conviction outcomes but overall does not find that aftercare/resettlement interventions have a reliably positive impact on crime‐related outcomes for young people who have offended. High variability across outcomes and reported data resulted in small sample sizes per outcome and limited moderator analyses. Multiple challenges for program implementation exist; additional rigorous research is sorely needed to further investigate the nuances of the program effects.

## 1 PLAIN LANGUAGE SUMMARY

### 1.1 Youth aftercare and resettlement programs to support transition out of custody have inconsistent effects on re‐offending, arrests and conviction

#### 1.1.1 The review in brief

This systematic review finds that youth aftercare/resettlement interventions do not produce consistent benefits or harms with respect to reoffending, including arrests, convictions, or incarceration. Conviction outcome results are promising, but the effect is small and unstable. Importantly, the number of studies in each of the three outcome groups was small, and the overall quality of evidence was hampered by a predominance of moderately strong research designs, small sample sizes, and a lack of peer‐reviewed studies. No clear pattern was found regarding the relationship between program and participant characteristics and treatment outcomes. Qualitative data suggest program facilitators face multiple challenges when implementing these programs, such as gaps in service immediately following release and staff turnover. There is a dearth of literature regarding the cost effectiveness of aftercare/resettlement programs.

#### 1.1.2 What is this review about?

Rates of reoffending among young custody‐leavers are high, with estimates ranging from 41% to 83% of youth committing a new offense post‐release. Youth experience many challenges during community reintegration, including barriers related to their education, employment, mental health, and social needs. To assist youth while they transition out of custody, aftercare/resettlement programs can occur pre‐, during, and post‐release and generally provide multiple services to address the young person's individual needs and reduce their risk for reoffending.

This review assessed whether aftercare/resettlement programs reduce future arrests, convictions, and/or incarceration among youth, and whether any program, participant, or study characteristics are linked to stronger treatment effects. Data regarding the strengths and challenges of program implementation, and the available literature on the costs of these programs, were also examined.

*Box: What is the aim of this review?*

This Campbell systematic review examines the effects of youth aftercare/resettlement programs on re‐offending, measured separately as arrests, convictions, and incarceration. The review reports on evidence from 15 intervention impact studies providing 35 independent effect sizes, and 19 qualitative implementation studies.

#### 1.1.3 What studies are included?

*Intervention impact studies*: Fifteen studies reporting on 21 program sites contributed 35 effects sizes examining arrest (*k* = 14), conviction (*k* = 13), and incarceration (*k* = 8). Studies were published between 1993 and 2021, and were primarily conducted in the United States, with three conducted in Western Europe. Four of the 15 studies were randomized controlled trials.

*Implementation studies*: Nineteen studies assessing the implementation of 18 programs met inclusion criteria for the review. Studies were published between 1998 and 2021, with six programs implemented outside of the United States.

*Cost assessment studies*: Seven studies providing cost data were identified. Four programs were implemented in North America, and three in the United Kingdom.

#### 1.1.4 What is the impact of aftercare/resettlement interventions on youth with respect to outcomes of crime?

No strong or consistent evidence suggests that youth aftercare/resettlement interventions are more effective at reducing incidents of arrests, convictions, or incarceration than are regular reentry services.

#### 1.1.5 How is the treatment effect moderated by factors such as participant, treatment, and study characteristics? Are some types of interventions more effective than others?

No program characteristics were related to important differences in reoffending outcomes. When looked at as subgroups, interventions involving community‐based service providers and programs without probation officer involvement positively impacted youth conviction rates.

Several study characteristics were related to stronger outcomes; studies rated as having a high degree of design bias produced a negative impact on arrest and incarceration, and studies using non‐randomized designs, with larger sample sizes, and with primarily ethnic minority samples showed positive effects on conviction.

#### 1.1.6 What are the barriers and facilitators to effective implementation of aftercare/resettlement interventions?

Aftercare programming often operates without seamless throughcare, which undermines the processes of rehabilitation and identity shifts towards prosocial attitudes. Poor communication, coordination, and data sharing between stakeholders is common and results in gaps in services and poor program fidelity. At times, sentencing and correctional practices seem to work in opposition to reentry processes; for example, short custodial sentences may not allow for proper transitional supports to be put in place. Dedicated and caring staff, structured communication across agencies, and planned long‐term funding can mitigate some of these challenges. Efficient administrative practices should be adopted that minimize the duplication of paperwork and maximize information sharing across agencies.

#### 1.1.7 What does the available research suggest regarding the cost of aftercare/resettlement interventions?

Only seven studies were included; the data were insufficient for synthesis.

#### 1.1.8 How has this intervention worked?

The assessment of the theory of change literature revealed additional factors to consider when implementing aftercare/resettlement programs, including the development of trusting relationships between transition teams and young people, facilitating youth motivation to change through greater program engagement, and the importance of a gradual transition process from custody to the community.

#### 1.1.9 What do the findings of this review mean?

Aftercare/resettlement programs have minimal impacts on youth with respect to recidivism, which may be related to various challenges and barriers faced during program implementation. More rigorous research addressing various program contexts, components, and populations is needed, as well as a greater focus on cost assessment.

#### 1.1.10 How up to date is this review?

Authors searched for studies published up to January 2023.

## 2 BACKGROUND

### 2.1 The problem, condition, or issue

Rates of post‐release offending for young custody‐leavers^1^ are high across the global west. For example, the UK House of Commons notes that 69.3% of those under age 18 reoffend within 1 year of being released from custody (House of Commons Justice Select Committee, 2021). In Australia, longitudinal data show that 41% of youth placed under supervision between 2000 and 2020 received additional sentences before turning 18; for those youth whose first sentence was custodial (vs. community‐based), rates were 10% higher (Australian Institute of Health and Welfare, 2021). European studies have found similar rates: in Germany, the reconviction rates of youth aged 14–15 who offended were estimated to be 46% from 2007 to 2010 (Jehle, 2014). While national youth recidivism rates are not available for Canada or the United States, provincial and state rates indicate similar levels of recidivism. For example, approximately 83% of youth who are incarcerated in British Columbia reoffend in the 5 years following their first offense (British Columbia Ministry of Children and Family Development, 2021), while 76% of Californian youth are rearrested within 3 years of release (Grassel et al., 2019). Aftercare/resettlement/reentry approaches^2^ support individuals as they leave custody and re‐enter their communities (Caputo, 2004). Generally, aftercare/resettlement programs include supervision as well as any service that is deemed to assist in the successful transition and reintegration of persons from custody to the community (Petersilia, 2004). Examples include assisting young people with access to safe accommodation, facilitating education and job training opportunities, and linking youth with community‐based substance abuse and counseling services (e.g., Barton et al., 2008; Bergseth & McDonald, 2007; Sontheimer & Goodstein, 1993).

Despite earlier summative evidence suggesting positive effects of aftercare/resettlement services on youth reoffending (Bouchard & Wong, 2018; James et al., 2013), recidivism rates remain unacceptably high, and the positive effects of such programs are often short‐lived (Hazel & Bateman, 2021). Recent reports suggest there may be significant programmatic gaps in meeting the reintegration needs of young custody‐leavers (House of Commons Justice Select Committee, 2021). Such gaps may include, for example, a lack of supports for education, housing, and job training, a failure to focus on internal narrative shifts and pro‐social identities, practices that are not gender‐informed, and a lack of inter‐agency collaboration that support young people upon release (Bateman & Hazel, 2018). Establishing an updated, comprehensive assessment of the overall impact of aftercare/resettlement programs on recidivism is paramount to providing current estimates of the effectiveness of aftercare services.^3^ Additionally, it is critical to determine how service components, features of program implementation, and participant characteristics are linked with stronger and weaker outcomes.

### 2.2 The intervention

Young people face multiple barriers and challenges post‐custodial release as they return to the community, often making it difficult to reintegrate into their home environments. Although some youths are able to successfully navigate the transition into a stable, prosocial life (Abrams, 2006; McCuish et al., 2018; Todis et al., 2001), many releasees struggle with successful reintegration. Youth sentenced to incarceration are completely removed from their home lives, disrupting and potentially halting any support they may have had from families, friends, and communities that could benefit the process of reentry (Mears & Travis, 2004; Ruch & Yoder, 2018). This experience is further complicated by the dual transition that young people involved in crime face, as they may be simultaneously transitioning from adolescence to adulthood (Altschuler & Brash, 2004).

Young custody‐leavers are confronted with numerous obstacles throughout the reentry process, including barriers related to their physical, psychological, educational, employment, and social needs – many of which are known to increase the risk of recidivism (Barrett et al., 2010; Fields & Abrams, 2010; Kubek et al., 2020; Mears & Travis, 2004). Young people who have offended may experience higher rates of mental and behavioral health needs in comparison to populations not involved in crime (Cauffman, 2004; Fazel et al., 2008; Young et al., 2017). Qualitative research suggests that young people view accessing mental health care as a daunting process, with systemic delays and minimal resources impacting their motivation and ability to seek help and access services (Aalsma et al., 2014). Adolescents also face numerous challenges regarding education and employment opportunities, such as labeling and stigma, a persistent pro‐offending identity, discrimination, negative attitudes from those in administrative positions, and an overall lack of support (Kubek et al., 2020). Further, young people involved in crime have noted social and personal obstacles, such as stress, anxiety, and fatigue (Bateman & Hazel, 2015), difficulties avoiding the negative and anti‐social peers and activities prevalent in their lives prior to their incarceration, and challenges in cultivating pro‐social friends and connections (Abrams, 2006). In addition, young people may face challenges accessing health care, substance use and addiction support, stable and safe housing, reliable transportation, and resources for developing skills to establish pro‐social and supportive relationships with friends, family, and other influential adults (Aalsma et al., 2015; Abrams, 2006; Barnert et al., 2020; Bateman & Hazel, 2018; Ruch & Yoder, 2018; Sinclair et al., 2021; Wright et al., 2012).

Upon release, adolescents face a very different environment outside the rigid structure of closed custodial settings, which can prove disorienting and stress‐inducing (Bateman & Hazel, 2015). Aftercare and resettlement programs differ from traditional models of post‐custodial supervision in two primary ways: (1) youth experience supervision as well as needs‐based supports and services (traditional post‐custodial supervision may or may not include services), and (2) youth typically participate in services while in custody, during the initial transition to the community, and for a supervised period post‐release from custody (Petersilia, 2004; Weaver & Campbell, 2015). While aftercare/resettlement programs often seek to ensure continuity of care throughout the custodial, transition, and post‐release periods, programs vary with respect to the specific components and services offered. In addition to supervision and frequent contacts between case workers and their clients, a common component is intensive case management; this component may include risk and needs assessments to appropriately match the adolescent to relevant supports and ensure that services continue throughout the transition and post‐release supervision periods (Altschuler & Armstrong, 1998).

More recently, resettlement policy and literature from the United Kingdom (UK) and Europe has rejected the risk paradigm in favor of approaches that focus on personal agency, identifying individual strengths, and empowering young people to shift to prosocial identities (Hazel & Bateman, 2021; Hazel et al., 2017; O'Mahony, 2009). Conversely, North American literature has leaned further into the risk paradigm, instead pushing for further development of the risk‐needs‐responsivity approach, including the use of risk reassessments and service delivery skills in practice (Baglivio et al., 2017; Looman & Abracen, 2013; Wormith & Zidenberg, 2018). In Australia, where aftercare/resettlement services are often referred to as “throughcare,” there is a noted lack of evaluation and other research on programming for the transition from custody to community, precluding the identification of a dominant theoretical framework (Day et al., 2019; Griffiths et al., 2017; Majeed et al., 2023). However, Australian governmental strategies and plans to reduce reoffending have in general been underpinned by desistance theory, restorative justice principles, the risk‐needs‐responsivity model, and life‐course perspectives (Australian Capital Territory Government, n.d.; Department for Correctional Services, Government of South Australia, 2016; Larsen, 2014; Strategic Services, Corrections Victoria, 2004).

Aftercare and resettlement services for children and young people who have offended may include psychological support and treatment, such as cognitive behavioral therapy (e.g., James et al., 2016) or anger management (e.g., Barton et al., 2008), as well as substance use interventions such as Alcoholics Anonymous or Narcotics Anonymous group sessions (e.g., Bergseth & McDonald, 2007; Bouffard & Bergseth, 2008; Wright et al., 2012). Part of the facilitation process may include case workers accompanying the young person to their programming (e.g., Bergseth & McDonald, 2007). Other services may include connecting youth to resources regarding housing and accommodation, such as ensuring that they have a safe and stable environment to return to after release, or helping to coordinate foster care placements (e.g., Barton et al., 2008). Employment and education services are also common, including job placements, vocational training, school supports, or a requirement for school attendance (e.g., Barton et al., 2008; Bouffard & Bergseth, 2008; Wiebush, 1993). Providing purposeful or constructive activities that help young people look towards their futures and direct their time towards non‐criminal activities is a staple of the resettlement process (Hazel, 2022; Wright et al., 2012). Additionally, programs may aim to increase relational supports for young people who have offended, including mentorship (e.g., Bergseth & McDonald, 2007; Bouffard & Bergseth, 2008) and familial supports such as family conferencing (e.g., Wiebush, 1993) or family group therapy (e.g., Greenwood & Turner, 1993). Some youth may access a wide array of services and supports as part of their reentry strategy, while others participate in fewer services.

### 2.3 How the intervention might work

Aftercare and resettlement programs were originally developed in response to the need for continued support post‐custodial release and are based on the theoretical underpinning that more supervision and resources will lead to improved outcomes and reduced recidivism. Specifically, programs seek to increase pro‐social identities, opportunities, skillsets, and behaviors (Hazel et al., 2017; Hazel, 2022), while reducing antisocial influences, attitudes, and behaviors (Bouffard & Bergseth, 2008; Chung et al., 2007). Theories of cognitive transformation derived from social control axioms provide some insight into the identity change process that happens during desistance and stress the importance of individual differences (such as motivation levels and openness to change) and their effects on lasting prosocial behavior (Giordano et al., 2002). Many North American aftercare/resettlement programs incorporate aspects of strain theories, learning theories, and social control theories (Bergseth, 2010), and focus on a risks‐based approach which aims to target factors that make individuals more likely to reoffend (Burnett, 2010; Hazel & Bateman, 2021). However, European and UK aftercare/resettlement program logic has deviated from the risk paradigm (which is based in the facets of social learning theory) and adopted a strengths‐based, positive youth justice approach, wherein individual strengths are highlighted in the desistance process (Burnett, 2010; Case & Haines, 2018). Further, the promotion of “secondary desistance,” closely aligned with Giordano et al.'s (2002) theory of cognitive transformation, has been adopted in some reentry practices, wherein aftercare/resettlement staff encourage individuals to adopt new, non‐offender identities by facilitating rehabilitative and pro‐social activities (Burnett, 2010; Hazel et al., 2017; McNeill, 2006).

**Figure 1:**
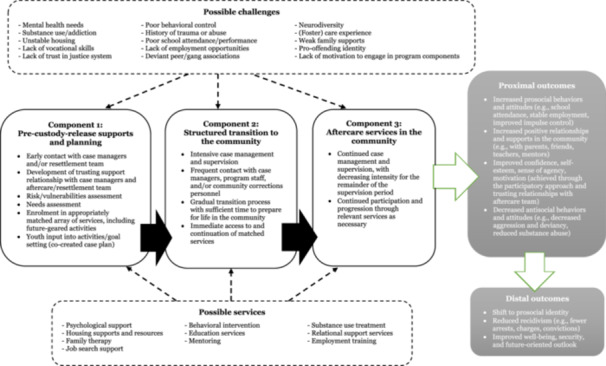
Youth aftercare/resettlement theory of change.

In 1988, the U.S. Office of Juvenile Justice and Delinquency Prevention (OJJDP) initiated efforts surrounding adolescent reentry concerns, resulting in the development of the Intensive Aftercare Program model (IAP) (Altschuler & Armstrong, 1994, 2001). The IAP model emphasizes three components: (1) intervention and rehabilitation programming completed during custody to prepare the individual to reintegrate into the community, (2) a structured transitional process that bridges the institution and community and develops connections between the two environments, and (3) intensive supervision and intervention services provided in the community (Altschuler & Armstrong, 1998). Similarly, UK models stress the importance of five characteristics during the resettlement process: (1) constructive intervention which encourages the development of a pro‐social identity, (2) co‐created plans which involve the young person, informal support systems, and formal support systems, (3) customized support that takes into account the custody‐leaver's background, strengths, and vulnerabilities, (4) consistent care that spans from (pre)sentencing to successful life in the community, and (5) a coordinated and multi‐agency approach (Bateman & Hazel, 2018; Hazel et al., 2017, 2022). These components are integral to comprehensive resettlement approaches; see Figure 1 for a description of the theory of change.

#### 2.3.1 Custodial phase

The pre‐release stage of aftercare/resettlement programs provides young people in custody the opportunity to build a strong foundation of skills and behaviors necessary for easing the transition post‐release. Research suggests that successful reintegration is more likely if release planning and intervention programming begin when the individual is admitted into custody, before the start of the transition (Bateman et al., 2013; Byrnes et al., 2002; Petersilia, 2004; Travis & Petersilia, 2001). In accordance with this principle, the case management component of the reentry model should ideally commence at entry into custody and continue throughout the supervision period. Important in the case management process is an element of co‐creation; more specifically, by allowing young people to have some self‐determination in their plans for the future, the development of a pro‐social identity may be strengthened (Bateman & Hazel, 2018). Eligible young people will generally undergo risk, vulnerabilities, and needs assessments and engage in discussions surrounding their personal goals and motivations, which will inform their case planning with respect to level of supervision and the specific interventions provided while in custody. These interventions typically focus on preparing custody‐leavers to live pro‐socially post‐release, and may include psychological or behavioral interventions (e.g., cognitive behavioral therapy, anger management) or substance use treatment, mental and physical health services, education services, discharge planning such as identifying a safe living situation, and/or employment preparation and training (e.g., Hampson & Tracey, 2016; James et al., 2013). Further, case planning engages informal networks of support *before* release, in order to provide young people with coordinated support throughout the entire reentry process (Bateman & Hazel, 2018).

#### 2.3.2 Transition phase

The immediate period of transition is a critical point in the overall reintegration process. Research suggests that re‐offense is most likely to occur shortly after release, indicating the need for intensive and highly structured transition planning, and immediate access to matched services (Altschuler & Armstrong, 1998; Goodstein & Sontheimer, 1997; Griffin, 2004). As such, aftercare/resettlement models typically involve intensive case management and supervision immediately after release, with frequent contact between case managers and community correctional staff (Altschuler & Armstrong, 1998; Greenwood & Turner, 1993; Griffin, 2004; James et al., 2016). This can include check‐in meetings, drug testing, family conferencing, and various mentorship activities (Bergseth & McDonald, 2007; Bouffard & Bergseth, 2008; Sontheimer & Goodstein, 1993; Wiebush, 1993). Agencies from all stages of the resettlement process are encouraged to attend meetings during this transitional phase, in order to provide coordinated and unified care which is unaffected by the spatial changes involved in release (Bateman & Hazel, 2018; Hampson & Tracey, 2016).

#### 2.3.3 Post‐release phase

After the initial transition to the community has occurred, young custody‐leavers are generally required to maintain contact with their case manager to ensure continued supervision and service participation, including reassessment of needs as necessary (Altschuler & Armstrong, 1998; Griffin, 2004; James et al., 2013). The intensity of this care gradually decreases over time as the young person becomes more established within the community (Griffin, 2004). To successfully implement this phase, it is critical that the aftercare/resettlement team has established strong connections with community‐based services and resources to ensure that youth receive the appropriate continuity of care to prevent any lapses or delays in service delivery and subsequent skill and behavioral regression (Altschuler & Armstrong, 2001; Mathur & Clark, 2014). The scope of the available services should be broad in an effort to meet the various needs of the adolescents and ensure that services provided in the community parallel those provided in custody (Altschuler & Armstrong, 1998; Byrnes et al., 2002).

### 2.4 Why it is important to do this review

Three prior reviews have been conducted on the topic of aftercare/resettlement interventions on recidivism for young people who have offended. First, James et al. (2013) meta‐analyzed 22 studies of experimental and quasi‐experimental reports, with recidivism defined as official reports of re‐arrests and/or reconvictions. The authors found a small, positive overall effect size, suggesting that in comparison to the control group (care as usual), youth who participated in an aftercare/resettlement program post‐release from a secured facility were significantly less likely to recidivate (*d* = 0.12, *p* < 0.001). Moderator analyses indicated certain characteristics of interventions were related to stronger impacts (specifically, individual treatment, intensive treatment, interventions targeted to youth involved in serious crime, and interventions targeted to older youth). In addition, well‐implemented programs (as opposed to those noting considerable implementation challenges) were related to stronger outcome effects.

Next, Weaver and Campbell (2015) meta‐analyzed 30 evaluations of youth aftercare/resettlement programs and found a non‐significant overall treatment impact on recidivism (RR = 0.931, *p* = 0.117). Risk ratios ranged from 0.391 to 2.095, indicating substantial variability in the effects of the selected interventions. Moderator analyses found that interventions were most effective for youth over the age of 16.5 years, and for youth with a predominantly violent index offense. Similar to James et al. (2013), Weaver and Campbell (2015) reported that programs without implementation difficulties were related to stronger, positive impacts, as were randomized research designs and publications in peer‐reviewed sources.

Notably, although the reviews by James et al. (2013) and Weaver and Campbell (2015) addressed the same research question, the reviews resulted in conflicting overall conclusions with respect to the impact of aftercare/resettlement programs on recidivism. This discrepancy is in large part due to differences in author operationalization of “aftercare” interventions, as well as differences in the review inclusion criteria. More specifically, Weaver and Campbell (2015) included studies in which the intervention served specifically as an aftercare approach to shock incarceration and boot camp programs, while James et al. (2013) restricted their set of studies to aftercare/resettlement programs which “incorporated a treatment modality such as skills training, counseling, and cognitive behavioral therapy” and excluded studies in which the intervention focused on discipline or surveillance (p. 265).

In 2018, Bouchard and Wong published a systematic review and meta‐analysis on the impacts of community‐based aftercare (as well as intensive supervision programs) on crime‐related outcomes for youth. The search was restricted to studies published after 1990, excluding older studies focused on shock incarceration and boot camp programs (which are no longer common approaches to aftercare/resettlement). Strict selection criteria were implemented in an effort to increase the generalizability of results to the “typical” offender; further, the authors separated results into “alleged” (e.g., arrests, police contacts) versus “convicted” (e.g., convictions, incarcerations) offenses. In total 10 studies were included, which represented 15 independent program sites. Bouchard and Wong found a positive and significant estimate of treatment impact on alleged offenses (LOR = 0.179, *z* = 2.18, *p* = 0.029), but no significant impact for convicted offenses (LOR = −0.029, *z* = 0.27, *p* = 0.784). For alleged offenses, moderator analyses found that weaker research designs, shorter follow‐up periods, and larger sample sizes were related to stronger treatment effects, while for convicted offenses shorter follow‐up periods were related to stronger impacts. The latter finding suggests that the impact of aftercare/resettlement programs may not have a lasting effect on reducing recidivism.

The current review updates these prior reviews on aftercare/resettlement programs with the inclusion of new research published in the 7.5 years since Bouchard and Wong conducted their literature search (i.e., in 2015). In addition, the inclusion criteria with respect to intervention type is expanded to include studies focused on substance abusers, as well as to interventions implemented in both community and closed‐custody settings. The current study also includes a qualitative evidence synthesis of process evaluation data from both experimental and non‐experimental studies, and a narrative summary of available cost‐effectiveness data. It is well known that young people often face difficulties after release from a custodial stay and are in need of assistance during the process. It is important that the services and programs designed to provide this assistance are continuously evaluated to safeguard the best care possible for youth. This knowledge is critical to ensure that program funding is directed appropriately and that resources are being used effectively. The review seeks to provide decisionmakers with information relevant to considerations of best practices for program components, target population, and approach, and provide evidence for practitioners and policymakers with regard to potential barriers and facilitators to program implementation.

## 3 OBJECTIVES

The goal of the current study is to examine the impacts of aftercare/resettlement interventions on youth with respect to crime‐related outcomes, and to examine factors related to intervention success. Specific objectives are as follows:

(1) What is the impact of aftercare/resettlement interventions on youth with respect to outcomes of crime and violence?
(2) How is the treatment effect of aftercare/resettlement interventions on crime and violence outcomes moderated by factors such as participant (e.g., age, race, ethnicity, sex, offender type), treatment (e.g., intensity and quality of implementation), methodological (e.g., measurement of crime, study design, timing of follow‐up measures), and study characteristics (e.g., date of publication, peer‐reviewed status)?
(3) Are some types of aftercare/resettlement interventions more effective than others?
(4) What are the barriers and facilitators to effective implementation of aftercare/resettlement interventions?
(5) What are the mechanisms (theory of change) underlying aftercare/resettlement interventions?
(6) What does the available research suggest regarding the cost of aftercare/resettlement interventions?

## 4 METHODS

This review follows the methodological expectations of Campbell Collaboration intervention reviews (The Methods Group of the Campbell Collaboration, 2019). A protocol for the review was published in January 2023 (Wong et al., 2023). The review is informed by theory‐based impact evaluation (White, 2018), as specified in the theory of change outlined in Figure 1. To address objectives 1, 2, and 3, we systematically collected and synthesized quantitative outcomes from rigorous impact evaluations of aftercare/resettlement interventions on young people who have offended.

To address objective 4, we collected and examined both quantitative and qualitive research describing the implementation of aftercare/resettlement interventions, including process evaluations which examine barriers and facilitators to success from the perspectives of both youth and staff. To achieve objective 5, we revised the theory of change described in the review protocol based on retrieved research. Finally, to increase the utility of this review for policymakers and other decisionmakers, we aimed to address objective 6 by narratively summarizing available evidence on intervention costs in relation to outcomes and participants served.

### 4.1 Criteria for considering studies for this review

#### 4.1.1 Types of studies

##### Study objectives 1–3

For objectives 1–3, included studies were restricted to rigorous or moderately rigorous research designs, including randomized controlled trials and quasi‐experimental comparison group designs in which participant baseline variables (e.g., criminal history, age, sex) were examined for observable between‐group differences. Eligible comparators were similar groups of youth who did not participate in an aftercare/resettlement program or receive reentry services. Single group pretest‐post‐test designs, as well as comparison group designs deemed weak in baseline matching (i.e., with groups found inequivalent on important factors such as risk level and criminal history, or baseline group comparability not provided), were excluded.

##### Study objective 4

For objective 4, included studies were restricted to implementation assessments of an aftercare/resettlement intervention, without restriction on study research design, or requirement for quantitative outcomes of violence and crime. Both quantitative and qualitative research were included. Qualitative studies (or mixed methods studies with a qualitative component) identified through the search were appraised using the Critical Appraisal Skills Program (CASP) qualitative research checklist (2018). Included studies met, at minimum, the requirements of questions 1–2 on the checklist; specifically, the study provided a clear statement of the aims of the research, and a qualitative methodology was appropriate to meet these aims. See Supporting Information: Appendix 1 for an overview of the checklist.

##### Study objective 5

Data for objective 5 were obtained if information on program intervention theory was presented in any of the studies included for objectives 1–4, or if studies relevant to an aftercare/resettlement theory of change were identified in the search of gray literature. Included studies were not restricted to those presenting a program evaluation as long as they presented a discussion or description of aftercare/resettlement intervention theory of change.

##### Study objective 6

For objective 6, data were obtained if analyses on program cost–benefit, cost‐effectiveness, or cost‐per‐participant served were presented in any of the studies included for objectives 1–5.

#### 4.1.2 Types of participants

Eligible program participants for this review include children and young people who have served or are currently serving a custodial sentence, with an age range of 10–18 years. Studies were also eligible for inclusion if the participant age range exceeded 18 years (up to 21 years), provided that the average age of study participants was at or below 18. There were no restrictions based on gender, ethnicity, or offense.

Excluded participants include offenders being offered a specialized program based on their exceptional/unique characteristics; for example, those with serious mental health diagnoses. These types of participants were excluded as findings from such studies would not be generalizable to broader groups of young people participating in aftercare/resettlement programs.

#### 4.1.3 Types of interventions

Eligible types of interventions include any aftercare/resettlement program that takes place while the young person is in custody, during their transition to the community, and/or when they have returned to the community. The overarching intervention objective should be to promote successful community reintegration and/or reduce reoffending. Program components may vary across interventions (e.g., intensive case management, housing support, education, counseling), and interventions were not required to contain any particular type of component so long as the intervention was framed as an aftercare/resettlement program as opposed to, for example, a more general skills training program or substance abuse recovery program.

Boot camp interventions, wilderness therapy interventions, and intensive supervision programs were excluded as they represent very specific models that are dissimilar from more generalized aftercare programs. Also excluded were probation programs that did not meet the definition of a resettlement approach (e.g., those that include only supervision without additional supports and resources). In order to increase the generalizability of findings, programs limited to very specific types of individuals were excluded, such as specialized programs targeting adolescents who have committed sex offenses.

#### 4.1.4 Types of outcome measures

##### Primary outcomes

###### Study objectives 1–3

For objectives 1–3, the primary outcomes included quantitative, individual‐level measures of crime. Outcomes were categorized into subgroups including new arrest/criminal contact, new conviction, and new incarceration.^4^ Status offenses (e.g., truancy, traffic violations) and technical probation violations were excluded.

###### Study objective 4

For objective 4, the primary outcomes included any qualitative or quantitative measures related to intervention implementation and process outcomes. Expected outcomes included but were not limited to:

- discussions of gaps in services (e.g., seamless throughcare services, whether necessary community‐based services were available),
- participant perceptions of barriers to program engagement,
- practices regarding interagency collaboration and communication (e.g., which data were shared between agencies and how),
- issues concerning staff or participant safety (e.g., caseworker visits to high‐risk areas),
- resource allocation and casework feasibility (e.g., participant to caseworker ratios; sufficiency of resources for providing a high standard of care),
- program implementation fidelity (e.g., how far the program deviated from its design),
- participant engagement with services and resources (e.g., expected vs. actual service hours or participation; underutilized services; services in high demand), and
- unanticipated challenges with respect to service delivery.

###### Study objective 5

For objective 5, relevant outcomes included any information on intervention theory of change, such as causal pathways between program inputs and expected outcomes.

###### Study objective 6

The primary outcomes for objective 6 included information concerning intervention cost in relation to outcomes achieved, such as a cost–benefit analysis or cost‐effectiveness analysis, or cost data such as annual program cost in relation to number of clients served.

##### Secondary outcomes

For objectives 1–3 and 5–6, no additional outcomes were considered. For objective 4, qualitative data that did not fit within the a priori outcomes categories were coded inductively, allowing other relevant outcomes to present themselves.

#### 4.1.5 Duration of follow‐up

No restrictions were placed on the duration of participant follow‐up; for objectives 1–3 quantitative outcomes were coded for all available time periods for all included studies. While our intent was to synthesize studies by length of follow‐up period, few included studies used time periods that were less than 12 months or greater than 18 months. As the large majority of studies used a 12‐month follow‐up period, this outcome time point was used across all studies when available for the main pooled analyses. Duration of follow‐up was explored in sensitivity analyses; for example, by conducting smaller analyses of two or three studies with a 6‐month time period, and dropping studies from the main analyses if outcomes were greater or longer than 12 months. The sensitivity analyses allowed for an examination of only those studies using the same period of follow‐up, thus reducing variation in the pooled sets with respect to timing of outcome measure.

#### 4.1.6 Types of settings

No restrictions were placed on intervention settings for studies addressing any of objectives 1–6. With respect to geographical setting, studies were restricted to those conducted in Canada, the United States, Australia, New Zealand, the United Kingdom, or a Western European country. This criterion was implemented in an effort to enhance the commensurability and practicality of the study findings, as social/political systems and approaches to criminal justice vary substantially across nations from different geopolitical regions.

#### 4.1.7 Other criteria

Studies were limited to research published after 1991 (i.e., 30 years; to focus on more contemporary approaches to aftercare/resettlement), and to research published in English, French, or German (given the evaluation team's existing expertise). As noted in the protocol, studies published in additional languages would have been retrieved and documented, and a decision concerning the necessity for translation services would have been made. However, no studies met this criterion. No restrictions were placed on publication status or type (e.g., peer‐reviewed source; published in a government report vs. journal article).

### 4.2 Search methods for identification of studies

#### 4.2.1 Electronic searches

The following set of key terms was used in a Boolean search, reflecting four primary constructs: (1) youth/young person, (2) offending, (3) aftercare/resettlement intervention, and 4) evaluation, theory, or cost:

*Construct 1*: (youth* OR juvenile* OR adolesc* OR teen* OR "young offender*" OR "young people" OR "young person*" OR child* OR "early offender") AND

*Construct 2*: (crime* OR criminal* OR devian* OR violen* OR delinquen* OR offend* OR offense* OR offence* OR recidiv* OR reoffen* OR breach* OR "technical violation*" OR arrest* OR convict* OR charge* OR incarcer* OR petition* OR adjudicat* OR caution* OR "compliance during supervision" OR "return to custody") AND

*Construct 3*: (diversion* OR divert* OR probat* OR parole OR aftercare OR resettlement OR reentry OR "re‐entry" OR "after custod*" OR supervis* OR "graduated sanction*" OR "intermediate sanction*" OR "early release" OR "pretrial release" OR "supervised release" OR wraparound OR reintegrat* OR throughcare OR "local authority care" OR "security training cent*" OR "care leaver*" OR "detention and training order" OR "youth offending team") AND

*Construct 4*: (evaluat* OR effect* OR impact* OR outcome* OR trial* OR treat* OR program* OR randomi* OR experiment* OR assess* OR process OR implement* OR fidelity OR "proof of concept" OR "case study" OR "focus group" OR "pilot study" OR qualitative OR "formative evaluation" OR "cost benefit" OR "cost effectiveness" OR "cost analysis” OR "benefit cost" OR "theory of change" OR "program* theory" OR "program model" or "logic model" OR "action theory" OR "causal map")

The search was conducted between December 29, 2022, and January 13, 2023 (see Supporting Information: Appendix 3 for complete search strategies and dates), and encompassed documents published between January 1992 and January 2023. The complete search strategy was applied to a series of 26 electronic databases, including various social sciences databases and those more specifically focused on criminology and criminal justice. Terms were searched in the “abstract,” “title,” and “subject” search fields where available. The complete search strategies are detailed in Supporting Information: Appendix 3. The following databases were searched; those on the same search platform were combined:

- *EBSCO*:
  ○ Academic Search Premier
  ○ Criminal Justice Abstracts
  ○ EBSCO Open Dissertations
  ○ Education Source
  ○ ERIC
  ○ Medline
  ○ PsycARTICLES
  ○ PsycBOOKS
  ○ PsycINFO
  ○ Social Sciences Abstracts
  ○ Social Sciences Full Text
- *Elsevier*: Scopus
- Networked Digital Library of Theses and Dissertations (NDLTD)
- *OVID*:
  ○ Cochrane Central Register of Controlled Trials
  ○ Cochrane Database of Systematic Reviews
  ○ Database of Abstracts of Reviews of Effects
- *ProQuest*:
  ○ Applied Social Sciences Index and Abstracts (ASSIA)
  ○ Canadian Research Index
  ○ National Criminal Justice Reference Service (NCJRS)
  ○ ProQuest Dissertations and Theses
  ○ Social Services Abstracts
  ○ Sociological Abstracts
  ○ Sociology Database
- Open Access Theses and Dissertations
- Theses Canada
- Web of Science:
  ○ Arts & Humanities Citation Index (AHCI)
  ○ Conference Proceedings Citation Index – Science (CPCI‐S)
  ○ Conference Proceedings Citation Index – Social Sciences & Humanities (CPCI‐SSH)
  ○ Emerging Sources Citation Index (ESCI)
  ○ Science Citation Index Expanded (SCI‐EXPANDED)
  ○ Social Sciences Citation Index (SSCI)

Additional web sources were searched to identify relevant unpublished works not indexed in academic databases (e.g., technical reports, conference papers, and independent research projects) in an effort to minimize the risk of publication bias. Specifically, we searched for relevant conference and meeting abstracts via the meeting archives of the American Society of Criminology, the British Society of Criminology, the European Society of Criminology, and the Conference Proceedings Citation Index. In addition, the websites of several relevant organizations were searched, including the Australian Institute of Criminology, Confederation of European Probation, Department of Justice Canada, European Crime Prevention Network, Home Office UK, Ministry of Justice UK, National Institute of Justice, New South Wales Bureau of Crime Statistics and Research, Office of Juvenile Justice and Delinquency Prevention, U.S. Department of Justice, and the Youth Justice Board. Last, three open access search engines were searched: the Directory of Open Access Journals (DOAJ), Bielefeld Academic Search Engine (BASE), and CrimRxiv. See Supporting Information: Appendix 3.

Search terms on websites, meeting archives, and open access engines included a reduced set of the terms from the database search strategy and were adjusted across different sites and search restrictions accordingly. If the site allowed for advanced search strategies, we used multiple combined terms; if the site allowed for only basic search strategies, we attempted a series of searches such as (“young person” AND resettlement) or (youth AND aftercare).

#### 4.2.2 Searching other resources

Additional search methods included hand‐searching the reference lists of existing review literature in the field (e.g., Weaver & Campbell, 2015), and the reference lists of all studies meeting inclusion criteria. We also reviewed the curricula vitae of well‐known researchers in the field (Tim Bateman; Jeffrey Bouffard; Anne‐Marie Day; Barry Goldson; Kate Gooch; Pippa Goodfellow; Neal Hazel; John Pitts). Further, websites specifically affiliated with an aftercare/resettlement program were searched for evaluations (e.g., Boys and Girls Clubs of America, Abraxas Non‐Residential Care). The search also included the tables of contents of the following 23 journals, backdated to 24 months prior to the date of implementation of the electronic database search:

- *British Journal of Criminology*
- *Canadian Journal of Criminology*
- *Canadian Journal of Criminology and Corrections*
- *Canadian Journal of Criminology and Criminal Justice*
- *Corrections: Policy, Practice & Research*
- *Crime and Delinquency*
- *Crime Prevention and Community Safety*
- *Criminal Justice and Behavior*
- *Criminal Justice Review*
- *Criminology & Public Policy*
- *Criminology and Criminal Justice*
- *European Journal of Crime, Criminal Law, and Criminal Justice*
- *European Journal of Criminology*
- *European Journal on Criminal Policy and Research*
- *Federal Probation*
- *International Journal of Offender Therapy and Comparative Criminology*
- *Journal of Community Corrections*
- *Journal of Experimental Criminology*
- *Journal of Offender Rehabilitation*
- *Journal of Research Crime and Delinquency*
- *Juvenile and Family Court Journal*
- *Probation Journal*
- *Residential Treatment for Children & Youth*
- *Youth Justice*
- *Youth Violence and Juvenile Justice*

### 4.3 Data collection and analysis

#### 4.3.1 Selection of studies

The initial list of sources for the database and gray literature search were split into two sections (approximately half for each reviewer) with Reviewer 1 (CL) conducting the search for the first section, and Reviewer 2 (NB) conducting the search for the second section. Specifically, each reviewer read through the titles and abstracts of identified hits in their designated set of databases/sources to determine studies that appeared potentially relevant to the review objectives. Studies were deemed potentially relevant if they appeared to focus on youth aftercare/resettlement programs and included any quantitative, qualitative, or cost data. At this point in the selection process, obviously irrelevant hits were excluded (e.g., book reviews, non‐human studies), including those that clearly met any of the exclusion criteria (e.g., used an adult population; were published in the 1980s). The lists from both reviewers were then combined into a single database and reviewed a second time by both reviewers to determine which studies to retrieve in full. Once the full text documents had been retrieved, two reviewers (CL and NB) independently applied the inclusion and exclusion criteria to every article in the list to determine whether it met the full set of selection criteria for objectives 1–3 and/or objectives 4, 5, or 6. Discrepancies between reviewers were resolved by a third reviewer (JSW).

#### 4.3.2 Data extraction and management

The included studies were split in half, with two coders conducting primary data extraction for each study in their set. Data were entered into a Microsoft Excel database. After primary data extraction, two coders (CL and NB) validated each other's coding by cross‐checking the database coding with each research report. Discrepancies between coders were discussed until consensus was reached. Where consensus could not be reached, a third coder (JSW) was consulted.

##### Study objectives 1–3

For objectives 1–3, an extensive coding scheme involving approximately 75 variables was used to extract data from each study. The coded variables fell into categories of general study characteristics (e.g., publication type, program delivery year), program components (e.g., vocational skills, family involvement), information about the study sample (e.g., age, racial mix, gender mix, sample size), research design characteristics (e.g., type of research design, baseline group differences, outcome measure, follow‐up period), and outcomes (e.g., means and standard deviations, frequencies, group sample sizes, *t*‐statistics).

##### Study objective 4

For objective 4, implementation studies were coded using 37 variables. Extracted information related to study details (e.g., program name, program description), study quality (e.g., reporting practices, ethical considerations, limitations), data characteristics (e.g., types of data presented, methods of data analysis, types of participants), and process evaluation outcomes (e.g., program fidelity, service provision, casework feasibility, communication, staffing). After initial coding on these 37 a priori variables was complete, further data extraction took place by identifying emergent variables based on trends across the included studies. This process is described in more detail under Data Synthesis.

##### Study objective 5

For objective 5, any information concerning the intervention's theory of change was extracted, for example, theorized pathways for participants as they move between program activities towards desired outcomes.

##### Study objective 6

For objective 6, studies were coded on a series of variables related to any intervention cost analyses (e.g., cost–benefit, cost‐effectiveness, cost in relation to participants served). Identified variables include any program or staffing costs, cost per participant, annual costs or budget, or costs related to the justice system (e.g., incarceration, court processes, police/arrests, etc.), and findings of any cost analysis or comparison made. Notably, the studies varied considerably in terms of the type and extent of the cost analysis or comparison presented, and the resulting coding and data extracted were limited.

See the draft coding form in Supporting Information: Appendix 2 for more details.

#### 4.3.3 Assessment of risk of bias

##### Study objectives 1–3

Studies were assessed by two independent reviewers for potential risk of bias using either the Cochrane Risk of Bias in Non‐randomized Studies of Interventions (ROBINS‐I; Sterne et al., 2016) or the Cochrane Risk of Bias tool for randomized studies (RoB‐2; Higgins et al., 2019). Non‐randomized studies were scored across seven domains to determine a rating of no information, critical, serious, moderate, or low risk of bias, and randomized studies were scored across five domains to determine a rating of high, some concerns, or low risk of bias. Judgments were then visualized using robvis, a tool used to create ‘traffic light’ charts for risk of bias judgments (McGuinness & Higgins, 2020). See the section on Sensitivity Analysis for testing concerning publication bias and influential studies, as well as the section on Moderator Analyses which outlines plans for the exploration of study‐ and intervention‐related characteristics as potential moderators of treatment impact.

##### Study objective 4

We assessed the quality of included qualitative studies using an adapted version of the Critical Appraisal Skills Program qualitative study checklist (CASP, 2018), making judgments on the adequacy of reporting, data collection, presentation, analysis, and validity of the conclusions drawn. The checklist is included in Supporting Information: Appendix 1. We excluded studies of particularly low quality at this stage (Noyes et al., 2019), as well as studies in which questions 1–2 on the checklist were assessed as “No.” The remaining studies were classified as high, moderate, or low quality. Each item on the checklist was coded as “yes,” “no,” or “can't tell” with respect to whether a component was adequately addressed. Using similar guidelines as presented in Templeton et al. (2017), studies were rated as high quality if they had 8–10 “yes” ratings, moderate quality with 5–7 “yes” ratings, and low quality with 4 or fewer “yes” ratings.

The results of the quality appraisal are reported in Table 10. Further, as described in the section on Sensitivity Analysis, a sensitivity analysis of qualitative evidence was performed to assess the contributions of individual studies to the synthesized process evaluation findings.

##### Study objective 5

Any study presenting discrepant or conflicting theory of change information was discussed by the research team until a decision about inclusion was reached.

##### Study objective 6

To assess methodological quality and risk of bias, in our protocol we described plans to assess studies using the Consensus on Health Economic Criteria (CHEC‐list) developed by Evers et al. (2005). For this checklist, studies are rated on a series of 19 criteria to calculate a global assessment of study quality. However, the final sample for this objective was small, with only seven studies reporting any kind of cost assessment. Of these studies, only three provided a detailed cost–benefit assessment; a majority provided very minimal cost data. Due to a lack of cost data provided and minimal cost analyses, most studies would have scored very low on the checklist, resulting in even less usable data. Given this, and the fact that the current sample was unable to be synthesized due to the low number of studies overall, formal quality assessments were not conducted.

#### 4.3.4 Measures of treatment effect

Data from each individual study were standardized so that results across studies could be meaningfully pooled. Effect size calculation was dependent on the type of data presented in each individual study. More specifically, the following two types of effect sizes calculations were used:

(a) For studies that presented dichotomous outcome measures (e.g., in the form of percentages or raw numbers representing how many participants were rearrested at least once), effect sizes were computed as odds ratios. The odds referred to the odds of recidivism (e.g., arrest, conviction, or incarceration) compared to no recidivism for an individual who participated in an aftercare/resettlement intervention relative to the odds of recidivism for an individual in the control group (Lipsey & Wilson, 2001). Specifically (Equation 1):

(1) $$\mathrm{OR}=\frac{\mathrm{ad}}{\mathrm{bc}}=\frac{P_{a}\;P_{d}}{P_{b}\;P_{c}}=\frac{P_{a}÷P_{b}}{P_{c}÷P_{d}}=\frac{P_{a}(1-p_{c})}{P_{c}(1-p_{a})},$$

where a, b, c, and d correspond to the raw frequencies of those who recidivated and those who did not for each group. For example, *a* refers to the number of youth in the treatment group who were arrested, *d* refers to the number of youth in the treatment group who were *not* arrested, *b* refers to the number of youth in the comparison group who were arrested, and *c* refers to the number of youth in the comparison group who were *not* arrested. The superscript *P* refers to the proportion in the relative cell (*a, b, c, d*) and lower‐case *p* refers to the proportion of persons in its relative group (*a* or *c*) who experienced a positive outcome (reduction in recidivism) (Lipsey & Wilson, 2001).

As odds ratios are not normally distributed, all odds ratios were log transformed to log odds ratios (LORs). LORs are centered around a value of zero, with zero indicating that recidivism is equally likely to occur in both groups. Data were coded (or reverse‐coded) such that an LOR below 0 indicates that the outcomes favor the control group (with the treatment group being more likely to recidivate), and a value above 0 indicates that the aftercare/resettlement intervention has a beneficial impact on the treatment group (a lower rate of recidivism).

The standard error of the LOR was calculated as (Equation 2):

(2) $${\mathrm{SE}}_{\mathrm{LOR}}=\sqrt{\left[\left(\frac{1}{a}\right)+\left(\frac{1}{c}\right)+\left(\frac{1}{b}\right)+\left(\frac{1}{d}\right)\right]}.$$

(b) For studies that presented means and standard deviations for both groups at post‐test, the basic standardized mean difference was calculated as the mean of the treatment group (*M*_*T*_ *)* minus the mean of the control group (*M*_*C*_ *)* divided by the pooled standard deviation (SD_pooled_) (Equation 3):

(3) $$\mathrm{Cohen}\prime s\;d=\frac{M_{T}-M_{C}}{{\mathrm{SD}}_{\mathrm{pooled}}},$$

where the pooled standard deviation was calculated by (Equation 4):

(4) $${\mathrm{SD}}_{\mathrm{pooled}}=\sqrt{\frac{{\left(n_{T}-1\right)}_{S{D^{2}}_{T}}^{2}+{\left(n_{C}-1\right)}_{S{D^{2}}_{C}}^{2}}{\left(n_{T}-1\right)+\left(n_{C}-1\right)}}.$$

In which ${\mathrm{SD}}_{T}^{2}$ is the standard deviation of the treatment group and ${\mathrm{SD}}_{C}^{2}\;$is the standard deviation of the control group.

The standard error of *d* was calculated by (Equation 5):

(5) $${\mathrm{SE}}_{d}=\sqrt{\frac{n_{T}+n_{C}}{n_{T}n_{C}}+\frac{d^{2}}{2\left(n_{T}+n_{C}\right)}}.$$

To be compatible with the effect sizes based on dichotomous data, all standardized mean difference effect sizes were converted to LORs using the Cox logit method (LOR = d*1.65).

#### 4.3.5 Unit of analysis issues

No clustered research designs were uncovered in the analytic sample of studies for objectives 1–3; as such no adjustments using intra‐cluster correlation coefficients were applied. Unit of analysis issues with respect to clustered research designs were not relevant for study objectives 4, 5, and 6.

#### 4.3.6 Criteria for determination of independent findings

##### Study objectives 1–3

All relevant effect size data were extracted from each study, and all potential effect sizes were calculated. For univariate pooled analyses, a key assumption in meta‐analysis is the independence of observations (Card, 2011). With respect to the inclusion of effect sizes for objectives 1–3, to ensure independence several a priori decision rules were defined and implemented when appropriate:

(a) If multiple reports present data from the same population or study (e.g., a dissertation and a journal article, or two separate articles using overlapping research samples), all available information will be used but the study will only be counted a single time.
(b) If a single report includes multiple experiments, they will be counted as separate studies only if the samples are completely independent and the control group is not doubly counted.
(c) If a study includes multiple treatment groups (e.g., versions of the aftercare intervention) compared to a single control group, the treatment group that is the most comparable to those in the overall set of studies will be selected for inclusion.
(d) With respect to the inclusion of effect sizes for objectives 1–3, when multiple post‐tests are reported (e.g., an immediate post‐test and a 6‐month follow‐up), the most common time point across all included studies in the set (12 months) will be chosen for the main analyses. Outcomes for all follow‐up time points will be coded, and numerous sensitivity analyses will be conducted to explore the potential impact of follow‐up time point differences.
(e) If studies report multiple outcomes that are categorized under the same outcome measure category (e.g., different measures of arrests), the most commensurate outcome to other studies will be selected.

##### Study objectives 4 and 6

For study objectives 4 and 6, if multiple reports present process evaluation or cost–benefit data from the same population or study (e.g., a dissertation and a journal article, or two separate articles used overlapping research samples), all relevant information will be coded but the study counted only once.

##### Study objective 5

For objective 5, the concept of study independence is not relevant; any information uncovered that could be used to refine the theory of change will be incorporated.

#### 4.3.7 Dealing with missing data

Substantial “missing data” was expected to be encountered throughout the coding process; specifically, across the set of included studies certain pieces of information were not presented by all study authors (e.g., sample size, participant age, participant gender). We attempted to obtain this information by contacting study authors. If missing quantitative data needed for inclusion in the meta‐analysis was not obtained from study authors, the study was excluded from quantitative analyses but was retained in summary tables and in the process evaluation and cost assessment analyses as appropriate.

#### 4.3.8 Assessment of heterogeneity

##### Study objectives 1–3

For objectives 1–3, we examined tau^2^, Cochran's *Q*‐statistics and *I* ^2^ statistics (Borenstein et al., 2017; Higgins, 2003). We also disaggregated the available outcome measures into smaller, more commensurate sets of outcomes (i.e., rearrest, reconviction, and reincarceration).^5^

##### Study objective 4

In addition to the exclusion of studies that did not fit study inclusion criteria (e.g., the evaluated program was not aftercare, participants were not youth), studies were removed if they did not evaluate program implementation or fidelity. For example, studies that were qualitative impact evaluations (e.g., where the focus was on participants' perceived outcomes) were excluded.

##### Study objective 5

Assessment of heterogeneity is not relevant for objective 5 (theory of change).

##### Study objective 6

For objective 6, as noted in the protocol we intended to assess heterogeneity by pooling outcomes based on various study and program characteristics to account for potentially variability (e.g., program setting, number of participants served, length of program operation). Unfortunately, this was not possible due to the small sample size and lack of available cost data.

#### 4.3.9 Assessment of reporting biases

For objectives 1–3, publication bias (Sterne & Harbord, 2004) and small study effects were assessed using Egger's test of small study effects and funnel plots (Egger et al., 2003; Steichen, 1998; Sterne et al., 2000).

Reporting biases are not considered relevant for study objectives 4, 5, and 6.

#### 4.3.10 Data synthesis

##### Study objectives 1–3

The two main approaches to modeling data in meta‐analysis are fixed effects and random effects models. Both models weight each study by its inverse variance, however, the calculation of weights is based on the assumed source of variability between studies (Card, 2011; Egger & Smith, 2001). A fixed effects model assumes that any between‐subject variability is the result of sampling error and occurs only by chance, while random effects models assume that between‐study heterogeneity is important and is due to factors other than random subject‐level sampling error (Card, 2011; Lipsey & Wilson, 2001). In the current analysis, given the expectation of a multitude of between‐study differences, we used restricted maximum likelihood (REML) random effects estimators with the Hartung‐Knapp‐Sidik‐Jonkman (HKSJ) variance correction. All analyses were conducted in Stata/SE 18.0, results are presented in forest plots. Analyses were conducted separately for three groups of outcomes: arrest, conviction, and incarceration.

##### Study objective 4

For outcome 4, both qualitative and quantitative data were collected to identify barriers and facilitators to program implementation and fidelity to intervention design. The extraction of qualitative data was in part based on an a priori set of expected findings, derived from a preliminary review of the literature. Specifically, broader categories of variables, such as communication, continuity of care, organization partnerships, and case management guided coding in a deductive manner wherein process data were coded within these predetermined variables either as either program “challenges” or “successes.” For reported data that did not fit into these pre‐defined variables, an inductive thematic approach was taken to code and analyze these findings using qualitative data analysis software,^6^ allowing new themes and categories to emerge.

Coding followed the three stages of aftercare/resettlement: (1) pre‐release programming and planning, including risk assessments and case management, (2) intensive transition structures and processes, including throughcare services, and (3) post‐release supervision and services, including intensive supervision and reintegration programming. However, many studies reported process data that did not belong to one of these stages; therefore, some data were coded as “overall” findings. See Table 1 for example from the coding scheme, guided by deductive and inductive variables in each of the three stages of aftercare/resettlement. A complete list of a priori variables can be found in the coding form (Supporting Information: Appendix 2).

**Table 1:** Sample coding framework.

	Framework categories		
	Pre‐release programming and planning	Intensive transition structures and processes	Post‐release supervision and reintegration
Examples of a priori variables	*Example 1*: Communication	*Example 1*: Continuity of care	*Example 1*: Contacts
Examples of emergent variables	*Example 1*: Working relationships between aftercare/resettlement workers custodial staff	*Example 1*: Basic necessities (e.g., housing)	*Example 1*: Relationships with community justice agents (e.g., parole officers)

#### 4.3.11 Subgroup analysis and investigation of heterogeneity

##### Study objectives 1–3

We investigated between‐study heterogeneity of effects through subgroup (moderator) analysis to examine whether certain categorical variables could explain some of the variability in effect sizes (Card, 2011; Lipsey & Wilson, 2001). Dichotomized variables were related to study characteristics, sample characteristics, intervention components, and program delivery. A total of 24 potential moderators were examined. Only those variables in which each subgroup had a minimum of 3 effect sizes were included in the analyses, resulting in a maximum of 16 moderators in the analyses. Associations among moderators within each of the three outcome categories were assessed in an effort to address potential collinearity; two correlations were statistically significant for the arrest outcome. These included bias rating and research design (only the bias rating was used as a moderator), and group therapy and mentoring (only the mentoring variable was used). Crosstabulations were computed between moderators within each of the four categories (study, sample, intervention, and program delivery characteristics) for each of the three program outcomes (arrest, conviction, incarceration); no significant associations were observed.

With respect to study characteristics (all identified a priori), the following four variables were examined: peer‐reviewed publication type (yes vs. no), research design (randomized control trial vs. quasi‐experimental design), treatment group sample size (<75 participants vs. 75+ participants), and risk of bias score (low/moderate risk vs. serious risk^7^).

Sample characteristics (both identified a priori) included: sample race (predominantly Caucasian/mixed race vs. predominantly racial minority^8^) and sample gender (90%+ male vs. mixed gender).

Intervention characteristics were coded for presence of the following 7 components (yes vs. no; all components were identified a priori)^9^: life skills, housing, individual therapy, group therapy, family therapy, mentoring, and substance abuse treatment.

Last, each program was coded based on who was involved in delivery of program services, including the following three non‐mutually exclusive options^10^ (identified a priori): probation officers, program staff, and community service providers.

Subgroup analyses were conducted using the analog to the ANOVA method, which separates the total variability (*Q*_T_) into the within‐group variation (*Q*_w_ = the summed *Q*‐statistics for each of the two groups in the analysis) and that which can be explained by the categorical variable (the between‐group variation; *Q*_b_ = the difference between the total and within‐Q‐statistics). A statistically significant *Q*_b_ suggests the two categories are producing significantly different effect sizes and the difference is due to more than sampling error (Lipsey & Wilson, 2001).

In addition, random effects meta‐regression models were implemented, incorporating multiple moderators into the same model (Hedges & Olkin, 1985). This approach served to reduce potential confounding across moderators. As in standard linear regression analysis, the coefficients derived from a meta‐regression describe how the dependent variable changes with a unit increase in the predictor (moderator) variables. We considered the use of robust variance estimation (RVE) so that dependent effect sizes could be included in the meta‐regression models without violating the assumption of independence (see Hedges et al., 2010; Pustejovsky & Tipton, 2021; Tanner‐Smith & Tipton, 2013). However, based on simulations provided by Hedges et al. (2010) and Tipton (2013), RVE appeared unlikely to perform well (with respect to empirical coverage) for the given number of effect sizes per study and total number of studies in our set. As such, standard meta‐regression models were implemented using no more than one effect size per study. We used REML to estimate the additive component of tau^2^, with the Knapp Hartung variance correction (Tipton et al., 2019a, 2019b). Given the relatively small number of studies for each outcome, moderator variables were grouped into three separate models for the outcomes of arrest and conviction, and two separate models for the outcome of incarceration.

##### Study objective 4

Subgroup analysis of qualitative studies was conducted based on themes uncovered from the process evaluation evidence synthesis for aftercare/resettlement programming as a whole, as well as for each of the three stages of aftercare/resettlement. Specifically, overarching themes and findings were derived, along with subgroup analyses based on findings related to the custodial phase of intervention, the transition phase, and the post‐custodial phase.

##### Study objective 5

Subgroup analysis and investigation of heterogeneity is not relevant for objective 5 (theory of change).

##### Study objective 6

Subgroup analysis of intervention cost‐related data was intended to be analyzed in smaller sets based on various characteristics, such as intervention setting (e.g., for those programs delivered in a hybrid prison/community setting than solely in the community), program size (e.g., low vs. high numbers of participants served), program length of operation (e.g., newer vs. more established programs), geographic location (e.g., United States vs. United Kingdom). However, the sample included only seven eligible studies (with 8 independent program sites) and a lack of consistency in reported data, which precluded any subgroup analysis.

#### 4.3.12 Sensitivity analysis

##### Study objectives 1–3

For objectives 1–3, we tested sensitivity of the findings to strongly influential studies by conducting a remove‐one‐study influence analysis (Tobias, 1999). To do so, each study in the meta‐analysis was omitted, one at a time, and the pooled effect was recalculated without that study to determine whether its removal had a notable impact on the pooled findings of the meta‐analysis. Further, we tested the sensitivity of findings by using research design bias as a treatment moderator, and calculating the pooled effect separately for low/moderate risk versus serious risk studies.

##### Study objective 4

A sensitivity analysis of the synthesized process evaluation findings was performed to assess the contributions of qualitative studies. By examining both the “frequency” (i.e., if certain conclusions are reliant on data from a single study) and “thickness” (i.e., the depth of understanding that studies bring to conclusions) of study outcomes within the qualitative evidence synthesis findings, we considered the influence and profile of individual studies (i.e., if studies with low or high methodological quality were more influential), and possible biases that shaped the synthesized findings (Carroll et al., 2012, 2013).^11^

In addition, the CASP (2018) qualitative study quality appraisal checklist was used as a guide while conducting the sensitivity analysis (see Supporting Information: Appendix 1). The level of quality observed among qualitative studies (high, moderate, or low in methodological quality, based on a cut off score modeled after Templeton et al. (2017), was used to examine study contributions to synthesized process evaluation findings. Where conclusions were based primarily on studies with low or moderate methodological quality, we tempered our conclusions appropriately, or removed themes altogether.

##### Study objectives 5 and 6

Sensitivity analysis is not relevant for objective 5 (theory of change) or objective 6 (intervention cost).

#### 4.3.13 Treatment of qualitative research

For objective 4, the study synthesized qualitative data by drawing on thematic analysis and framework synthesis (Carroll et al., 2011; Dixon‐Woods, 2011; Thomas & Harden, 2008). Following a systematic search of the literature (using electronic databases and gray literature sources), retrieved qualitative studies were read and re‐read by two independent reviewers, and findings were coded into variables based on the initial framework of the aftercare/resettlement model. Further, as new themes emerged that were not decided a priori, the framework was updated to represent the emerging synthesis. Lastly, coded data were analyzed in NVivo, where themes emerged. As in previous studies utilizing framework synthesis (e.g., Abbott et al., 2019), the goal herein is to present an understanding of the effects of aftercare and resettlement programming as well as an explanation of the mechanisms that work to produce these effects.

## 5 RESULTS

### 5.1 Description of studies

#### 5.1.1 Results of the search

**Figure 2:**
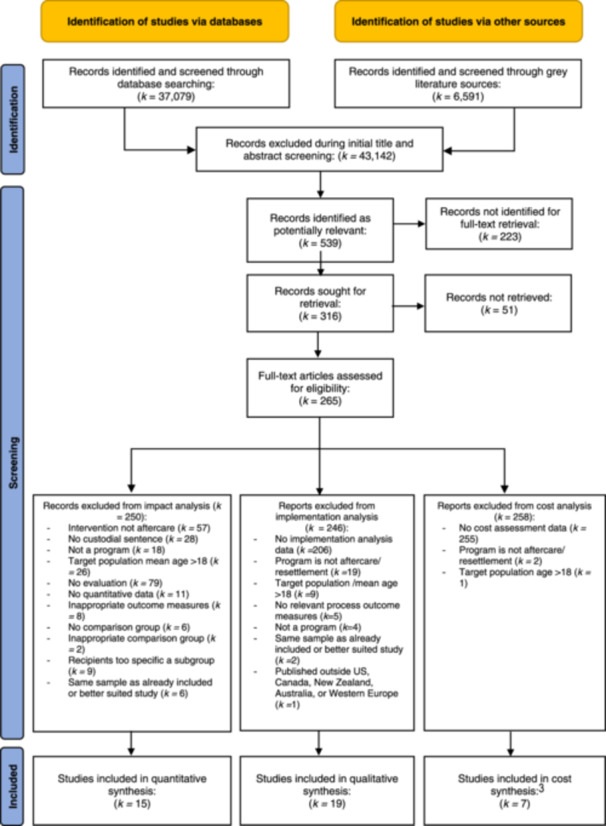
Search process flow diagram.

The systematic search resulted in a total of 37,079 initial hits across the 26 electronic databases and an additional 6591 from gray literature searching (e.g., relevant websites, open access search engines, journal searches).^12^ The review of the titles and abstracts of these initial hits resulted in 528potentially eligible studies (after the removal of duplicates), and 307 were identified for full text retrieval. A total of 248 documents were retrieved in full for the application of inclusion and exclusion criteria; 59 documents were unretrievable (these were largely conference presentations or student theses/dissertations). This process resulted in 15 intervention studies selected for inclusion, representing 21 independent implementation sites (i.e., some studies had multiple sites) and producing 35 independent effect sizes. See Figure 2 for a review of the search results.

As per the protocol under “Criteria for determination of independent findings,” five a priori decision rules were defined for the intervention studies. After completing the search and applying inclusion criteria, two of these rules were not necessary in the current study (“b” and “c”). The results of the application of rules to the results of the systematic search and included studies are as follows:

(a) If multiple reports present data from the same population or study (e.g., a dissertation and a journal article, or two separate articles using overlapping research samples), all available information will be used but the study will only be counted a single time.
  - Seven^13^ research reports fell under this rule and were not included due to overlapping samples.
(b) If a single report includes multiple experiments, they will be counted as separate studies only if the samples are completely independent and the control group is not doubly counted.
  - Not relevant to the current study.
(c) If a study includes multiple treatment groups (e.g., versions of the aftercare intervention) compared to a single control group, the treatment group that is the most comparable to those in the overall set of studies will be selected for inclusion.
  - Not relevant to the current study.
(d) With respect to the inclusion of effect sizes for objectives 1–3, when multiple post‐tests are reported (e.g., an immediate post‐test and a 6‐month follow‐up), the most common time point across all included studies in the set (12 months) will be chosen for the main analyses. Outcomes for all follow‐up time points will be coded, and numerous sensitivity analyses will be conducted to explore the potential impact of follow‐up time point differences.
  - Three included studies reported multiple time points for the measurement of outcomes; results of sensitivity analyses are reported in the “Quantitative Synthesis of Results” section.
(e) If studies report multiple outcomes that are categorized under the same outcome measure category (e.g., different measures of arrests), the most commensurate outcome to other studies will be selected.
  - Bouffard and Bergseth (2008): reported on 4 different measures of police contacts/arrests that were based on slightly different definitions and represented overlapping data (e.g., # of new court contacts per week at risk and # of new court contacts); only one was used.
  - Iutcovich and Pratt (1998) Allegheny: reported on both arrest count and mean # of arrests, only one was used.
  - Iutcovich and Pratt (1998) Philadelphia: reported on both arrest count and mean # of arrests, only one was used.
  - Sontheimer and Goodstein (1993): reported on both total arrests and frequency of arrests over entire time at risk; only one was used.

With respect to the implementation study search, a total of 19 studies, with 27 independent program sites, were selected for inclusion.

Last, the search resulted in a total of 10 studies that presented cost data; after further exclusions, seven studies (8 program sites) remained for the final sample.

#### 5.1.2 Included studies

##### Intervention studies

The present sample included 15 eligible studies assessing intervention impact. See Table 2 for a detailed summary of each study.

**Table 2:** Intervention study characteristics.

Author (date)	Program site	Program name	Location	Pub. type	Research design	Treatment sample (baseline)	Comparison sample (baseline)	Age range (mean)	Sample gender	Post‐test timing	Outcomes
Abrams et al. (2008)	Midwest USA	Transitional Living Program (TLP)	USA	Journal article	Quasi‐experiment with weakly matched comparison group	Youth released from a correctional facility after completing the TLP (*n* = 46)	Offenders released from a correctional facility who did not participate in the TLP (*n* = 15)	15.9^b^	All male	12 months	Conviction
Barton et al. (2008)	Alaska	Boys and Girls Clubs of America (BGCA) Targeted Re‐Entry (TR)	USA	Report	Quasi‐experiment with matched comparison group	Youth released from a custodial facility who participated in the program (*n* = 84)	A retrospective comparison group drawn from years prior to the introduction of systematic transition programming (*n* = 64)	13–19 (17.3)	Majority male	12 months	Arrest and Conviction
	Arkansas	Boys and Girls Clubs of America (BGCA) Targeted Re‐Entry (TR)	USA	Report	Quasi‐experiment with matched comparison group	Youth released from a custodial facility who participated in the program (*n* = 83)	Contemporary matched comparison group of those not participating in program (*n* = 89)	12–19 (16.2)	Majority male	12 months	Conviction
	Wisconsin	Boys and Girls Clubs of America (BGCA) Targeted Re‐Entry (TR)	USA	Report	Quasi‐experiment with matched comparison group	Youth released from a custodial facility who participated in the program (*n* = 81)	Contemporary matched comparison group of those not participating in the program (*n* = 50)	14–20 (16.7)	All male	12 months	Arrest and Conviction
Bouffard and Bergseth (2008)	Clay County	Clay County Reentry Services Project (RSP)	USA	Journal article	Quasi‐experiment with weakly matched comparison group	Youth returning from 3+ weeks of custodial placement (*n* = 63)	Youth in a neighboring county returning from 3+ weeks of placement on probation without reentry services (*n* = 49)	11–19 (16.5)	Majority male	6 months	Arrest
Calleja (2016)	Wayne County	Wayne County Second Chance Reentry (WC‐SCR) Program	USA	Journal article	Quasi‐experiment with weakly matched comparison group	Youth placed in residential facilities (*n* = 117)	Youth receiving 6 months of treatment‐as‐usual aftercare (*n* = 156)	13–18 (15.9)	All male	24 months	Conviction
Cillo (2001)	Westchester County	Westchester County Aftercare Program (WCAP)	USA	Thesis/dissertation	Randomized Controlled Trial	Youth returning to non‐foster care homes after incarceration at Jenny Clarkson Residential Adolescent Facility (*n* = 50)	Adolescents returning home on traditional probation (*n* = 50)	11–17 (15.2)	Majority male	3 months	Arrest
Greenwood et al. (1993)	Detroit	The Skillman Aftercare Experiment	USA	Report	Randomized Controlled Trial	Male offenders returning home after a placement in a residential correctional program (*n* = 50)	Male youth on regular forms of post‐release care (*n* = 49)	17.0^b^	All male	12 months	Conviction
Hawkins et al. (2009)	Colorado, Florida, Kansas, South Carolina^a^	The Serious and Violent Offender Reentry Initiative (SVORI)	USA	Report	Quasi‐experiment with matched comparison group	SVORI youth released into the community after custodial placement (*n *= 152)	Youth released into the community after residential placements who received treatment‐as‐usual (*n* = 185)	17.0^b^	All male	15 months	Incarceration
Hazel et al. (2012)	North West England	North West Resettlement Consortium	England	Report	Quasi‐experiment with matched comparison group	Youth with a detention and training order sentence who received the “enhanced offer” (*n* = 168)	Youth with a detention and training order sentence, released from custody in the year previous to the cohort receiving the enhanced offer (*n* = 104)	15–17 (16.4)	All male	3 months	Conviction
Iutcovich and Pratt (1998)	Allegheny	Abraxas Non‐Residential Care (Abraxas NRC)	USA	Report	Quasi‐experiment with weakly matched comparison group	Youth released to the Abraxas NRC program post‐residential placement (*n *= 72)	Youth released from residential placement receiving aftercare services directly through the Probation Department (*n* = 58)	17.0^b^	Majority male	12, 18 months	Arrest and Incarceration
	Philadelphia	Philadelphia Opportunities Industrialization Center (Youth Advocacy Plus Program (OIC‐YAPP)	USA	Report	Quasi‐experiment with weakly matched comparison group	Youth released to the OIC‐YAPP program post residential‐ placement (*n *= 87)	Youth released from residential placement receiving aftercare services directly through the Probation Department (*n* = 85)	17.0^b^	All male	12, 18 months	Arrest and Incarceration
Liberman et al. (2021)	Oklahoma	Second Chance Act Reentry program	USA	Report	Quasi‐experiment with matched comparison group	Youth returning home after at least a 6‐month stay in custody (*n* = 153)	Historical cohort sample of youth returning home after release (*n* = 111)	12–19 (16.3)	Majority male	12 months	Conviction
	Virginia	Second Chance Act Reentry program	USA	Report	Quasi‐experiment with matched comparison group	Youth returning home after at least a 6‐month stay in custody (*n* = 217)	Youth returning home after at least 6 months confinement and not enrolled in program (*n* = 286)	13–19.9 (16.6)	Majority male	12 months	Arrest, Conviction, and Incarceration
Sontheimer and Goodstein (1993)	Philadelphia	Intensive Aftercare Probation Program	USA	Journal article	Randomized Controlled Trial	High risk males released from the Bensalem Youth Development Denter and randomly assigned to the program (*n* = 44)	High risk males released from the Bensalem Youth Development Center and randomly assigned to receive standard probation (*n* = 46)	17.2^b^	All male	3 months	Arrest
Stafford and Glassner (2012)	Texas	Children's Aftercare Reentry Experience (CARE)	USA	Report	Quasi‐experiment with weakly matched comparison group	Youth enrolled in CARE within 90 days of release from a residential facility (*n* = 317)	Youth parolees released from a residential facility between July 2007 and December 2009 (*n* = 533)	16.5^b^	Majority male	12 months	Arrest
Andersson Vogel et al. (2014)	Sweden	Coherent chain of Care (CoC project)	Sweden	Journal article	Quasi‐experiment with matched comparison group	Youth released from 2+ weeks in care in eligible municipalities (*n* = 156)	Youth released from 2+ weeks in care in eligible municipalities not assigned to the program (*n* = 335)	11–21^c^	Majority male	24 months	Conviction and Incarceration
Wiebush et al. (2005)	Colorado	Intensive Aftercare Program (IAP)	North America	Report	Randomized Controlled Trial	High‐risk males released from state custody (*n* = 82)	High‐risk males released from state custody and not assigned to the program (*n* = 68)	Not reported	All male	12 months	Arrest, Conviction, and Incarceration
	Nevada	Intensive Aftercare Program (IAP)	North America	Report	Randomized Controlled Trial	High‐risk males released from state custody (*n* = 120)	High‐risk males released from state custody and not assigned to the program (*n* = 127)	Not reported	All male	12 months	Arrest, Conviction, and Incarceration
	Virginia	Intensive Parole Program (IPP)	North America	Report	Randomized Controlled Trial	High‐risk males released from state custody (*n* = 74)	High‐risk males released from state custody and not assigned to the program (*n* = 44)	Not reported	All male	12 months	Arrest, Conviction, and Incarceration
Wright et al. (2012)	South West England	South West Resettlement Consortium	England	Report	Quasi‐experiment with matched comparison group	Youth who received the “enhanced offer” and were released during the 19 month period from September 2010 to March 2012 (*n* = 82)	Youth released from custody in the year previous to the cohort receiving the enhanced offer (*n* = 58)	14–17 (16.2)	Majority male	3 months	Arrest

^a^ Participants were pooled across program sites; individual sites were not separated for analyses presented.

^b^ Age range not reported.

^c^ Mean age not reported.

These 15 studies represented 21 individual program implementation sites^14^ and contributed a total of 35 effect sizes assessing three outcomes: arrest (*k* = 14), conviction (*k* = 13), and incarceration (*k* = 8). Ten studies contributed one effect size, while the remaining five studies contributed two or more effect sizes. A total of 2300 treatment group and 2562 comparison group participants were included at baseline. No studies focused specifically on outcomes of violence were identified in the search, and all outcomes were measured using official or administrative data. Of the 21 program sites, 15 (71%) were detailed in government or organizational reports, 5 (24%) were in published journal articles, and 1 (5%) was an unpublished thesis. Publication year ranged from 1993 to 2021, with 62% (*k* = 13) published in 2006 or later. Included studies were almost exclusively conducted in the United States, with only 3 (14%) conducted in Western Europe.

With respect to program characteristics, the vast majority of interventions (*k* = 19, 90%) were implemented both pre‐ and post‐release, and were primarily handled by a case manager (*k* = 20, 95%). Programs included additional involvement from other program staff (62%), community‐based service providers (71%), probation officers (29%), and counselors (10%; categories were not mutually exclusive). Specific program components included behavioral or emotional supports (76% of programs), academic supports (71%), vocational training (76%), life skills development (62%), housing assistance (48%), individual therapy (67%), group therapy (14%), family therapy or services (62%), mentoring (43%), substance use treatment (71%), and parental involvement (86%).

A total of 6 (29%) sites were evaluated using a randomized controlled trial, while the remaining 15 (71%) utilized a non‐randomized design with a matched comparison group. Of the 21 sites, 57% were scored as having low/moderate research design bias, while 43% were scored as having serious bias concerns. Outcomes were most commonly measured between 12 and 17 months post release (57%). Study samples were primarily male, with 8 (38%) utilizing all male samples, as opposed to mixed gender samples (62%). Overall participant age ranged from 11 to 21 years, with means ranging from 15.2 to 17.3. Samples primarily included participants of an ethnic minority group (i.e., <30% White; 62%). Sample size at baseline ranged from 61 to 850, with a mean of 235 (SD = 190.2); 57% of the sites had a sample of over 75 participants. See Table 3 for a presentation of study characteristics for the full sample, as well as by outcome measure.

**Table 3:** Intervention study characteristics.

Type	Characteristic	Total sample, *N* (%)	Arrests, *N* (%)	Conviction, *N* (%)	Incarceration, *N* (%)
Study characteristics	Publication type				
	Journal article	5 (24%)	2 (14%)	3 (23%)	1 (12%)
	Report	15 (71%)	11 (79%)	10 (77%)	7 (88%)
	Thesis/dissertation	1 (5%)	1 (7%)	0	0
	Peer review				
	No	16 (76%)	12 (86%)	10 (77%)	7 (88%)
	Yes	5 (24%)	2 (14%)	3 (23%)	1 (12%)
	Publication year				
	1993–2005	8 (38%)	8 (57%)	4 (31%)	5 (63%)
	2006–2021	13 (62%)	6 (43%)	9 (69%)	3 (37%)
	Location				
	North America	18 (86%)	13 (93%)	11 (85%)	7 (88%)
	Europe	3 (14%)	1 (7%)	2 (15%)	1 (12%)
	Research design				
	Non‐RCT	15 (71%)	8 (57%)	9 (69%)	5 (63%)
	RCT	6 (29%)	6 (43%)	4 (31%)	3 (37%)
	Bias score				
	Low/moderate risk	12 (57%)	9 (64%)	8 (62%)	5 (63%)
	Serious/critical risk	9 (43%)	5 (36%)	5 (38%)	3 (37%)
	Tx group sample size				
	<75	9 (43%)	8 (57%)	4 (31%)	3 (37%)
	75+	12 (57%)	6 (43%)	9 (69%)	5 (63%)
	Participant mean age	*M* = 16.5	*M* = 16.7	*M* = 16.5	*M* = 16.9
		SD = 0.55	SD = 0.59	SD = 0.47	SD = 0.20
	Participant age range	11–21	11–20	11–21	11–21
	Sample ethnicity				
	White/mixed	8 (38%)	5 (36%)	6 (46%)	3 (37%)
	Minority	13 (62%)	9 (64%)	7 (54%)	5 (63%)
	Sample gender				
	All male	8 (38%)	6 (43%)	4 (31%)	3 (37%)
	Mixed gender	13 (62%)	8 (57%)	9 (69%)	5 (63%)
Program components	Behavioral/emotional skills				
	No	3 (14%)	3 (21%)	0	2 (25%)
	Yes	16 (76%)	10 (71%)	11 (85%)	4 (50%)
	Missing	2 (10%)	1 (7%)	2 (15%)	2 (25%)
	Academic supports				
	No	3 (14%)	2 (14%)	2 (15%)	0
	Yes	15 (71%)	11 (79%)	8 (62%)	6 (75%)
	Missing	3 (14%)	1 (7%)	3 (23%)	2 (25%)
	Vocational training				
	No	3 (14%)	2 (14%)	2 (15%)	0
	Yes	16 (76%)	11 (79%)	9 (69%)	6 (75%)
	Missing	2 (10%)	1 (7%)	2 (15%)	2 (25%)
	Life skills				
	No	6 (29%)	5 (36%)	2 (15%)	2 (25%)
	Yes	13 (62%)	8 (57%)	9 (69%)	4 (50%)
	Missing	2 (10%)	1 (7%)	2 (15%)	2 (25%)
	Housing				
	No	10 (48%)	8 (57%)	8 (62%)	3 (37%)
	Yes	10 (48%)	6 (43%)	4 (31%)	5 (63%)
	Missing	1 (5%)	0	1 (8%)	0
	Individual therapy				
	No	5 (24%)	3 (21%)	4 (31%)	1 (12%)
	Yes	14 (67%)	10 (71%)	7 (54%)	6 (75%)
	Missing	2 (10%)	1 (87)	2 (15%)	1 (12%)
	Group therapy				
	No	15 (71%)	10 (71%)	7 (54%)	3 (37%)
	Yes	3 (14%)	3 (21%)	3 (23%)	3 (37%)
	Missing	3 (14%)	1 (7%)	3 (23%)	2 (25%)
	Family therapy				
	No	6 (29%)	4 (29%)	2 (15%)	0
	Yes	13 (62%)	10 (71%)	9 (69%)	7 (88%)
	Missing	2 (10%)	0	2 (15%)	1 (12%)
	Mentoring				
	No	9 (43%)	6 (43%)	6 (46%)	4 (50%)
	Yes	9 (43%)	7 (50%)	4 (31%)	2 (25%)
	Missing	3 (14%)	1 (7%)	3 (23%)	2 (25%)
	Substance use treatment				
	No	5 (24%)	4 (29%)	2 (15%)	0
	Yes	15 (71%)	10 (71%)	10 (77%)	7 (88%)
	Missing	1 (5%)	0	1 (8%)	1 (12%)
	Parental involvement				
	No	2 (10%)	0	2 (15%)	1 (12%)
	Yes	18 (86%)	14 (100%)	11 (85%)	6 (75%)
	Missing	1 (5%)	0	0	1 (12%)
Program delivery	Counselors				
	No	19 (90%)	12 (86%)	13 (100%)	8 (100%)
	Yes	2 (10%)	2 (14%)	0	0
	Probation officers				
	No	15 (71%)	8 (57%)	10 (77%)	6 (75%)
	Yes	6 (29%)	6 (43%)	3 (23%)	2 (25%)
	Case managers				
	No	1 (5%)	1 (7%)	0	0
	Yes	20 (95%)	13 (93%)	13 (100%)	8 (100%)
	Program staff				
	No	8 (38%)	5 (35%)	2 (15%)	2 (25%)
	Yes	13 (62%)	9 (64%)	11 (85%)	6 (75%)
	Community service providers				
	No	6 (29%)	3 (21%)	4 (31%)	1 (12%)
	Yes	15 (71%)	11 (79%)	9 (69%)	7 (88%)
	Timing of services				
	Pre‐release only	1 (5%)	0	1 (8%)	0
	Post‐release only	1 (5%)	1 (7%)	0	0
	Pre‐ and post‐release	19 (90%)	13 (93%)	12 (92%)	8 (100%)

##### Implementation studies

The systematic search resulted in a total of 19 publications which provided process evaluations of 18 reentry programs in 27 program sites.^15^ Two studies, Lindquist et al. (2014) and McKay et al. (2014), reported data on the same program, data, and samples; these two publications were treated as a single study for the purposes of the thematic analysis, sensitivity analysis, and heterogeneity assessment. Conversely, four studies presented separate process findings for programs that had been implemented in multiple sites (Barton et al., 2008; Iutcovich & Pratt, 1998; Liberman et al., 2021; Wiebush et al., 2005). Where one publication presented findings for multiple sites (and where samples were independent), each site was treated as a separate study. See Table 4 for an overview of each included study.

**Table 4:** Implementation study characteristics.

Author(s), year	Program name	Implementation site (if applicable)	Sample	Data sources
Abrams et al. (2008)	Transitional Living Program (TLP)	United States	10 youth participants, 5 TLP staff	Qualitative interviews
Barton et al. (2008)	The Boys and Girls Clubs of America (BGCA) Targeted Re‐Entry (TR)	Alabama	Program staff, correctional facility personnel, members of partnering organizations. Ns not reported.	Qualitative interviews, program materials, program case records
		Alaska	Program staff, correctional facility personnel, members of partnering organizations. Ns not reported.	Qualitative interviews, program materials, program case records
		Arkansas	Program staff, correctional facility personnel, members of partnering organizations. Ns not reported.	Qualitative interviews, program materials, program case records
		Wisconsin	Program staff, correctional facility personnel, members of partnering organizations. Ns not reported.	Qualitative interviews, program materials, program case records
Citizens' Committee for Children of New York (2000)	Juvenile Services Aftercare Program	New York	8 aftercare workers	Qualitative interviews, questionnaires
Dum and Fader (2013)	Powelton Aftercare Program	Pennsylvania	25 aftercare workers	Qualitative interviews, observations
Flynn et al. (2003)	Network Aftercare System	Alabama	5 program administrators (interviews), focus group sample size not reported	Qualitative interviews, program case records, focus groups, observations
Hazel and Hampson (2015)	Resettlement Broker Project	North Wales	11 young people, 3 parents, 2 policymakers, 11 practitioners	Qualitative interviews, questionnaires, focus groups
Hazel et al. (2008)	RESET Program	United Kingdom	63 stakeholders, including front‐line workers and their managers, local and strategic‐level stakeholders, 28 young people, 4 parents	Qualitative interviews, program materials, focus groups, observations
Hazel et al. (2012)	North West Resettlement Consortium	North West England	Young people, stakeholders. Ns not reported.	Qualitative interviews, questionnaires
Ipsos MORI (2012)	London Youth Reducing Reoffending Program (Daedalus)	United Kingdom	23 young people, 26 policy stakeholders, 2 discussion groups of case managers, 6 Youth Offending Team workers, 5 family interviews	Qualitative interviews, program case records, questionnaires, focus groups, observations
Iutcovich and Pratt (1998)	Pennsylvania Intensive Aftercare Programs	Allegheny	Not reported	Qualitative interviews, program materials, program case records, questionnaires
		Philadelphia	Not reported	Qualitative interviews, program materials, program case records, questionnaires
Jain et al. (2018)	Juvenile Reentry Program	California	25 case managers (focus group), 15 key stakeholders (interviews), 75 survey respondents	Qualitative interviews, program materials, questionnaires focus groups, observations
Liberman et al. (2021)	Second Chance Act (SCA) reentry program	California	Parents, program administrators, community partners. Sample sizes not reported.	Qualitative interviews, focus groups, observations
		Oklahoma	128 youth; Sample sizes for parents, program administrators, and community partners not reported.	Qualitative interviews, focus groups, observations
		Texas	Parents, program administrators, community partners. Sample sizes not reported.	Qualitative interviews, focus groups, observations
		Virginia	127 youth; Sample sizes for parents, program administrators, and community partners not reported.	Qualitative interviews, focus groups, observations
Lindquist et al. (2014); McKay et al. (2014)	Tribal Juvenile Detention and Reentry Green Demonstration (“Green Reentry”) programs	Three tribes: the Rosebud Sioux Tribe, the Hualapai Indian Tribe, and the Mississippi Band of Choctaw Indians Tribe. Findings presented together.	77 staff and stakeholders, 56 youth, 41 parents (individual interviews), 32 elders, 17 parents (focus groups)	Qualitative interviews, questionnaires, focus groups, observations
Miller and MacGillivray (2002)	Youth Offender Demonstration projects	United States	Participants, case managers, community partners. Sample sizes not reported.	Qualitative interviews, program materials, observations
Sinclair et al. (2021)	Model Demonstration Project	United States	8 youth, 4 transition specialists	Qualitative interviews
Andersson Vogel et al. (2014)	The Leaving Care Project	Sweden	9 case managers	Qualitative interviews, questionnaires
Wiebush et al. (2005)	Intensive Aftercare Program (IAP)	Colorado	IAP coordinators, program staff, technical assistance providers. Sample sizes not reported. 67 treatment group youth and 51 control group youth.	Qualitative interviews, program materials, program case records, observations
		Nevada	IAP coordinators, program staff, technical assistance providers. Sample sizes not reported. 100 treatment group youth and 120 control group youth.	Qualitative interviews, program materials, program case records, observations
		Virginia	IAP coordinators, program staff, technical assistance providers. Sample sizes not reported. 63 treatment group youth and 34 control group youth.	Qualitative interviews, program materials, program case records, observations
Wright et al. (2012)	South West Resettlement Consortium	South West England	Young people. Stakeholders. Ns not reported.	Qualitative interviews, questionnaires

As shown in Table 5, studies were published between 1998 and 2021 (median = 2012), primarily in the United States. Data were commonly collected through surveys and interviews with participants or program staff (e.g., aftercare/resettlement workers, case managers, stakeholders), observations and field notes, focus groups, and/or case records. Qualitative interviewing was used as a method of data collection in all 27 implementation sites. Further, observations (*k* = 14), surveys of program materials (*k* = 12) and case records (*k* = 11), focus groups (*k* = 10), and questionnaires (*k* = 10) were used to collect data. Methods of data analysis in the 19 studies were varied and often not stated explicitly, but included ethnographic analysis, narrative synthesis, and thematic analysis. Last, in the 27 implementation sites, the most common sources for data were program administrators (*k* = 18) and case managers (18). Other sources included community partners (*k* = 13), participants (*k* = 7), parents (6), custodial staff (*k* = 4), probation officers (*k* = 3), school staff (*k* = 2), counselors (*k* = 1), and policymakers (*k* = 1).

**Table 5:** Implementation study descriptives.

Characteristic	*N* (%)
Publication type (*k =* 19)^1^	
Journal article	6 (32%)
Report	13 (68%)
Peer review (*k =* 19)^1^	
Yes	6 (32%)
No	13 (68%)
Publication year (*k =* 19)^1^	
1993–2005	5 (26%)
2006–2021	14 (74%)
Program location (*k =* 18)^2^	
North America	12 (67%)
Europe	6 (33%)
Timing of services (*k =* 18)^2^	
Pre‐ and post‐release	16 (89%)
Missing	2 (11%)
Data collection methods (*k =* 27 implementation sites)	
Qualitative interviews	27 (100%)
Program materials	12 (44%)
Program case records	11 (41%)
Questionnaires	10 (37%)
Focus groups	10 (37%)
Observations	14 (52%)
Research design (*k =* 19)^1^	
Qualitative	9 (47%)
Mixed method	10 (53%)
Critical appraisal quality assessment (*k =* 19)^1^	
High quality	12 (63%)
Moderate quality	7 (37%)

^1^ There were 19 publications included in the synthesis of process findings, including two publications that reported on the same program (Lindquist et al., 2014; McKay et al., 2014).

^2^ As two publications reported on the same program, only 18 programs were included in the process synthesis.

##### Cost assessment studies

A total of 7 studies (8 program sites) were found to report on cost assessment data. Of these, 3 assessed a program implemented in the United States, 1 in Canada, and 3 in the United Kingdom. Three assessments were reported in journal articles, with 4 in reports. See Table 6 for an overall summary of the studies.

**Table 6:** Cost assessment study characteristics.

Author (year)	Program name	Location	Publication type	Assessment reported
Beausoleil et al. (2017)	Redemption Reintegration Services (RRS)	Canada	Journal article	Compared cost of service usage between treatment and comparison groups
Bergseth and McDonald (2007)	Reentry Services Project (RSP)	USA	Journal article	Cost‐benefit analysis comparing program impact to costs related to justice system processing across treatment and comparison group youth
Cowell et al. (2010)	Serious and Violent Offender Reentry Initiative (SVORI)	USA	Report	Cost‐benefit analysis assessing costs of pre‐release services
Greenwood et al. (1993)	The Skillman Aftercare Experiment	USA	Report	Compared costs of providing aftercare services via treatment program with standard aftercare services
Hazel et al. (2012)	North West Resettlement Consortium	United Kingdom	Report	Cost‐benefit analysis assessing costs related to reoffending, housing, and education of program youth
Renshaw (2007)	RESET	United Kingdom	Journal article	Provided estimated costs and savings with associated estimates in reduced offending
Wright et al. (2012)	South West Resettlement Consortium	United Kingdom	Report	Cost‐benefit analysis assessing costs related to reoffending, housing, and education of program youth

#### 5.1.3 Excluded studies

##### Intervention studies

The 250 studies excluded from the analysis of intervention evaluation data are summarized in Table 7, with detailed exclusion reasons. Of these 250 studies, the majority were excluded for not having an evaluation of a program (*k* = 79) or because the program discussed was not an aftercare or resettlement program (*k* = 57). Many studies were “near misses”; for example, where samples were a mix of youth with custodial sentences and those without but data were not presented separately (e.g., Lane et al., 2005; Lindquist et al., 2014; Savage et al., 2010). Similarly, some studies had treatment samples with mean ages that were between 19 and 20 years old, just above the cutoff of 18 years (e.g., Beausoleil et al., 2017; Josi & Sechrest, 1999).

**Table 7:** Characteristics of studies excluded from intervention analysis (*k* = 250).

Exclusion reasons	Number of studies excluded
Intervention is not aftercare	57 (22.8%)
Participants did not have a custodial sentence	28 (11.2%)
Not a program	18 (7.2%)
Target population not youth/mean age >18	26 (10.4%)
No evaluation	79 (31.6%)
No quantitative data	11 (4.4%)
Inappropriate outcome measures	8 (3.2%)
No comparison group	6 (2.4%)
Inappropriate comparison group	2 (0.8%)
Participants too specific a subgroup	9 (3.6%)
Same sample as already included/better suited study	6 (2.4%)

##### Implementation studies

Nineteen of the 265 full text publications were included in the qualitative synthesis analysis of implementation data, and 212 were excluded for not having relevant implementation data. An additional 40 studies were excluded for not meeting other study eligibility requirements, as summarized in Table 8.

**Table 8:** Excluded implementation evaluation studies (*k* = 246).

Exclusion reasons	Number of studies excluded
No implementation analysis data	206 (83.7%)
Program is not aftercare/resettlement	19 (7.7%)
Target population not youth/mean age >18	9 (3.7%)
No relevant process outcomes	5 (2%)
Not a program	4 (1.6%)
Same sample as already included/better suited study	2 (0.8%)
Published outside US, Canada, New Zealand, Australia, or Western Europe	1 (0.4%)

##### Cost studies

Of the 265 retrieved studies, 258 were excluded from the synthesis of cost assessment data. 23 were excluded for not reporting any appropriate cost assessment data, and an additional 3 were excluded as they did not meet program eligibility requirements (e.g., sample mean age, non‐commensurate program), despite reporting on relevant cost data. See Table 9.

**Table 9:** Excluded cost assessment studies (*k* = 241).

Exclusion reasons	Number of studies excluded
No cost assessment data	255 (98.8%)
Program is not aftercare/resettlement	2 (0.8%)
Target population not youth/mean age >18	1 (0.4%)

#### 5.1.4 Studies awaiting classification

Some studies were flagged for retrieval and further assessment but associated texts or study details could not be located; references to these studies are listed in the “studies awaiting classification” reference list. Generally, these entries were conference presentations that had no associated texts, or dissertations/theses that could not be accessed or located by our InterLibrary Loan service. The characteristics of all studies awaiting classification are listed in Supporting Information: Appendix 4.

#### 5.1.5 Risk of bias in included studies

##### Intervention studies

The included intervention studies consisted of 4 RCT designs (6 program sites), and 11 non‐randomized designs (15 program sites). The RCTs were assessed for risk of bias and overall study quality using the Revised Cochrane Risk of Bias tool for randomized trials (RoB‐2; Higgins et al., 2019), while the non‐randomized studies were assessed the Cochrane Risk of Bias in Non‐randomized Studies of Interventions (ROBINS‐I; Sterne et al., 2016). With respect to the RCT designs, risk of bias was assessed across five domains relating to the randomization process, deviations from the intended intervention, missing outcome data, outcome measurement, and selection of the reported result. All six of the reported program sites received an overall judgment of “some concerns” indicating moderate levels of risk. This is primarily a result of two factors. The first is in relation to the randomization process, as no study supplied enough information to determine whether the allocation sequence was concealed from participants and as such, we could not confidently code this domain was low risk. Additionally, all studies were determined to have “some concerns” in relation to the selection of reported results (domain 5) as no studies provided information regarding whether a pre‐specified analysis plan (e.g., trial protocol) was followed. As these studies were primarily government reports, it is unlikely there was a formal, approved scientific study protocol developed.

Regarding the non‐randomized designs, risk of bias was assessed across seven domains related to confounding, selection of participants, classification of interventions, deviation from intended interventions, missing outcome data, outcome measurement, and selection of the reported result. Six program sites received a judgment of moderate, six of serious, and two of critical. The main domain of concern was that of bias due to confounding. Due to the nature of the assessed interventions, control of potential confounding variables to a similar level as RCT designs is not feasible. Many of the interventions occur in the community, with a variety of potential influences that are out of the evaluators' scope of control. As such, all studies received a judgment of moderate or higher on that domain, contributing to the final overall judgments. Six program sites were scored as moderate for confounding due to evaluators attempts to control for some potential confounds, such as utilizing multivariate analyses (e.g., Abrams et al., 2008; Andersson Vogel et al., 2014; Bouffard & Bergseth, 2008; Liberman et al., 2021; Stafford & Glassner, 2012) or propensity score matching (e.g., Liberman et al., 2021). We note that these studies and methods were unable to account for all possible confounding factors. The remaining studies were judged as serious or critical risk for confounding due to minimal efforts to control for confounding.

**Figure 3:**
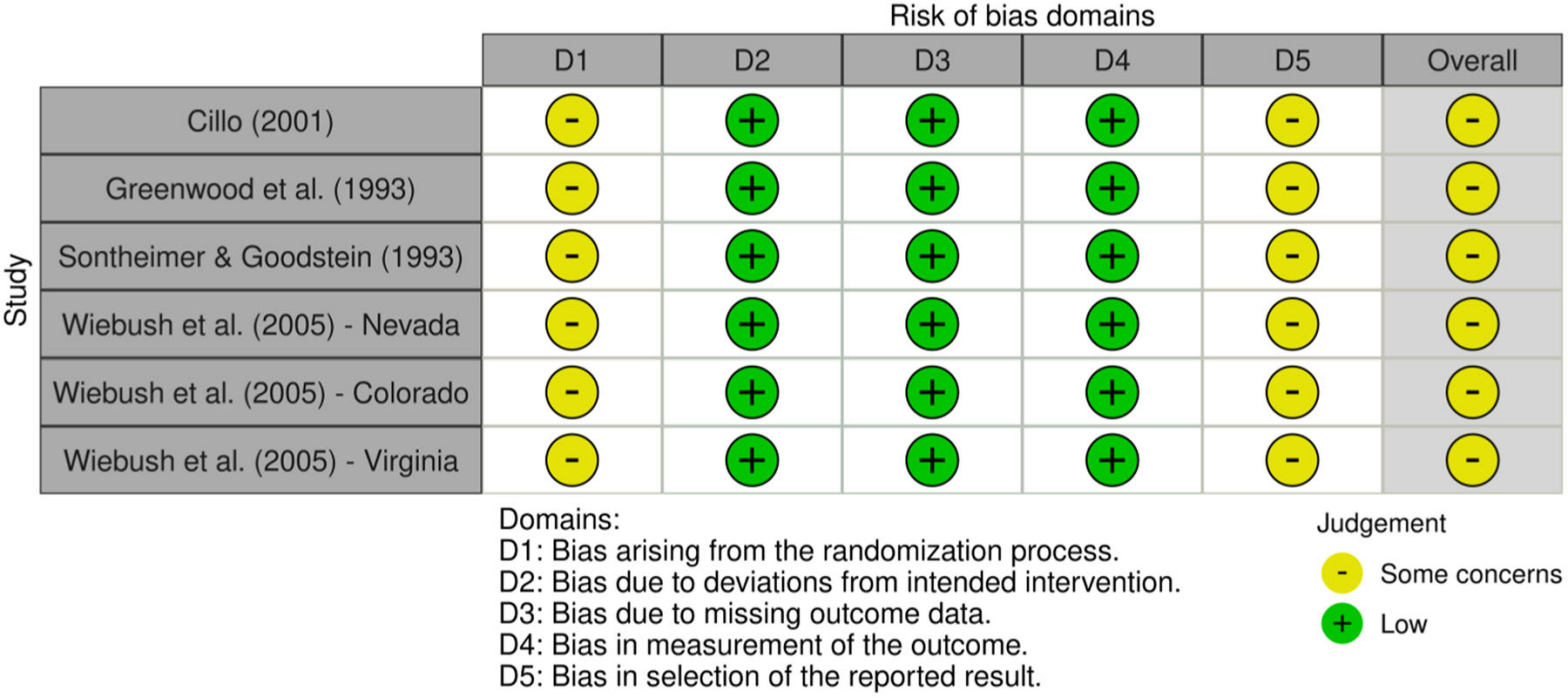
Risk of bias assessments for randomized design studies (RoB‐2).

**Figure 4:**
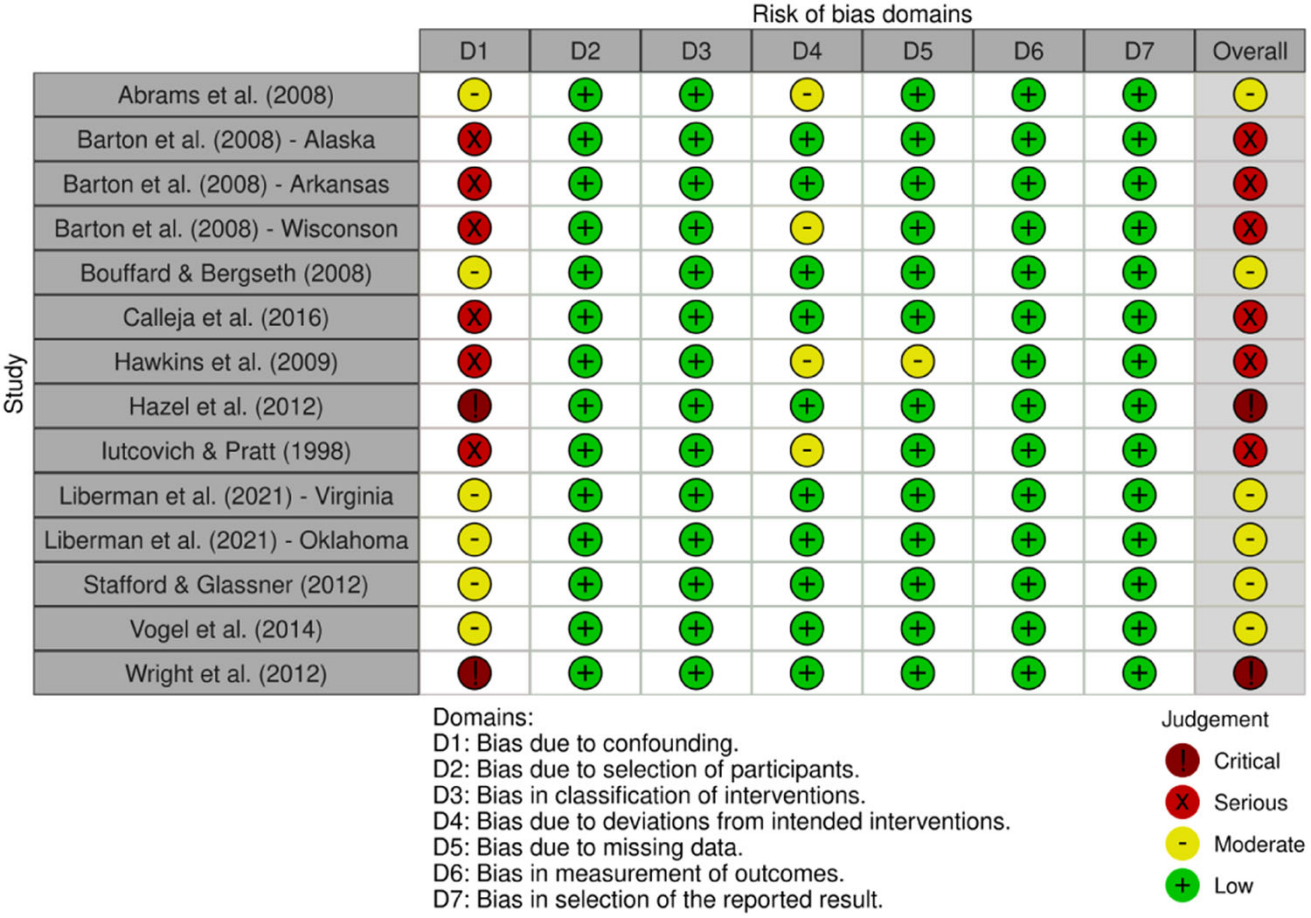
Risk of bias assessments for non‐randomized design studies (ROBINS‐I).

Based on these tools, the evaluations of 12 program sites were assessed as being low/moderate risk for bias, while 7 were deemed serious risk (see Figures 3 and 4, generated using robvis; McGuinness & Higgins, 2020).

##### Implementation studies

Qualitative implementation studies were assessed for quality and risk of bias using the Critical Appraisal Skills Program qualitative study checklist (CASP, 2018). The present sample included 19 publications^16^ that reported qualitative process evaluation data, representing 27 program sites. Of these, 12 were rated as high quality, and the remaining 7 were rated as moderate quality. No included studies were rated as low quality. See Table 10.

**Table 10:** CASP (2018) qualitative study quality appraisal checklist.^a^

	CASP (2018) checklist item										
Author(s), year – sample (if applicable)	Was there a clear statement of the aims of the research?	Is a qualitative methodology appropriate?	Was the research design appropriate to address the aims of the research?	Was the recruitment strategy appropriate to the aims of the research?	Was the data collected in a way that addressed the research issue?	Has the relationship between researcher and participant been adequately considered?	Have ethical issues been taken into consideration?	Was the data analysis sufficiently rigorous?	Is there a clear statement of findings?	How valuable is the research?	Overall quality of the study^b^
Abrams et al. (2008)	Y	Y	Y	Y	Y	C	Y	Y	Y	High value	H
Barton et al. (2008) – Alabama	Y	Y	Y	Y	Y	Y	Y	C	Y	High value	H
Barton et al. (2008) – Alaska	Y	Y	Y	Y	Y	Y	Y	C	Y	High value	H
Barton et al. (2008) – Arkansas	Y	Y	Y	Y	Y	Y	Y	C	Y	High value	H
Barton et al. (2008) – Wisconsin	Y	Y	Y	Y	Y	Y	Y	C	Y	High value	H
Citizens' Committee for Children of New York (2000)	Y	Y	C	C	Y	C	C	C	Y	High value	M
Dum and Fader (2013)	Y	Y	Y	C	Y	C	N	C	Y	High value	M
Flynn et al. (2003)	Y	Y	Y	Y	Y	Y	N	C	Y	High value	H
Hazel and Hampson (2015)	Y	Y	Y	Y	Y	Y	Y	Y	Y	High value	H
Hazel et al. (2008)	Y	Y	Y	Y	Y	Y	Y	Y	Y	High value	H
Hazel et al. (2012)	Y	Y	Y	Y	Y	C	C	C	Y	High value	M
Ipsos MORI (2012)	Y	Y	Y	Y	Y	C	Y	C	Y	High value	H
Iutcovich and Pratt (1998) – Philadelphia	Y	Y	Y	Y	Y	Y	Y	Y	Y	High value	H
Iutcovich and Pratt (1998) – Allegheny	Y	Y	Y	Y	Y	Y	Y	Y	Y	High value	H
Jain et al. (2018)	Y	Y	Y	Y	Y	C	C	Y	Y	High value	H
Liberman et al. (2021) – California	Y	Y	Y	Y	Y	C	Y	C	Y	High value	H
Liberman et al. (2021) – Texas	Y	Y	Y	Y	Y	C	Y	C	Y	High value	H
Liberman et al. (2021) – Virginia	Y	Y	Y	Y	Y	C	Y	C	Y	High value	H
Liberman et al. (2021) – Oklahoma	Y	Y	Y	Y	Y	C	Y	C	Y	High value	H
Lindquist et al. (2014) – Green Reentry	Y	Y	Y	Y	Y	Y	Y	Y	Y	High value	H
McKay et al. (2014) – Green Reentry	Y	Y	Y	Y	Y	N	C	C	Y	High value	M
Miller and MacGillivray (2002)	Y	Y	C	C	C	N	N	Y	Y	High value	M
Sinclair et al. (2021)	Y	Y	Y	Y	Y	C	Y	Y	Y	High value	H
Andersson Vogel et al. (2014)	Y	Y	Y	Y	Y	C	C	C	Y	High value	M
Wiebush et al. (2005) – Colorado	Y	Y	Y	Y	Y	Y	Y	C	Y	High value	H
Wiebush et al. (2005) – Nevada	Y	Y	Y	Y	Y	Y	Y	C	Y	High value	H
Wiebush et al. (2005) – Virginia	Y	Y	Y	Y	Y	Y	Y	C	Y	High value	H
Wright et al. (2012)	Y	Y	Y	Y	Y	C	C	C	Y	High value	M

^a^ Based on the guidelines set out in the CASP (2018) Qualitative Checklist, the following scores were used to grade studies on each item: *Y* = yes, *C* = Can't tell, *N* = No, *N*/*A* = not applicable.

^b^ The overall quality of a study was designated as *H* = high overall quality, *M* = moderate overall quality, and *L* = low overall quality. Decisions were based on cut‐off scores, modeled after Templeton et al. (2017), where studies with 8–10 “yes” scores were rated as high quality, 5–7 “yes” scores were rated as moderate quality, and 1–4 “yes” scores were rated as low quality.

##### Cost studies

As discussed in the protocol, methodological quality and risk of bias of studies presenting a cost analysis were intended to be assessed using the Consensus on Health Economic Criteria (CHEC‐list) developed by Evers et al. (2005). However, given the small sample of cost‐related studies (*k* = 7) identified and the lack of available cost data, we determined that this step was not useful, and the checklist was not implemented.

### 5.2 Quantitative synthesis of results

#### 5.2.1 Arrest outcome (*k* = 14)

**Figure 5:**
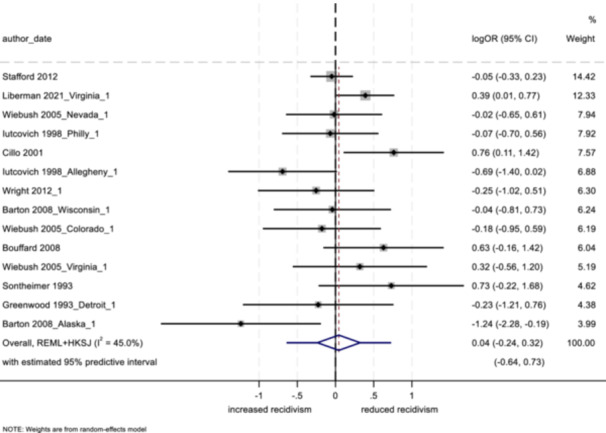
Forest plot for arrest outcome (k = 14).

The meta‐analysis for the set of 14 studies examining the outcome of arrest resulted in a small, positive, non‐statistically significant pooled LOR of 0.043 (95% CI [−0.236, 0.323], *t* = 0.335, *p* = 0.743). The 95% prediction interval ranges from −0.64 to 0.73 (see Figure 5), and the pooled set of studies included 1342 treatment group participants and 1509 comparison group participants. The set of 14 studies includes arrest outcomes measured at different time points post‐release; 12 months was the most common (8 studies), while one study measured outcomes at 1.5 months, two measured outcomes at 3 months, one at 6 months, and two at 18 months.

In odds ratio metrics, the pooled effect is 1.043, with a prediction interval of 0.527 to 2.075. This finding suggests that aftercare/resettlement programs do not have a meaningful impact on arrest rates for participants relative to comparison group subjects. Heterogeneity in the set of effect sizes was moderate to high, with tau^2^ = 0.081 and a significant *Q*‐statistic (*Q* = 24.04, *p* = 0.031, *I* ^*2*^ = 45.9%), suggesting notable between‐study variation.

##### Reporting bias – Arrest outcome

**Figure 6:**
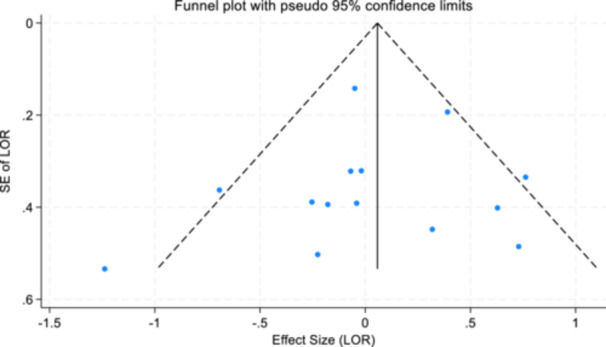
Funnel plot for arrest outcome (k = 14).

The funnel plot for the set of studies examining arrest was reasonably symmetric (Figure 6), with three studies outside the 95% confidence limits. Egger's test for small‐study effects was not significant (−0.379, *t* = −0.41, *p* = 0.687).

##### Sensitivity analysis – Arrest outcome

**Figure 7:**
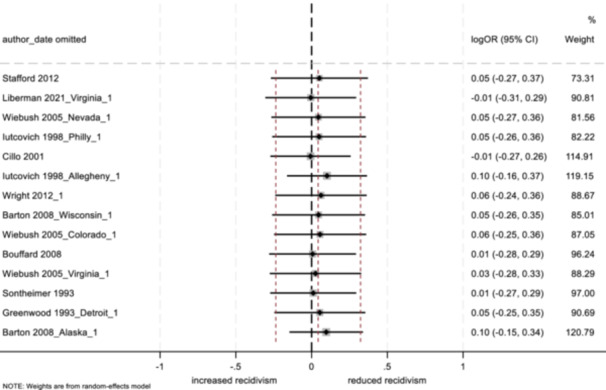
Influence analysis for arrest outcome (k = 14).

Figure 7 presents findings from the remove‐one‐study influence analysis. Results indicate that the non‐significant aggregate treatment effect is robust; the removal of any of the 14 effect sizes does not change the overall substantive finding.

Given potential differences in findings related to the time period of the outcome measure, sensitivity analyses were conducted by limiting study pools to those effect sizes measured at similar time points. Doing so allows for more homogenous analyses with respect to the follow‐up time periods examined in each study. Specifically, one analysis included the two studies which measured outcomes at 3 months post‐release. The pooled odds ratio was 1.215 and non‐statistically significant (*t* = 0.398, *p* = 0.759). Similarly, a pool of the two studies measuring outcomes at 18 months produced a non‐statistically significant odds ratio of 0.698 (*t* = −1.152, *p* = 0.455). Last, a pooled analysis limited to the eight studies measuring outcomes at 12 months was conducted; the resulting odds ratio was 1.00 (*t* = 0.003, *p* = 0.998). None of these analyses resulted in a substantive change to the original finding of no treatment impact on the outcome of arrest.

##### Moderator analysis – Arrest outcome

Thirteen potential moderators of aftercare/resettlement treatment impact on arrest outcomes were examined. As noted previously, only moderators with a minimum of 3 effect sizes per group were included; see Table 11 for an overview of all findings.

**Figure 8:**
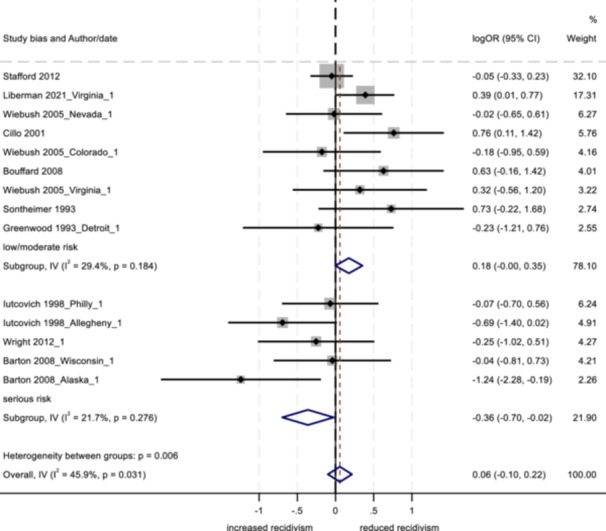
Forest plot for research bias moderator for arrest outcome (k = 14).

Two variables related to study design were included: bias score and treatment group sample size. The bias score rating was found to significantly moderate the impact of the aftercare/resettlement intervention (*Q*_*B*_ = 7.60, *p* < 0.01). Specifically, 9 of the 14 studies were rated as low/moderate risk (64%); these studies produced a positive, non‐statistically significant odds ratio of 1.191. For the 5 studies rated as serious risk (36%), the pooled effect was negative and statistically significant (OR = 0.698, *p* < 0.05). See Figure 8. These findings indicate that studies rated as having notable bias were related to negative treatment effects.

No other variables were found to be significant moderators of treatment impact, include two variables related to sample characteristics (sample ethnicity and sample gender), six program/intervention characteristics (whether the program included life skills training, housing, individual therapy, family therapy, mentoring, or substance use treatment), and three variables related to program delivery (whether the program was delivered by probation officers, program staff, or community service providers (not mutually exclusive)). See Table 11.

**Table 11:** Arrest outcome: Summary of moderator analyses (*k* = 14).

Type	Moderator	Definition	*N* (%) per group	LOR, *z*, *p*‐value, and heterogeneity measures
Study characteristics	Bias score	Low/medium	9 (64%)	LOR = 0.175, *z* = 1.926, *p* = 0.054
		Serious	5 (36%)	LOR = −0.360, *z* = −2.099, *p =* 0.036*
				*Q*_*T*_ = 24.04, *p* = 0.031*
				*Q*_*B*_ = 7.60, *p* = 0.006**
	Tx group sample size	<75	8 (57%)	LOR = 0.131, *z* = 0.915, *p* = 0.360
		75+	6 (43%)	LOR = 0.024, *z* = 0.249, *p =* 0.804
				*Q*_*T*_ = 24.04, *p* = 0.031*
				*Q*_*B*_ = 0.38, *p* = 0.537
Sample characteristics	Sample ethnicity	White/mixed	5 (36%)	LOR = −0.106, *z* = −0.605, *p* = 0.545
		Minority	9 (64%)	LOR = 0.101, *z* = 1.121, *p =* 0.262
				*Q*_*T*_ = 24.04, *p* = 0.031*
				*Q*_*B*_ = 1.11, *p* = 0.293
	Sample gender	Mixed gender	6 (43%)	LOR = 0.089, *z* = 0.898, *p* = 0.369
		All male	8 (57%)	LOR = −0.003, *z* = −0.020, *p =* 0.984
				*Q*_*T*_ = 24.04, *p* = 0.031*
				*Q*_*B*_ = 0.29, *p* = 0.591
Program components	Life skills^1^	No	5 (38%)	LOR = −0.083, *z* = −0.716, *p* = 0.474
		Yes	8 (62%)	LOR = 0.088, *z* = 0.643, *p =* 0.520
				*Q*_*T*_ = 20.43, *p* = 0.059
				*Q*_*B*_ = 0.91, *p* = 0.341
	Housing	No	8 (57%)	LOR = −0.052, *z* = −0.489, *p* = 0.625
		Yes	6 (43%)	LOR = 0.206, *z* = 1.673, *p =* 0.094
				*Q*_*T*_ = 24.04, *p* = 0.031*
				*Q*_*B*_ = 2.52, *p* = 0.112
	Individual therapy^1^	No	3 (23%)	LOR = −0.268, *z* = −1.189, *p* = 0.234
		Yes	10 (77%)	LOR = 0.035, *z* = 0.360, *p =* 0.719
				*Q*_*T*_ = 20.43, *p* = 0.059
				*Q*_*B*_ = 1.52, *p* = 0.217
	Family therapy	No	4 (29%)	LOR = 0.043, *z* = 0.349, *p* = 0.727
		Yes	10 (71%)	LOR = 0.069, *z* = 0.651, *p =* 0.515
				*Q*_*T*_ = 24.04, *p* = 0.031*
				*Q*_*B*_ = 0.03, *p* = 0.869
	Mentoring^1^	No	6 (46%)	LOR = 0.242, *z* = 1.498, *p* = 0.134
		Yes	7 (54%)	LOR = −0.120, *z* = −1.139, *p =* 0.255
				*Q*_*T*_ = 20.43, *p =* 0.059
				*Q*_*B*_ = 3.52, *p* = 0.060
	Substance use treatment^1^	No	4 (29%)	LOR = 0.098, *z* = 0.799, *p* = 0.424
		Yes	10 (71%)	LOR = 0.028, *z* = 0.258, *p* = 0.796
				*Q*_*T*_ = 24.04, *p* = 0.031*
				*Q*_*B*_ = 0.19, *p* = 0.665
Program delivery	Probation officers	No	8 (57%)	LOR = −0.016, *z* = −0.176, *p* = 0.860
		Yes	6 (43%)	LOR = 0.289, *z* = 1.773, *p* = 0.076
				*Q*_*T*_ = 24.04, *p* = 0.031*
				*Q*_*B*_ = 2.66, *p* = 0.103
	Program staff	No	5 (36%)	LOR = 0.127, *z* = 1.109, *p* = 0.267
		Yes	9 (64%)	LOR = −0.009, *z* = −0.078, *p* = 0.938
				*Q*_*T*_ = 24.04, *p* = 0.031*
				*Q*_*B*_ = 0.72, *p* = 0.397
	Community service providers	No	3 (21%)	LOR = 0.006, *z* = 0.046, *p* = 0.963
		Yes	11 (79%)	LOR = 0.091, *z* = 0.885, *p* = 0.376
				*Q*_*T*_ = 24.04, *p* = 0.031*
				*Q*_*B*_ = 0.27, *p* = 0.605

^1^ One study did not provide information.

* *p* < 0.05;

** *p* < 0.01.

To examine the joint impact of moderator variables, we implemented three random effects meta‐regression models (with the Knapp Hartung correction technique). The first model included the two study design characteristics and the two study sample characteristics, the second model incorporated the six program component characteristics, and the third model included the three program delivery characteristics. The model *F*‐statistics were all nonsignificant; indicating that, when looked at jointly, none of the variables were important moderators of aftercare/resettlement intervention impact on arrests.

In addition, to examine the impact of timing of outcome measure, we implemented a random effects meta‐regression (with the Knapp Hartung correction) to the full set of 17 studies with time to outcome measured in weeks. The model was implemented with time to outcome as the sole predictor, and in combination with the two study design characteristics and the two study sample characteristics. Both model *F*‐statistics were non‐significant, and none of the variables were important moderators of treatment effect on arrests.

#### 5.2.2 Conviction outcome (*k* = 13)

The pooled analysis for the outcome of conviction included 13 effect sizes based on 1357 treatment group and 1536 comparison group participants. The set of 13 studies included outcomes measured at different time points post‐release; 12 months was the most common (10 studies), while one study measured outcomes at 3 months, and two measured outcomes at 24 months.

**Figure 9:**
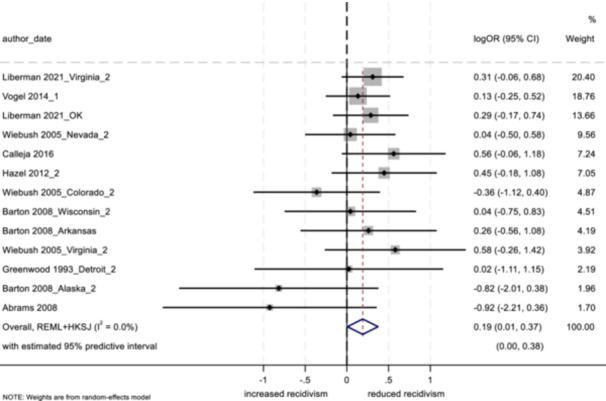
Forest plot for conviction outcome (k = 13).

The pooled result was a positive LOR of 0.190 (95% CI [0.006, 0.373], with a corresponding odds ratio of 1.209 and 95% prediction interval of 1.00 to 1.462. The effect was statistically significant (*t* = 2.256, *p* = 0.044), and heterogeneity was low (tau^2^ = 0.00, *Q* = 11.69, *I* ^*2*^ = 0%). See Figure 9.

##### Reporting bias – Conviction outcome

**Figure 10:**
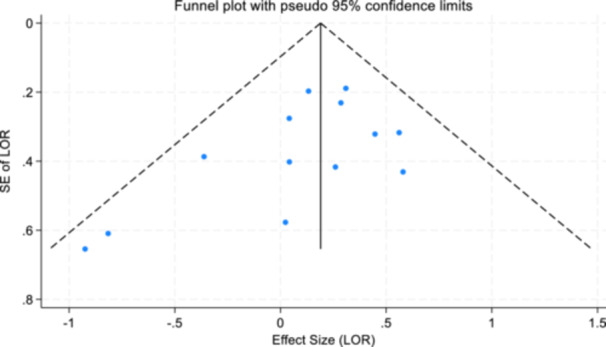
Funnel plot for conviction outcome (k = 13).

Figure 10 presents a funnel plot showing all studies falling within the 95% confidence intervals; Egger's test for small‐study effects was not significant (bias = −1.231, *t* = −1.81, *p* = 0.098).

##### Sensitivity analysis – Conviction outcome

**Figure 11:**
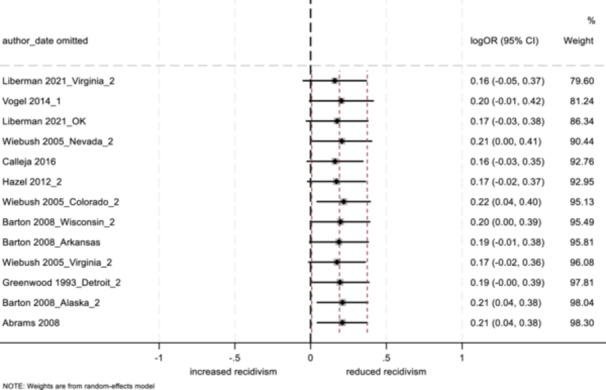
Influence analysis for conviction outcome (k = 13).

Results of the influence analysis indicate that the pooled finding is not robust; removing any of 8 of the 13 effect sizes is sufficient to reduce the aggregate LOR to a non‐statistically significant level favoring the treatment group. For example, removing the study by Liberman et al. (2021) ‐ Virginia would result in a pooled odds ratio of 1.172 (i.e., LOR = 0.159, 95%CI [−0.053, 0.372]). See Figure 11.

**Figure 12:**
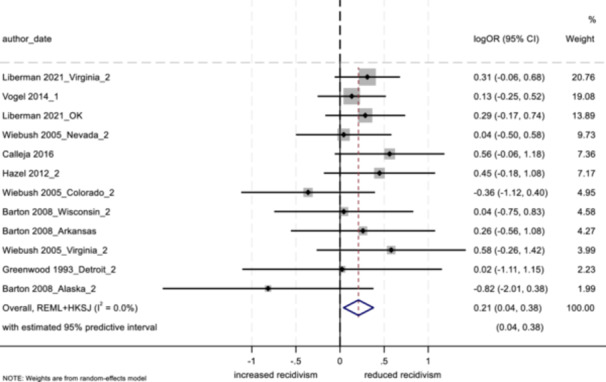
Forest plot for conviction outcome: Removing 1 study (k = 12).

Upon close examination, the aftercare intervention evaluated by Abrams et al. (2008) is arguably different than the other programs in the set, as it is the only intervention which takes place solely during a custodial phase.^17^ As a sensitivity test we removed this effect size from the set; the pooled LOR increased slightly to 0.209 (OR = 1.232), a statistically significant effect (*t* = 2.728, *p* = 0.020). The 95% prediction interval in odds ratios was 1.041 to 1.462; see Figure 12.

Given potential differences related to the time period in which outcomes were measured, additional sensitivity analyses were conducted by limiting study pools to effect sizes measured at similar time points. Specifically, the two studies which measured outcomes at 24 months post‐custodial release were pooled, resulting in a non‐statistically significant odds ratio of 1.317 (*t* = 1.363, *p* = 0.403). In addition, a pooled analysis using only the 10 studies measuring outcomes at 12 months was conducted; the resulting LOR of 0.139 (95%CI [−0.102, 0.379]) was not statistically significant, with a corresponding odds ratio of 1.149 (*t* = 1.305, *p* = 0.224).

Overall, results of the sensitivity testing suggest that the aggregate impact of aftercare/resettlement programs on conviction is marginal, and is acutely sensitive to the addition or removal of individual studies. Strong conclusions based on these findings are not warranted.

##### Moderator analysis – Conviction outcome

Twelve potential moderators of the impact of aftercare/resettlement interventions on conviction outcomes were examined. As noted previously, only moderators with a minimum of three effect sizes per group were included; see Table 12 for a summary of findings.

Four variables related to study design were included: peer‐reviewed status, research design, bias score, and treatment group sample size. While none of the variables were found to significantly moderate the impact of the aftercare/resettlement intervention (i.e., non‐significant between‐groups heterogeneity statistics), two of the subgroups produced significant effects on their own.

###### Experimental design

**Figure 13:**
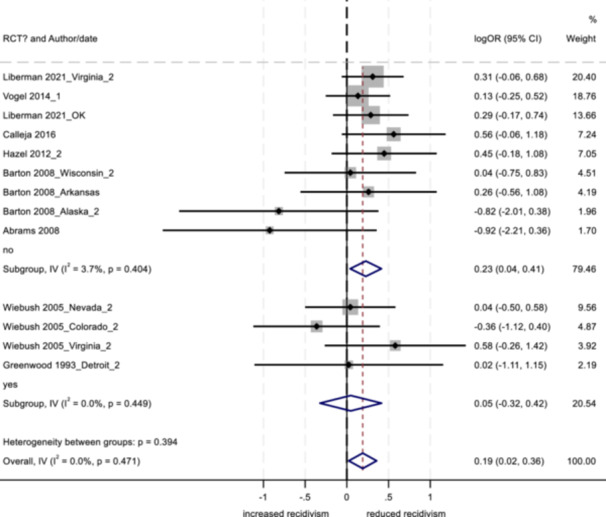
Forest plot for experimental design moderator for conviction outcome (k = 13).

A significant, positive subgroup effect on the outcome of conviction was found for the group of nine quasi‐experimental studies (OR = 1.254, *z* = 2.371, *p* = 0.018); see Figure 13.

###### Treatment group sample size

**Figure 14:**
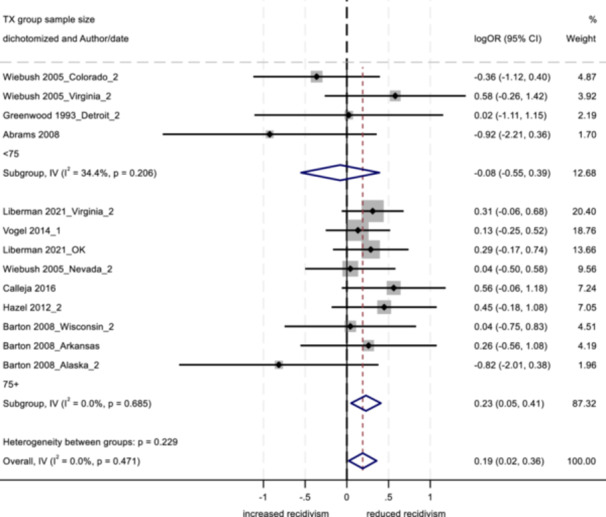
Forest plot for treatment group sample size moderator for conviction outcome (k = 13).

As shown in Figure 14, the aggregate impact of studies with samples of over 75 participants was positive and statistically significant (LOR = 0.229, *z* = 2.508, *p* = 0.012).

**Figure 15:**
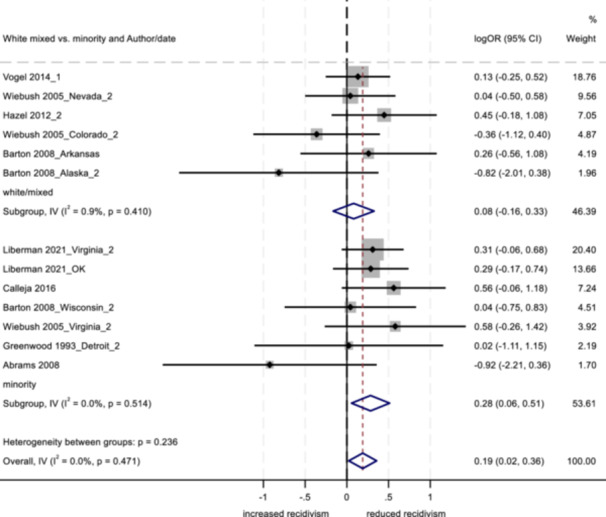
Forest plot for sample ethnicity moderator for conviction outcome (k = 13).

Two variables related to sample characteristics were included: sample ethnicity and sample gender. The between‐group heterogeneity statistic was non‐significant for both analyses, indicating that neither were significant moderators of treatment impact. However, samples that included predominantly minority group participants resulted in a positive and significant overall pooled effect (LOR = 0.284, *z* = 2.437, *p* = 0.015). See Figure 15.

###### Sample ethnicity

With respect to program components, no significant moderating effect was found for the moderators of housing, individual therapy, group therapy, or mentoring.

**Figure 16:**
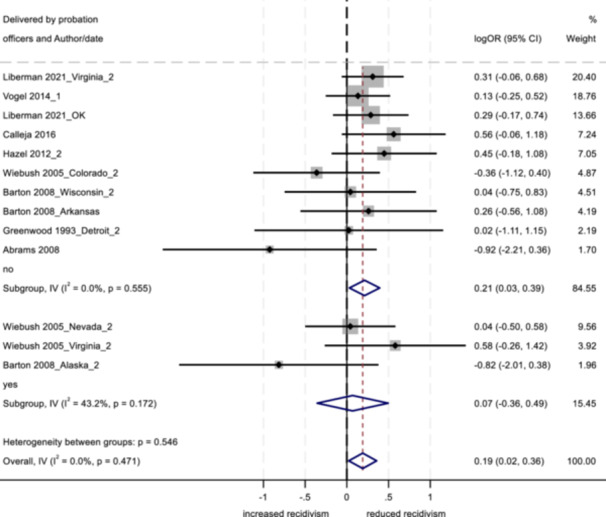
Forest plot for probation officer delivery moderator for conviction outcome.

Last, program delivery by probation officers and community service providers was examined. While no significant between‐group heterogeneity was identified, the group of 10 studies involving a program which did *not* include probation officers resulted in a positive, statistically significant (LOR = 0.212, *z* = 2.284, *p* = 0.022). See Figure 16.

###### Delivery by probation officers

**Figure 17:**
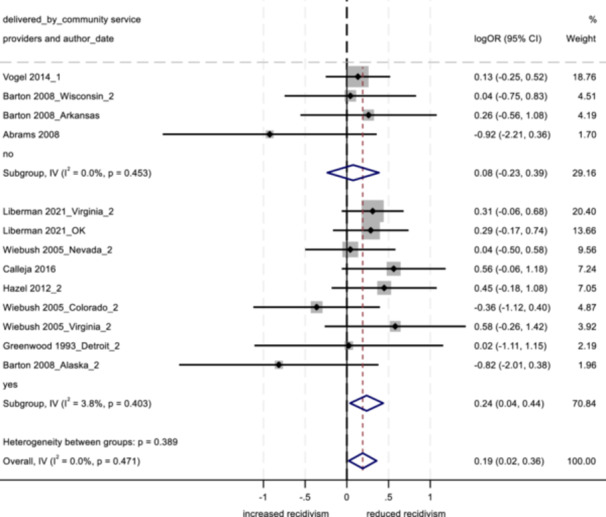
Forest plot for community service provider delivery moderator for conviction outcome.

In addition, the subgroup of nine studies which involved community service providers produced a positive pooled LOR of 0.237, *z* = 2.339, *p* = 0.019. See Figure 17 and Table 12.

**Table 12:** Conviction outcome: Summary of moderator analyses (*k* = 13).

Type	Moderator	Definition	*N* (%) per group	LOR, *z*, *p*‐value, and heterogeneity measures
Study characteristics	Peer‐review	No	10 (77%)	LOR = 0.194, *z* = 1.931, *p* = 0.053
		Yes	3 (23%)	LOR = 0.180, *z* = 1.109, *p* = 0.267
				*Q*_*T*_ = 11.69, *p* = 0.471
				*Q*_*B*_ = 0.01, *p* = 0.942
	Research design	Non‐RCT	9 (69%)	LOR = 0.227, *z* = 2.371, *p* = 0.018*
		RCT	4 (31%)	LOR = 0.047, *z* = 0.249, *p* = 0.804
				*Q*_*T*_ = 11.69, *p* = 0.471
				*Q*_*B*_ = 0.73, *p* = 0.394
	Bias score	Low/medium	8 (62%)	LOR = 0.161, *z* = 1.637, *p* = 0.102
		Serious	5 (38%)	LOR = 0.276, *z* = 1.618, *p* = 0.106
				*Q*_*T*_ = 11.69, *p* = 0.471
				*Q*_*B*_ = 0.34, *p* = 0.559
	Tx group sample size	<75	4 (42%)	LOR = −0.080, *z* = −0.332, *p* = 0.740
		75+	9 (69%)	LOR = 0.229, *z* = 2.508, *p* = 0.012*
				*Q*_*T*_ = 11.69, *p* = 0.471
				*Q*_*B*_ = 1.45, *p* = 0.229
Sample characteristics	Sample ethnicity	White/mixed	6 (46%)	LOR = 0.081, *z* = 0.649, *p* = 0.517
		Minority	7 (54%)	LOR = 0.284, *z* = 2.437, *p =* 0.015*
				*Q*_*T*_ = 11.69, *p* = 0.471
				*Q*_*B*_ = 1.40, *p* = 0.236
	Sample gender	All male	4 (31%)	LOR = 0.183, *z* = 1.444, *p* = 0.149
		Mixed gender	9 (69%)	LOR = 0.196, *z* = 1.695, *p* = 0.090
				*Q*_*T*_ = 11.69, *p* = 0.471
				*Q*_*B*_ = 0.01, *p* = 0.941
Program components	Housing^1^	No	8 (67%)	LOR = 0.122, *z* = 0.889, *p* = 0.374
		Yes	4 (33%)	LOR = 0.217, *z* = 1.758, *p* = 0.079
				*Q*_*T*_ = 11.48, *p* = 0.404
				*Q*_*B*_ = 0.26, *p* = 0.610
	Individual therapy^2^	No	4 (36%)	LOR = −0.153, *z* = −0.631, *p* = 0.528
		Yes	7 (64%)	LOR = 0.199, *z* = 1.708, *p* = 0.088
				*Q*_*T*_ = 10.82, *p* = 0.372
				*Q*_*B*_ = 1.71, *p* = 0.191
	Group therapy^3^	No	7 (70%)	LOR = 0.186, *z* = 1.173, *p* = 0.241
		Yes	3 (30%)	LOR = 0.050, *z* = 0.249, *p* = 0.803
				*Q*_*T*_ = 10.82, *p* = 0.288
				*Q*_*B*_ = 0.29, *p* = 0.591
	Mentoring^3^	No	6 (60%)	LOR = 0.117, *z* = 0.745, *p* = 0.456
		Yes	4 (40%)	LOR = 0.160, *z* = 0.789, *p* = 0.430
				*Q*_*T*_ = 10.82, *p* = 0.288
				*Q*_*B*_ = 0.03, *p* = 0.867
Program delivery	Probation officers	No	10 (77%)	LOR = 0.212, *z* = 2.284, *p* = 0.022*
		Yes	3 (23%)	LOR = 0.069, *z* = 0.319, *p* = 0.749
				*Q*_*T*_ = 11.69, *p* = 0.471
				*Q*_*B*_ = 0.36, *p* = 0.546
	Community service providers	No	4 (29%)	LOR = 0.075, *z* = 0.477, *p* = 0.633
		Yes	9 (64%)	LOR = 0.237, *z* = 2.339, *p* = 0.019*
				*Q*_*T*_ = 11.69, *p* = 0.471
				*Q*_*B*_ = 0.74, *p* = 0.389

^1^ One study did not provide information.

^2^ Two studies not did provide information.

^3^ Three studies did not provide information.

* *p* < 0.05.

###### Delivery by community service providers

To examine the joint impact of moderators, three random effects meta‐regression models were implemented (with the Knapp Hartung correction technique). Using a similar approach as for the arrest outcome, the first model included the four study characteristics, the second model included the two study sample characteristics and the two program delivery characteristics, and the third model incorporated the four program component characteristics. No significant model statistics were noted, again suggesting that none of the variables are important moderators of aftercare/resettlement intervention impact on conviction outcomes.

To examine the impact of timing of outcome measure, we implemented a random effects meta‐regression (with the Knapp Hartung correction) to the full set of 18 studies with time to outcome measured in weeks. The model was implemented with time to outcome as the sole predictor, and in combination with the four study design characteristics. Both model *F*‐statistics were non‐significant, and none of the variables were important moderators of treatment effect on conviction.

#### 5.2.3 Incarceration outcome (*k* = 8)

**Figure 18:**
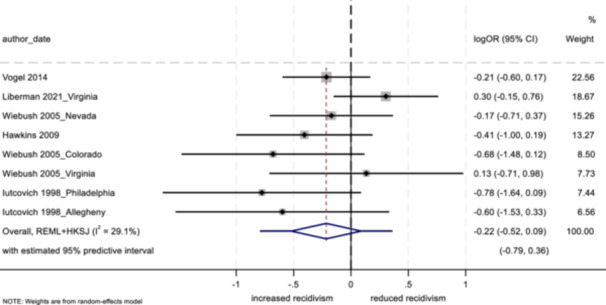
Forest plot for incarceration outcome (k = 8).

Last, we pooled outcomes from eight studies examining the effect of aftercare/resettlement interventions on incarceration; these studies included 848 treatment group participants and 1087 comparison group participants. Of the eight studies, six measured outcomes at 6 months post‐custodial release, one measured outcomes 15 months post‐release, and one study measured outcomes at 24 months post‐release. Heterogeneity was low across the set of studies (tau^2^ = 0.039, *Q* = 9.56, *p* = 0.215, *I* ^*2*^ = 26.8%). As shown in Figure 18, results were not statistically significant. The pooled odds ratio was 0.806 (LOR = −0.216, *t* = −1.674), with a 95% prediction interval of OR = 2.203 to OR = 1.433.

##### Reporting bias – Incarceration outcome

**Figure 19:**
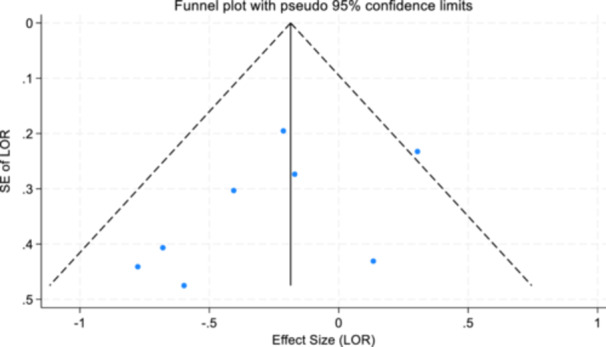
Funnel plot for incarceration outcome (k = 8).

The funnel plot shows some degree of asymmetry, with studies clustered in the lower left quadrant. Egger's test was not significant (bias = −1.832, *t* = −1.47, *p* = 0.191). See Figure 19.

##### Sensitivity analysis – Incarceration outcome

**Figure 20:**
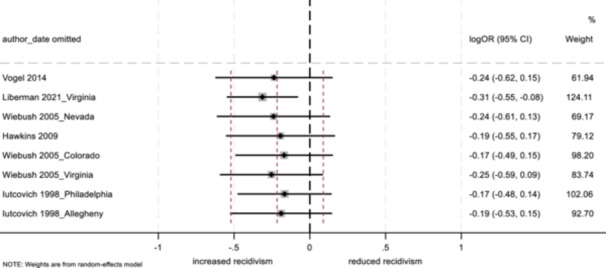
Influence analysis for incarceration outcome (k = 8).

The influence analysis involving removal of each study one at a time did not produce any substantively different findings; in other words, the aggregate (lack of) effect on incarceration outcomes is negative and robust and no study exerts a disproportionate effect. See Figure 20.

As an additional sensitivity analyses, the two studies measuring outcomes at 15 and 24 months were dropped from the set, leaving only the six studies using the commensurate 12 month outcome period. No substantive difference in findings emerged; the pooled odds ratio was negative and non‐statistically significant at 0.807 (*t* = −1.144, *p* = 0.304).

##### Moderator analysis – Incarceration outcome

**Figure 21:**
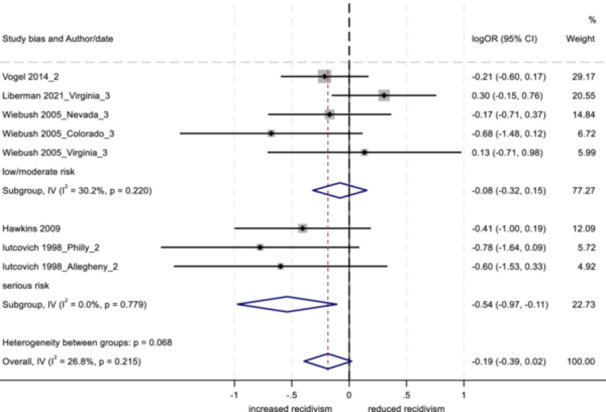
Forest plot for bias score moderator for incarceration outcome (k = 8).

Despite the lack of evidence of heterogeneity in the pooled set of studies, six variables were examined as potential moderators for the incarceration outcome; see Table 13 for an overview of findings. No significant between‐group heterogeneity statistics were observed; two subgroups produced notable pooled effects. First, in terms of the bias score rating, studies rated as having serious bias were related to a statistically significant negative pooled odds ratio of 1.718 (LOR = −0.541, *z* = −2.445, *p* < 0.05). In other words, studies rated as high bias had a negative effect on incarceration; see Figure 21.

###### Bias rating

###### Gender

**Figure 22:**
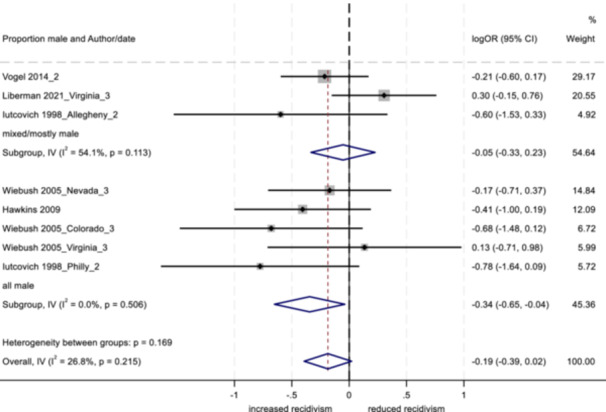
Forest plot for gender moderator for incarceration outcome (k = 8).

Second, with respect to gender, interventions serving samples of all male participants were related to statistically significant, negative effects on the outcome of incarceration (LOR = −0.345, *z* = −2.203, *p* < 0.05). See Figure 22 and Table 13.

**Table 13:** Incarceration outcome: Summary of moderator analyses (*k* = 8).

Type	Moderator	Definition	*N* (%) per group	LOR, *z*, *p*‐value, and heterogeneity measures
Study characteristics	Research design	Non‐RCT	5 (63%)	LOR = −0.170, *z* = −1.369, *p* = 0.171
		RCT	3 (38%)	LOR = −0.229, *z* = −1.138, *p* = 0.255
				*Q*_*T*_ = 9.56, *p* = 0.215
				*Q*_*B*_ = 0.06, *p* = 0.803
	Bias score	Low/medium	5 (63%)	LOR = −0.081, *z* = −0.679, *p* = 0.497
		Serious	3 (38%)	LOR = −0.541, *z* = −2.445, *p* = 0.014*
				*Q*_*T*_ = 9.56, *p* = 0.215
				*Q*_*B*_ = 3.33, *p* = 0.068
	Tx group sample size	<75	3 (38%)	LOR = −0.381, *z* = −1.516, *p* = 0.129
		75+	5 (63%)	LOR = −0.144, *z* = −1.240, *p* = 0.215
				*Q*_*T*_ = 9.56, *p* = 0.215
				*Q*_*B*_ = 0.73, *p* = 0.392
Sample characteristics	Sample ethnicity	White/mixed	3 (38%)	LOR = −0.263, *z* = −1.776, *p* = 0.076
		Minority	5 (63%)	LOR = −0.106, *z* = −0.708, *p =* 0.479
				*Q*_*T*_ = 9.56, *p* = 0.215
				*Q*_*B*_ = 0.55, *p* = 0.458
	Sample gender	Mixed gender	3 (38%)	LOR = −0.054, *z* = −0.377, *p* = 0.706
		All male	5 (63%)	LOR = −0.345, *z* = −2.203, *p* = 0.028*
				*Q*_*T*_ = 9.56, *p* = 0.215
				*Q*_*B*_ = 1.89, *p* = 0.169
Program components	Housing	No	3 (38%)	LOR = −0.229, *z* = −1.138, *p* = 0.255
		Yes	5 (63%)	LOR = −0.170, *z* = −1.369, *p* = 0.171
				*Q*_*T*_ = 9.56, *p* = 0.215
				*Q*_*B*_ = 0.06, *p* = 0.803

* *p* < 0.05.

To examine the joint impact of moderators, two random effects meta‐regression models were implemented (with the Knapp Hartung correction technique). The first model included the three study characteristics, and the second model included the two study sample characteristics and the one program component characteristic. Again, no significant model statistics were noted; none of the variables examined are important moderators of aftercare/resettlement intervention impact on the outcome incarceration.

To examine the impact of timing of outcome measure, we implemented a random effects meta‐regression (with the Knapp Hartung correction) to the full set of 8 studies with time to outcome measured in weeks. The model was implemented with time to outcome as the sole predictor, and in combination with the three study design characteristics. Both model *F*‐statistics were non‐significant, and none of the variables were important moderators of treatment effect on incarceration.

Tables 14 and 15 present summaries of the findings from the main analyses and the moderator analyses.

**Table 14:** Summary of main analysis findings.

Outcome category	No. of effect sizes	Pooled OR (LOR)	95% prediction interval (OR)	*t* (*p*‐value)	tau^2^, *Q*‐statistic (*p‐*value) *I* ^2^ statistic
Arrest	14	OR = 1.044	[0.527, 2.075]	0.335	tau^2^ = 0.081,
					*Q* = 24.04
		(LOR = 0.043)		(*p* = 0.743)	(*p* = 0.031*)
					*I* ^2^ = 45.9%
Conviction	13	OR =3.287	[1.000, 1.462]	2.256	tau^2^ = 0.000,
					*Q* = 11.69
		(LOR = 1.190)		(*p* = 0.044*)	(*p* = 0.471)
					*I* ^2^ = 0%
Incarceration	8	OR =0.806	[2.203, 1.433]	−1.674	tau^2^ = 0.039,
					*Q* = 9.56
		(LOR = −0.216)		(*p* = 0.138)	(*p* = 0.215).
					*I* ^2^ = 26.8%

**Table 15:** Summary of moderator analyses.

Moderator	Arrest	Conviction	Incarceration
*Study characteristics*			
Peer review	n/a	*No peer review*: LOR = 0.194, *z* = 1.931, *p* = 0.053 *Peer reviewed*: LOR = 0.180, *z* = 1.109, *p* = 0.267 *Q*_*B*_ = 0.01, *p* = 0.942	n/a
Research design	n/a	*Non‐RCT*: LOR = 0.227, *z* = 2.371, *p* = 0.018* *RCT*: LOR = 0.047, *z* = 0.249, *p* = 0.804 *Q*_*B*_ = 0.73, *p* = 0.394	*Non‐RCT*: LOR = −0.170, *z* = −1.369, *p* = 0.171 *RCT*: LOR = −0.229, *z* = −1.138, *p* = 0.255 *Q*_*B*_ = 0.06, *p* = 0.803
Bias score	*Low bias*: LOR = 0.175, *z* = 1.926, *p* = 0.054 *Serious bias*: LOR = −0.360, *z* = −2.099, *p =* 0.036* *Q*_*B*_ = 7.60, *p* = 0.006**	*Low bias*: LOR = 0.161, *z* = 1.637, *p* = 0.102 *Serious bias*: LOR = 0.276, *z* = 1.618, *p* = 0.106 *Q*_*B*_ = 0.34, *p* = 0.559	*Low bias*: LOR = −0.081, *z* = −0.679, *p* = 0.497 *Serious bias*: LOR = −0.541, *z* = −2.445, *p* = 0.014* *Q*_*B*_ = 3.33, *p* = 0.068
Tx group sample size	*<75*: LOR = 0.131, *z* = 0.915, *p* = 0.360 *75+*: LOR = 0.024, *z* = 0.249, *p =* 0.804 *Q*_*B*_ = 0.38, *p* = 0.537	*<75*: LOR = −0.080, *z* = −0.332, *p* = 0.740 *75+*: LOR = 0.229, *z* = 2.508, *p* = 0.012* *Q*_*B*_ = 1.45, *p* = 0.229	*<75*: LOR = −0.381, *z* = −1.516, *p* = 0.129 *75+*: LOR = −0.144, *z* = −1.240, *p* = 0.215 *Q*_*B*_ = 0.73, *p* = 0.392
*Sample characteristics*			
Sample ethnicity	*White/mixed*: LOR = −0.106, *z* = −0.605, *p* = 0.545 *Minority*: LOR = 0.101, *z* = 1.121, *p =* 0.262 *Q*_*B*_ = 1.11, *p* = 0.293	*White/mixed*: LOR = 0.081, *z* = 0.649, *p* = 0.517 *Minority*: LOR = 0.284, *z* = 2.437, *p =* 0.015* *Q*_*B*_ = 1.40, *p* = 0.236	*White/mixed*: LOR = −0.263, *z* = −1.776, *p* = 0.076 *Minority*: LOR = −0.106, *z* = −0.708, *p =* 0.479 *Q*_*B*_ = 0.55, *p* = 0.458
Sample gender	*Mixed gender: *LOR = 0.089, *z* = 0.898, *p* = 0.369 *All male*: LOR = −0.003, *z* = −0.020, *p =* 0.984 *Q*_*B*_ = 0.29, *p* = 0.591	*Mixed gender*: LOR = 0.183, *z* = 1.444, *p* = 0.149 *All male*: LOR = 0.196, *z* = 1.695, *p* = 0.090 *Q*_*B*_ = 0.01, *p* = 0.941	*Mixed gender*: LOR = −0.054, *z* = −0.377, *p* = 0.706 *All male*: LOR = −0.345, *z* = −2.203, *p* = 0.028* *Q*_*B*_ = 1.89, *p* = 0.169
*Program components*			
Life skills	*No*: LOR = −0.083, *z* = −0.716, *p* = .474 *Yes*: LOR = 0.088, *z* = 0.643, *p =* 0.520 *Q*_*B*_ = 0.91, *p* = 0.341	n/a	n/a
Housing	*No*: LOR = −0.052, *z* = −0.489, *p* = 0.625 *Yes*: LOR = 0.206, *z* = 1.673, *p* = 0.094 *Q*_*B*_ = 2.52, *p* = 0.112	*No*: LOR = 0.122, *z* = 0.889, *p* = 0.374 *Yes*: LOR = 0.217, *z* = 1.758, *p* = 0.079 *Q*_*B*_ = 0.26, *p* = 0.610	*No*: LOR = −0.229, *z* = −1.138, *p* = 0.255 *Yes*: LOR = −0.170, *z* = −1.369, *p* = 0.171 *Q*_*B*_ = 0.06, *p* = 0.803
Individual therapy	*No*: LOR = −0.268, *z* = −1.189, *p* = 0.234 *Yes*: LOR = 0.035, *z* = 0.360, *p* = 0.719 *Q*_*B*_ = 1.52, *p* = 0.217	*No*: LOR = −0.153, *z* = −0.631, *p* = 0.528 *Yes*: LOR = 0.199, *z* = 1.708, *p* = 0.088 *Q*_*B*_ = 1.71, *p* = 0.191	n/a
Group therapy	n/a	*No*: LOR = 0.186, *z* = 1.173, *p* = 0.241 *Yes*: LOR = 0.050, *z* = 0.249, *p* = 0.803 *Q*_*B*_ = 0.29, *p* = 0.591	n/a
Family therapy	*No*: LOR = 0.043, *z* = 0.349, *p* = 0.727 *Yes*: LOR = 0.069, *z* = 0.651, *p* = 0.515 *Q*_*B*_ = 0.03, *p* = 0.869	n/a	n/a
Mentoring	*No*: LOR = 0.242, *z* = 1.498, *p* = 0.134 *Yes*: LOR = −0.120, *z* = −1.139, *p =* 0.255 *Q*_*B*_ = 3.52, *p* = 0.060	*No*: LOR = 0.117, *z* = 0.745, *p* = 0.456 *Yes*: LOR = 0.160, *z* = 0.789, *p* = 0.430 *Q*_*B*_ = 0.03, *p* = 0.867	n/a
Subs use treat	*No*: LOR = 0.098, *z* = 0.799, *p* = 0.424 LOR = 0.028, *z* = 0.258, *p* = 0.796 *Q*_*B*_ = 0.19, *p* = 0.665	n/a	n/a
*Program delivery*			
Probation officers	*No*: LOR = −0.016, *z* = −0.176, *p* = 0.860 *Yes*: LOR = 0.289, *z* = 1.773, *p* = 0.076 *Q*_*B*_ = 2.66, *p* = 0.103	*No*: LOR = 0.212, *z* = 2.284, *p* = 0.022* *Yes*: LOR = 0.069, *z* = 0.319, *p* = 0.749 *Q*_*B*_ = 0.36, *p* = 0.546	n/a
Program staff	*No*: LOR = 0.127, *z* = 1.109, *p* = 0.267 *Yes*: LOR = −0.009, *z* = −0.078, *p* = 0.938 *Q*_*B*_ = 0.72, *p* = 0.397	n/a	n/a
Community service providers	*No*: LOR = 0.006, *z* = 0.046, *p* = 0.963 *Yes*: LOR = 0.091, *z* = 0.885, *p* = 0.376 *Q*_*B*_ = 0.27, *p* = 0.605	*No*: LOR = 0.075, *z* = 0.477, *p* = 0.633 *Yes*: LOR = 0.237, *z* = 2.339, *p* = 0.019* *Q*_*B*_ = 0.74, *p* = 0.389	n/a

* *p* < 0.05;

** *p* < 0.01.

### 5.3 Summary of findings from qualitative evidence

Based on the process analysis data found in the implementation studies, 15 themes were uncovered which represent common challenges and successes within aftercare/resettlement programming. First, themes which are not stage‐specific (i.e., are not only relevant to the pre‐release, intensive transition, or post‐release stages) are presented, wherein challenges, and then successes, are presented within each theme. Subsequently, we present themes specific to the pre‐release, intensive transition, and post‐release stages of aftercare/resettlement.

#### 5.3.1 Overall findings

As noted above, a number of themes emerged which were not specific to one of the three stages of aftercare/resettlement. Below, eight themes relevant to aftercare as a whole are presented within five domains: funding and program continuance; staffing and organization‐level findings; program fidelity; casework feasibility; and parents and families.

##### Funding and program continuance

*Theme 1: A lack of long‐term funding often results in program termination or poor service delivery*.

Several studies noted that programs had been terminated during the study period, in some cases due to funding issues (Barton et al., 2008; Liberman et al., 2021). In multiple program sites, a lack of leadership and difficulties operating the program past a term of grant funding led to program termination. For example, in its Alabama site, the Targeted Re‐Entry (TR) program suffered from a lack of administrative commitment and a weakened community network, which led to the decision to “…discontinue TR programming when the funding from the national [Boys and Girls Clubs of America] office was due to expire in December, 2006” (Barton et al., 2008, p. 26). Similarly, a reentry program implemented in Texas was terminated due to a lack of funding after Second Chance Act grant funding expired, while services in California and Oklahoma sites were reduced and enrollment waned (Liberman et al., 2021). Though it is not clear if the Green Reentry program is still running, Lindquist et al. (2014) also noted that “…at the conclusion of grant period, it was unclear how the overall planning and coordination of green activities and the oversight of specific green projects would be accomplished” (pp. 6–23). Though not all programs experience funding issues, programs seem to experience instability and challenges towards program fidelity due to a lack of funding, perhaps hampering the effectiveness of such programs.

Where funding was noted as a program success, improvements in service delivery were described. For example, additional funds provided to an Arkansas program resulted in “…the addition of two case managers to the [Arkansas Division of Youth Services] staff, a reduction in the role of the current aftercare provider, ongoing training in case management (to include the [Targeted Reentry] staff), and the development of a mentoring program in partnership with a local university” (Barton et al., 2008, p. 41). Similarly, Iutcovich and Pratt (1998) found that in Philadelphia, an adequate budget allowed smooth operation of the Pennsylvania Intensive Aftercare Program, while Miller and MacGillivray (2002) reported that service expansions were made possible by combining funding sources. Last, in its Virginia site, a Second Chance Act reentry program was able to renew its funding and “…funding for the program [continued to be] uninterrupted” throughout the study period (Liberman et al., 2021).

##### Staffing and organization‐level findings

*Theme 2: Aftercare/resettlement programs experience high levels of staff turnover due to a lack of attractive job features*.

Perhaps the most commonly noted challenge to program delivery and functioning was a high level of staff turnover (Barton et al., 2008; Dum & Fader, 2013; Flynn et al., 2003; Hazel et al., 2008; Hazel et al., 2012; Iutcovich & Pratt, 1998; Jain et al., 2018; Lindquist et al., 2014; Wiebush et al., 2005). While aftercare/resettlement programs may experience high staff turnover for a number of reasons, poor compensation, working conditions, staff demoralization, health and retirement benefits, qualification levels, and burnout were all noted as contributing factors (Barton et al., 2008; Dum & Fader, 2013; Flynn et al., 2003; Iutcovich & Pratt, 1998; Lindquist et al., 2014; Miller & MacGillivray, 2002). For example, in the Arkansas implementation of the Targeted Re‐Entry program, “issues of insufficient training and the lack of clinical supervision for the staff would come up again and again over the course of the project and [were] never completely resolved within the organization itself” (Barton et al., 2008, p. 41). In the Pennsylvania Intensive Aftercare programs, “managers experienced challenges in finding and retaining qualified, experienced staff, and cited the nature of the work and compensation issues as contributing to staff turnover and ‘burnout’” (Iutcovich & Pratt, 1998, p. 12).

*Theme 3: Poor leadership and organizational functioning often compromise aftercare/resettlement program delivery*.

A lack of leadership, administrative changes, and poor supervision were cited in some studies (Barton et al., 2008; Flynn et al., 2003; Liberman et al., 2021). For example, in the Wisconsin implementation of the Targeted Re‐Entry program, there were difficulties establishing an office which would supervise aftercare/resettlement staff, new management was not suited to supervising the program, and “the [Targeted Re‐Entry] project did not always receive the kind of attention it needed” (Barton et al., 2008, p. 46). Poor relationships between staff and leadership were also noted. In the Network Aftercare System, Flynn et al. found that “some of the staff seemed to feel that while they could communicate with those below them, upper management were not listening to their needs or being supportive of them … comments about opinions or issues “not being taken seriously” by supervisors were raised” (2003, p. 130).

A number of studies cited issues regarding role definition and conflicting authority. Staff and partners were often unsure of their responsibilities or the responsibilities of others, leading to inefficiencies and time‐wasting (Flynn et al., 2003; Hazel & Hampson, 2015; Hazel et al., 2008; Ipsos MORI, 2012; Andersson Vogel et al., 2014; Wright et al., 2012). For instance, in the Leaving Care Project, “when the coordinators started their work, no specific job description existed, but was formulated along the way” (Andersson Vogel et al., 2014, p. 250). In other cases, staff felt that they lacked the authority or power to properly do their jobs (Dum & Fader, 2013; Flynn et al., 2003; Andersson Vogel et al., 2014). For example, aftercare/resettlement workers in the Powelton Aftercare program “felt powerless because they lacked any ability to impose meaningful consequences upon their clients” (Dum & Fader, 2013, p. 794), indicating a lack of efficiency in implementing the program as intended and likely impacting the extent to which the program is adequately affecting participants.

*Theme 4: Staff and leadership often show high levels of care and dedication in their work*.

Some successes noted regarding staff included a high level of care towards youth, relevant and extensive prior experience, and dedication to the program (Barton et al., 2008; Dum & Fader, 2013; Miller & MacGillivray, 2002; Wiebush et al., 2005). Successful cooperation and communication were also common themes throughout studies. Good teamwork and interagency cooperation were cited as program successes, often exemplified by frequent meetings, strong communication, established community partnerships, and continuity of care across jurisdictions or program phases (Flynn et al., 2003; Ipsos MORI, 2012; Liberman et al., 2021; Lindquist et al., 2014; Wiebush et al., 2005). Regarding program leadership, strong management and administrative support, consistent leadership, and proper program oversight were found in some studies (Barton et al., 2008; Ipsos MORI, 2012; Lindquist et al., 2014; Wiebush et al., 2005).

##### Program fidelity

*Theme 5: Program fidelity is often hampered by weak or poorly communicated program theory, aims, and operation*.

Interventions seemed to suffer from a lack of program fidelity in many cases. For example, programs operated under various theoretical bases by different staff (e.g., treatment vs. punishment‐based), provided inconsistent or irrelevant case planning, emphasized some stages of the aftercare/resettlement process more than others, or changed their goals after experiencing bureaucratic challenges (Andersson Vogel et al., 2014; Barton et al., 2008; Flynn et al., 2003; Hazel & Hampson, 2015; Hazel et al., 2008; Jain et al., 2018; Liberman et al., 2021; Miller & MacGillivray, 2002). While Flynn et al. (2003) found that program administrators were not using the same treatment philosophies, Barton et al. similarly noted that “stakeholders had little understanding of the big picture of IAP and TR in the early stages of this project” (2008, p. 50). In the RESET program, Hazel and colleagues note that “…there were fewer consensuses about the aims and objectives of RESET … there had been a lack … communication about what the project was really about and what it is meant to do” (2008, pp. 30–31). Andersson Vogel et al. illustrated the salience of program fidelity, finding that “in the end, [the Leaving Care project] could rather be described as 24 different projects loosely connected by a few mutual grounds than as a structured program or a systematic way of working” (2014, p. 256).

*Theme 6: Administrative duties are not always aligned with direct service delivery, which can hamper program fidelity*.

Additionally, challenges related to service provision were noted throughout the process analyses. For example, paperwork responsibilities were noted as a barrier to service provision, where staff had little time to spend with youth because they had to fulfill administrative tasks (Abrams et al., 2008; Dum & Fader, 2013; Hazel et al., 2008). Similarly, administrative meetings and emails were perceived to take time away from front‐line work and service delivery in the RESET program (Hazel et al., 2008). On the other hand, missing, duplicated, or incorrectly completed paperwork can also waste time, and universal and straightforward documentation procedures (e.g., making sure that staff members know the purpose of forms and case files, or having inter‐agency information sharing systems) were identified as an unfulfilled need in some programs (Andersson Vogel et al., 2014; Barton et al., 2008; Hazel et al., 2012; Iutcovich & Pratt, 1998; Wright et al., 2012). Documentation and case plans were sometimes not made, or transition teams were not in place, leaving youth without proper aftercare/resettlement services (Andersson Vogel et al., 2014; Barton et al., 2008).

##### Caseload feasibility

*Theme 7: Caseload feasibility is variable across aftercare/resettlement programs; however, where caseloads are high, program fidelity may be diminished*.

Findings on staff to participant caseload ratios and casework feasibility were mixed. Three studies reported caseloads that were too high, where low staff to participant ratios impeded program delivery (Citizens' Committee for Children of New York, NY, 2000; Flynn et al., 2003; Liberman et al., 2021). In the Texas implementation of an aftercare/resettlement program, “downsizing of the Houston District Parole Office” was the reason for increased caseloads (Liberman et al., 2021, p. 10). In addition to managing too many cases, aftercare/resettlement workers must juggle numerous responsibilities, which may hamper their ability to provide participants with high quality case management. For example, in the London Youth Reducing Reoffending Program, “Resettlement Brokers were faced with the challenge of trying to secure education or employment opportunities for young people whilst also addressing their wider needs, such as community issues and family relations” (Ipsos MORI, 2012, p. 7). However, another four studies noted that caseloads were small and met program standards (Barton et al., 2008; Hazel et al., 2008; Iutcovich & Pratt, 1998; Wiebush et al., 2005). In Colorado, small caseloads and a team approach to case‐management resulted in “constant interaction and communication around assessment, planning, service delivery, and monitoring issues for each youth … more coordinated interventions for youth … [and], a source of mutual support for staff, which proved critical given the difficult nature of the IAP population” (Wiebush et al., 2005, p. 28).

##### Parents and families

*Theme 8: A high degree of parent involvement is needed for successful program implementation*.

The engagement of parents and families was frequently discussed as a major challenge to successful transition processes or as weak points of the programs. For instance, there was a need for greater parent and family involvement, or for better services dedicated to involving families (Abrams et al., 2008; Jain et al., 2018). In fact, process findings often showed that parents were generally uninvolved in the lives of youth clients, or in the transition processes of their children (Andersson Vogel et al., 2014; Citizens' Committee for Children of New York, NY, 2000; Dum & Fader, 2013; Lindquist et al., 2014; McKay et al., 2014). In some cases parents were identified as problematic and as barriers to program success, especially when they were noted as experiencing behavioral or substance use issues, not fulfilling their roles as guardians (e.g., facilitating condition incompliance, failing to enroll youth in school, or missing court dates), or when they had adversarial relationships with their child or program staff (Dum & Fader, 2013; Lindquist et al., 2014; McKay et al., 2014).

While failures related to parent and family involvement could stem from multiple areas, some findings may provide context towards this problem. For example, where parents are non‐English speakers, it may be difficult for them to engage in the reentry process. Jain et al. note that in an aftercare/resettlement program in California, families and youth of underrepresented ethnicities may have disproportionally experienced communication problems:

“There was a low availability of translators and individuals who could communicate with youth and their families in a culturally responsive way, especially for underrepresented populations like some specific dialects and/or less common Southeast Asian languages” (2018, p. 3673).

In the Targeted Re‐Entry program, the Wisconsin site tackled this barrier by bringing in a volunteer to translate for Spanish‐speaking parents (Barton et al., 2008). Two additional barriers to family involvement stood out as reported in the three sites of the Tribal Green Reentry Program evaluation (McKay et al., 2014). First, parents were reported to be unaware of their roles in the transition process or the ways in which they could be involved in their child's program involvement (McKay et al., 2014). Second, it seemed that parents were sometimes kept in the dark with regard to their child's progress in the aftercare/resettlement program, or they were not notified of changes in transition plans (McKay et al., 2014).

Parent engagement was noted as a success in only three studies: Hazel et al. (2008), McKay et al. (2014), and Sinclair et al. (2021). In the Tribal Green Reentry Initiative, though some parents were found to be unsupportive, others were found to act as proponents of program engagement and transition success; parents were able to emotionally support their children while they were enrolled in the aftercare/resettlement programs, to encourage adherence to program conditions, and to promote youth engagement in programming (McKay et al., 2014). Similarly, Sinclair et al. (2021) found that transition specialists coordinated with client families to promote youth engagement.

#### 5.3.2 Pre‐release stage

In the pre‐release stage, two themes emerged from the data, within two domains: contacts in custodial facilities, and working with custodial staff and institutions.

##### Contacts in custodial facilities

*Theme 9: Minimal contact between aftercare/resettlement workers and youth in custodial facilities compromises the ability to develop strong support relationships*.

According to the theory of change presented in this review, contact between program staff and youth participants should be made early on, ideally at the start of the incarceration period (Bateman et al., 2013; Byrnes et al., 2002; Travis & Petersilia, 2001). Both Iutcovich and Pratt (1998; Philadelphia site) and Liberman and colleagues (2021; California, Texas, and Virginia sites) found that contacts were fewer than expected in the pre‐release stage. For example, though programs aimed to facilitate multiple contacts between case managers and youth in custodial facilities, oftentimes only one contact could be made (Iutcovich & Pratt, 1998; Liberman et al., 2021). Generally, the purpose of these meetings was characterized as “[introducing youth to] the program and services provided” (Liberman et al., 2021, p. 10). Similarly, contact between program staff and the parents/families of clients was also minimal at the pre‐release stage (Iutcovich & Pratt, 1998; Liberman et al., 2021). For example, Iutcovich and Pratt note that “case managers had only one contact documented with 13 of 32 families [in the pre‐release phase], making an average of less than one contact per family” (1998, p. 46). In some cases, it seemed that long distances between community and custodial facilities, or caseloads with clients in multiple far‐apart custodial facilities, were a barrier to contacts between aftercare/resettlement workers and their clients (Barton et al., 2008; Hazel & Hampson, 2015; Hazel et al., 2008; Liberman et al., 2021; Wright et al., 2012). Where caseworkers were assigned from the communities that young custody‐leavers would be released to, they could not travel to custodial facilities to meet; alternatively, if aftercare/resettlement teams were close to custodial facilities but the young person was far from home, it became difficult to coordinate release supports in release destination communities (Barton et al., 2008; Hazel & Hampson, 2015; Liberman et al., 2021; Wright et al., 2012). Minimal contact between case managers and youth in the custodial phase of aftercare/resettlement directly compromises an important component of aftercare/resettlement programming: the development of trusting support relationships that allow youth to buy‐in to programs and be motivated to change (Hazel et al., 2017).

Only one study noted major successes in pre‐release contacts (Wiebush et al., 2005). In the Virginia implementation of the Intensive Aftercare Program (IAP), Wiebush et al. found that:

“In addition to ongoing telephone communication between the case managers and parole officers while the youth was at the institution, [aftercare] parole officers made monthly visits to see the youth and case managers at the institution. The parole staff also contacted parents twice per month while youth were institutionalized, and the institutional case managers traveled to Norfolk periodically to meet with the parents of youth on their caseloads. From the perspective of [aftercare] managers and staff, the improved coordination and communication in planning and service delivery were [the program's] major accomplishments” (2005, p. 41).

For the IAP sites in Colorado, Nevada, and Virginia, visits to participants who were incarcerated were made by case managers or parole officers at least once per month, and “the monthly number of face‐to‐face contacts between [Intensive Aftercare Program] youth and parole officers was at least twice that which occurred in the [treatment as usual] group” (Wiebush et al., 2005, p. 26).

##### Short sentences

*Theme 10: Sentence lengths are often short, leaving very little time for transition planning, preparation activities, or securing post‐release supports*.

A number of studies noted that young people who had shorter sentences received insufficient aftercare/resettlement support while in custody (Barton et al., 2008; Hazel & Hampson, 2015; Hazel et al., 2008; Hazel et al., 2012; Liberman et al., 2021; Wright et al., 2012). For example, in California, Liberman et al. (2021) noted that young people who had shorter lengths of incarceration were not always able to participate in pre‐release programming, and were sometimes not eligible to be enrolled in re‐entry services. Similarly, in the Targeted Re‐Entry program, the Arkansas site found that young people with shorter lengths of incarceration did not have enough time to complete two pre‐release courses that were required for enrollment in the aftercare/resettlement program (Barton et al., 2008). It is clear that, while long periods of incarceration are no doubt harmful to young people, re‐entry preparation takes time and cannot always be completed in one or 2 months.

Further, some studies found that aftercare/resettlement staff simply did not have enough time to put proper supports in place for youth before they were released back into the community. For instance, procuring living arrangements for young custody‐leavers is a particularly important component of positive re‐entry, but short sentences may prevent housing to be secured (Hazel et al., 2012). In addition, practices such as temporary release, while incredibly helpful in gradually bringing young people back to their communities, take a long time to put in place and cannot be offered when sentence lengths are short (Hazel & Hampson, 2015; Wright et al., 2012).

##### Working with custodial staff and institutions

*Theme 11: A lack of communication between custodial staff and aftercare/resettlement workers creates challenges in transition processes and gaps in service*.

Working relationships between aftercare/resettlement program staff or administration and custodial institutions appeared to pose a major challenge in delivering reentry programming. Some studies found that custodial staff had a limited understanding of, support for, or even awareness of the aftercare/resettlement programs (Barton et al., 2008; Wiebush et al., 2005; Wright et al., 2012). For example, in one facility, access to aftercare/resettlement programming was treated as a reward by one custodial facility, rather than as treatment, which hampered the process of preparing for release (Barton et al., 2008).

Some studies noted that poor relationships/partnerships between program staff and custodial facilities contributed to a low number of client‐aftercare worker contacts, delays in creating treatment plans, or difficulties with involving families. A commonly cited issue was the failure of custodial facilities to communicate scheduled release dates to aftercare/resettlement staff (Barton et al., 2008; Iutcovich & Pratt, 1998; Liberman et al., 2021). For instance, Barton and colleagues note that “staff were not even aware of when releases were scheduled, and youths were being released without the [aftercare program] case managers being notified” (2008, p. 45). Similarly, Liberman et al. note:

“Reentry staff were often not informed of when a youth would be released, which hampered their ability to contact youth and their family to develop an individualized plan to guide the transition home. In many cases reentry planning was not able to occur until the youth had returned home” (2021, p. 10).

The need for advanced knowledge of client release dates was directly related to a program's ability to engage youth in program services prior to release, to create effective transition plans, and to allow youth and case managers to develop positive relationships (Barton et al., 2008; Iutcovich & Pratt, 1998; Liberman et al., 2021).

#### 5.3.3 Intensive transition stage

Findings for the intensive transition stage were synthesized into two themes across two domains: service provision during release from custodial facilities, and community partnerships.

##### Service provision during release from custodial facilities

*Theme 12: Aftercare/resettlement programs often fail to ensure youth arrive in the community with immediate supports*.

The continuance of service provision is perhaps the most emphasized component of the intensive transition stage in aftercare/resettlement programming. When youth experience large gaps in services during their transition from custody to their communities, it hampers their ability to lead prosocial lives (Hazel et al., 2017). Several studies noted gaps in care during the time immediately following a youth's release from custody (Abrams et al., 2008; Barton et al., 2008; Citizens' Committee for Children of New York, NY, 2000; Hazel & Hampson, 2015; Hazel et al., 2012; Iutcovich & Pratt, 1998; Sinclair et al., 2021; Wright et al., 2012). For example, one study noted that while youth may have medical care available in custody, “once [youth] are discharged … Medicaid does not automatically continue unless the young person's family meets the standard eligibility criteria, which requires either no wages or very low earnings that do not exceed the minimum wage” (Citizens' Committee for Children of New York, NY, 2000, p. 18). Iutcovich and Pratt found that in the Pennsylvania Intensive Aftercare programs, “once released from institutional placement, approximately 50 percent of the youth were *not* contacted within the first 24 hours” (1998, p. 12). School enrollment immediately after release, or continuation of GED programming, was also noted as a common gap in services (Abrams et al., 2008; Citizens' Committee for Children of New York, NY, 2000; Dum & Fader, 2013; Hazel et al., 2012; Iutcovich & Pratt, 1998).

Some studies noted a need for better service provision surrounding basic life necessities, like food security and housing (Abrams et al., 2008; Hazel & Hampson, 2015; Hazel et al., 2012; Lindquist et al., 2014; Miller & MacGillivray, 2002; Wright et al., 2012). In particular, housing presents a challenge to the re‐entry process, as accommodations are often inappropriate or unstable for young people (e.g., without proper familial support or supervision), far away from custodial sentences (e.g., where youth are returning to their communities, but are placed in far‐from‐home institutions that makes it difficult to schedule pre‐release home visits), not permanent (e.g., where young people are placed in temporary group homes), or difficult to secure (Abrams et al., 2008; Hazel & Hampson, 2015; Hazel et al., 2012; Miller & MacGillivray, 2002; Wright et al., 2012). In the North West Resettlement Consortium, it was found that:

“young people are not currently deemed to be homeless until they exit custody, which means that local authority homelessness departments will not consider their needs until they have left custody … young people are left unsure of their future housing situation throughout the whole custodial period” (Hazel et al., 2012, p. 43).

Related to the issue of gaps in service is the motivation of a young person to change, or lack thereof when proper supports are not put in place. This barrier to a young person's inner change process is illustrated saliently in Hazel and Hampson's evaluation of the Resettlement Broker Project, where a practitioner stated that “…children tend to be full of hope and determination at release, but then typically they become disillusioned when promised services fail to materialize … the moment of opportunity has passed” (2015, p. 84).

Imperative to service continuity are stable and long‐term staff members, who develop relationships with youth and become familiar with their specific needs. In discussing the many hours it takes to develop these relationships, one stakeholder in the RESET evaluation noted “…I don't think it's any coincidence that [the young people are] getting on with their support worker … the worker having the time to build the relationship, I can't stress that enough” (Hazel et al., 2008, p. 53). Where youth were able to develop these long‐term relationships with staff, the relationships were generally cited as being very beneficial (Abrams et al., 2008; Hazel & Hampson, 2015; Hazel et al., 2008; Ipsos MORI, 2012). However, in programs which suffered from high staff turnover, these relationships may have been interrupted or multidisciplinary teams may not have been consistent (Jain et al., 2018). For example, in the Network Aftercare System, Flynn et al. found that a lack of consistent staff was a barrier to relationships between participants and aftercare/resettlement workers, and that “staff need to understand and buy into the IAP model … this level of knowledge and commitment is difficult to attain when turnover is high or when positions remain unfilled” (2003, p. 136).

##### Community partnerships

*Theme 13: Poor relationships with community organizations and a lack of pre‐specified services negatively impacts service access*.

Related to proper service provision are the relationships that aftercare/resettlement programs have with community partners. While youth are ideally linked with community services and agencies prior to release or immediately upon release, this is often not the case. This may be due to a lack of, or generally poor quality, relationships with community service providers and partners, which was noted in a number of studies (Abrams et al., 2008; Barton et al., 2008; Hazel et al., 2012; Iutcovich & Pratt, 1998; Jain et al., 2018; Miller & MacGillivray, 2002; Wiebush et al., 2005). Further, one study noted that community organizations were hesitant to engage with young people who had offended (Hazel et al., 2012).

Difficulties in creating partnerships with specialized services, such as drug and alcohol treatments, family therapy, or services for youth with disabilities, were also noted as a challenge after release from custody (Abrams et al., 2008; Barton et al., 2008; Sinclair et al., 2021; Wiebush et al., 2005). In the Targeted Re‐Entry program, the Arkansas site had “little success involving the faith community in reentry programming”; similarly, in Alabama, “the [Targeted Re‐Entry] program attempted to involve the faith community and Volunteers of America with little success” (Barton et al., 2008, p. 36; p. 23). Further, the age at which youth engage in the program may not be taken into account when organizing supports; for example, Sinclair et al. (2012) noted that “there was … a lack of transition from youth to adult services … one transition specialist mentioned the need for age‐appropriate substance use program, for example, alcoholics anonymous” (p. 10).

#### 5.3.4 Post‐release stage

At the post‐release stage, two themes emerged which represented challenges and successes during two domains of program implementation: follow‐up and long‐term engagement, and relationships with justice personnel in the community.

##### Follow‐up and long‐term engagement

*Theme 14: Service provision is lacking after the intensive transition stage*.

Though aftercare/resettlement services will generally taper off once youth have successfully reintegrated into their communities, aftercare/resettlement programming generally aims to engage youth for, at minimum, multiple months after their release from custody, in order to produce long‐term benefits. However, a number of studies cited problems related to follow‐up care. For example, Abrams et al. (2008) noted that aftercare workers rarely maintained contact with their clients after 6 weeks post‐release, despite program guidelines stating that bi‐monthly contact was to be made for another 2 months; in this case, youth felt unsupported by the program. In the RESET program, aftercare/resettlement staff felt that the program was not long enough, and that they weren't given enough time to deliver program services or to facilitate positive re‐entry experiences for their clients (Hazel et al., 2008). Similarly, Dum and Fader (2013) noted that youth were cut off from various program components before they were ready to be without these services. In the Powelton Aftercare program, aftercare workers felt that they were put “in a no‐win position … if clients were staying out of trouble, that was considered a success that led to termination of services” (Dum & Fader, 2013, p. 798). Youth can also “[age] out and [be] dropped by supports,” leaving them with no further support (Sinclair et al., 2021, p. 10). The problem of clients who age out of youth services has been noted across multiple implementation studies, with many noting that young people who turn 18 or become legal adults are cut off from their re‐entry teams and services (Hazel & Hampson, 2015; Hazel et al., 2008; Hazel et al., 2012; Sinclair et al., 2021; Wright et al., 2012).

Though a lack of program follow up may be due to program deficiencies, such as understaffing or poor leadership, the voluntary nature of many aftercare/resettlement programs can also pose a challenge to long‐term engagement (Citizens' Committee for Children of New York, NY, 2000; Dum & Fader, 2013; Iutcovich & Pratt, 1998; Lindquist et al., 2014). For example, a common problem appears when clients “no‐show”; that is, they do not show up to meetings with aftercare/resettlement staff, or simply refuse to engage in voluntary programming (Citizens' Committee for Children of New York, NY, 2000; Dum & Fader, 2013; Lindquist et al., 2014). In the case of the London Youth Reducing Reoffending Program, there were “instances where young people were removed from the Programme as a result of their lack of willingness to participate and engage” (Ipsos MORI, 2012, p. 5). Similarly, aftercare workers in the Wisconsin Targeted Re‐Entry program felt “frustrated that the youths they served were often, as a group, unmotivated and lacked ambition … they would not hold onto jobs, were reluctant to work hard, and dropped out of training and educational assistance programs” (2008, p. 51). Further, aftercare staff often had no authority to impose consequences or ensure compliance with parole or program conditions, resulting in difficulties with service provision over time (Barton et al., 2008; Dum & Fader, 2013; Iutcovich & Pratt, 1998).

Despite noted challenges, two studies found that follow‐up engagement was successful. Sinclair et al. note that “…the youth reported that the transition specialist helped them stay engaged in school, by checking in and keeping them on track to accomplish their goals, and providing transportation if necessary (e.g., to get to a job interview)” (2021, p. 11). Another study found that, due to positive relationships between clients and aftercare workers, contact lasted for long periods of time post‐release (Ipsos MORI, 2012). Further, another study noted that the voluntary nature of the re‐entry services provided allowed aftercare/resettlement workers to develop a positive rapport with their clients (Hazel et al., 2008).

##### Relationships with justice personnel in the community

*Theme 15: Power imbalances and resentment sometimes lead to inefficient relationships between community‐based criminal justice workers and aftercare/resettlement staff*.

Relationships with justice personnel continued once youth were released into the community, usually with parole officers (POs) who were not directly employed by the aftercare/resettlement programs. Though aftercare/resettlement workers and parole officers would likely have many similar interests or goals for their clients, a number of studies noted poor working relationships between POs or police and program staff that were fraught with tension or resentment (Barton et al., 2008; Dum & Fader, 2013; Hazel et al., 2012; Lindquist et al., 2014). For example, Dum and Fader explained that:

“The relationships between aftercare workers at Powelton and [parole officers] were tenuous at best. Aftercare workers often felt that [parole officers] did not buy into the value of Powelton's services and failed to enforce the mandate for their clients to attend the program. They shared a common belief that POs were lazy and relied too heavily on the aftercare workers to get information about their clients in order to complete paperwork” (2013, p. 794).

Further, in the Wisconsin site of the Targeted Re‐Entry program, relationships between “[parole agents and] TR staff was not always as strong as it had been,” and when meetings between these agents were not being set up by the parole officers, “[Targeted Re‐Entry] staff felt that they could not initiate these” (Barton et al., 2008, p. 47).

In addition to tense relationships, major differences in objectives between enforcement/parole workers and aftercare/resettlement teams seem to hamper the re‐entry process. More specifically, several studies noted that aftercare/resettlement staff felt that POs were too quick to file technical violations or breaches/violations of parole conditions against young people in the community (Barton et al., 2008; Hazel et al., 2012). For example, in the North West Resettlement Consortium, one practitioner noted that:

“…every time [young people] bounce back [into custody] they lose all the provision that we've put in place and it gets harder and harder for us to get it – especially accommodation and education, as you can imagine … so we really need the police on board to understand what our objectives are” (Hazel et al., 2012, p. 35).

Clearly, as housing placements, employment, and other services are already difficult to put in place for youth, it becomes frustrating to aftercare/resettlement staff when young custody‐leavers are quickly sent back to custody for violating conditions of release.

### 5.4 Sensitivity analysis of implementation findings

A sensitivity analysis of implementation themes was conducted to understand the effects of study quality, frequency, and data thickness on qualitative synthesis findings. Results of the analysis are summarized in Table 16.

**Table 16:** Sensitivity analysis of qualitative synthesis findings.

Domain	First author of included studies and bias ratings^a^	Notes	Themes/findings
*Overall findings*			
Funding and program continuance	– Barton (H), Alabama, Arkansas – Iutcovich (H), Allegheny and ‐Philadelphia – Liberman (H), California, Oklahoma, Texas, Virginia – Lindquist (H) – Miller (M)	Evidence supporting this theme is well balanced, with data from multiple high‐quality studies and numerous implementation sites.	*Theme 1: A lack of long‐term funding often results in program termination or poor service delivery*.
Staffing and organization‐level findings	– Barton (H), Alabama, Alaska, Arkansas, Wisconsin – Dum (M) – Flynn (H) – Hazel & Hampson (H) – Hazel, 2008 (H) – Hazel, 2012 (M) – Ipsos MORI (H) – Iutcovich (H), Allegheny and Philadelphia – Jain (H) – Liberman (H), Texas – Lindquist (H) – Miller (M) – Vogel (M) – Wiebush (H), Colorado, Nevada, and Virginia – Wright (M)	Almost every program included in the implementation synthesis contributed findings relevant to staffing and program organization. Though many studies described challenges, positive experiences were also well‐documented.	*Theme 2: Aftercare/resettlement programs experience high levels of staff turnover due to a lack of attractive job features*. *Theme 3: Poor leadership and organizational functioning often compromise aftercare /resettlement program delivery*. *Theme 4: Staff and leadership often show high levels of care and dedication in their work*.
Program fidelity	– Abrams (H) – Barton (H), Alabama, Alaska, Arkansas and Wisconsin – Dum (M) – Flynn (H) – Hazel & Hampson (H) – Hazel, 2008 (H) – Hazel, 2012 (M) – Iutcovich (H), Allegheny and Philadelphia – Jain (H) – Liberman (H), California, Texas, and Virginia – Miller (M) – Vogel (M) – Wright (M)	While theme 5 is strong in terms of both the quality of studies, the frequency of high‐quality studies, and the thickness of data, theme 6 is less robust. Adjustments were made to temper the takeaway of theme 6.	*Theme 5: Program fidelity is often hampered by weak or poorly communicated program theory, aims, and operation*. *Theme 6: Administrative duties are not always aligned with direct service delivery, which can hamper program fidelity*.
Caseload feasibility	– Barton (H), Alabama – Citizens' Committee for Children of New York, NY (M) – Flynn (H) – Ipsos MORI (H) – Iutcovich (H), Allegheny and Philadelphia – Hazel, 2008 (H) – Liberman (H), Texas – Wiebush (H), Colorado, Nevada, and Virginia	Data were relatively non‐descriptive and lacked thickness, often simply mentioning existing caseloads with little discussion of whether they were manageable or not. Analysis did not indicate generalizable findings relating to program ability to maintain caseload feasibility.	*Theme 7: Caseload feasibility is variable across aftercare/resettlement programs; however, where caseloads are high, program fidelity may be diminished*.
Parents and families	– Abrams (H) – Barton (H), Wisconsin – Citizens' Committee for Children of New York, NY (M) – Dum (M) – Hazel, 2008 (H) – Jain (H) – Lindquist (H) – McKay (M) – Sinclair (H) – Vogel (M)	The quality ratings of studies contributing to this theme were almost split; however, in all studies contributing findings to this theme data were thick and relevant to overall conclusions.	*Theme 8: A high degree of parent involvement is needed for successful program implementation*.
**Pre‐release stage**			
Contacts in custodial facilities	– Barton (H), Alabama – Hazel & Hampson (H) – Hazel, 2008 (H) – Iutcovich (H), Allegheny and Philadelphia – Liberman (H), California, Texas, Virginia, and Oklahoma – Wiebush (H), Colorado, Nevada, and Virginia – Wright (M)	Though this theme is supported by high quality studies and data from numerous implementation sites, there were few studies that discussed contacts in the custodial phase of aftercare, making it difficult to conclude whether aftercare programs are generally successful in pre‐release contact levels. The theme statement was appropriately tempered.	*Theme 9: Minimal contact between aftercare/resettlement workers and youth in custodial facilities compromises the ability to develop strong support relationships*.
Short sentences	– Barton (H), Arkansas – Liberman (H), California – Hazel & Hampson (H) – Hazel, 2008 (H) – Hazel, 2012 (M) – Wright (M)	This theme is well balanced; evidence is mostly from studies of high quality.	*Theme 10: Sentence lengths are often short, leaving very little time for transition planning, preparation activities, or securing post‐release supports*.
Working with custodial staff and institutions	– Barton (H), Alabama, Alaska, Arkansas, and Wisconsin – Iutcovich (H), Allegheny and Philadelphia – Liberman (H), California, Oklahoma, Texas, and Virginia – Wiebush (H), Nevada and Virginia – Wright (M)	This theme is very robust: most supporting studies are of high quality, and findings were thick and found in numerous implementation sites.	*Theme 11: A lack of communication between custodial staff and aftercare/resettlement workers creates challenges in transition processes and gaps in service*.
**Intensive transition**			
Service provision during release from custodial facilities	– Abrams (H) – Barton (H), Alaska and Wisconsin – Citizens' Committee for Children of New York, NY (M) – Dum (M) – Flynn (H) – Hazel & Hampson (H) – Hazel, 2008 (H) – Hazel, 2012 (M) – Ipsos MORI (H) – Iutcovich (H) Philadelphia – Jain (H) – Lindquist (H) – Miller (M) – Sinclair (H) – Wright (M)	Study quality is mostly high in this theme, and thick descriptions were available across many studies.	*Theme 12: Aftercare/resettlement programs often fail to ensure youth arrive in the community with immediate supports*.
Community partnerships	– Abrams (H) – Barton (H), Alabama, Arkansas, and Wisconsin – Hazel, 2012 (M) – Iutcovich (H), Philadelphia – Jain (H) – Miller (M) – Sinclair (H) – Wiebush (H), Nevada	Most studies were of high quality, and multiple implementation sites provided data towards this theme.	*Theme 13: Poor relationships with community organizations and a lack of pre‐specified services negatively impacts service access*.
*Post‐release stage*			
Follow‐up and long‐term engagement	– Abrams (H) – Barton (H), Arkansas and Wisconsin – Citizens' Committee for Children of New York, NY (M) – Dum (M) – Hazel & Hampson (H) – Hazel, 2008 (H) – Hazel, 2012 (M) – Ipsos MORI (H) – Iutcovich (H), Allegheny – Lindquist (H) – Sinclair (H) – Wright (M)	This theme is robust and multifaceted (the lack of service provision was supported by two separate findings). Contributions of studies were balanced, and the removal of moderate quality studies did not alter conclusions.	*Theme 14: Service provision is lacking after the intensive transition stage*.
Relationships with justice personnel in the community	– Barton (H), Wisconsin and Alabama – Dum (M) – Hazel, 2012 (M) – Lindquist (H)	The studies of moderate quality that were included in this theme provide the most descriptive findings about the relationship between parole officers and aftercare staff; however, removing these studies would not alter the conclusion that these relationships experienced problems.	*Theme 15: Power imbalances and resentment sometimes lead to inefficient relationships between community‐based criminal justice workers and aftercare/resettlement staff*.

^a^ (H) = high quality study, (M) = moderate quality study.

The influence of each study was examined across the implementation findings for each stage of aftercare/resettlement, and on the implementation synthesis as a whole. No studies were deemed to be overrepresented in the analysis. Further, the proportion of moderate and high‐quality studies was examined. In all stages, the proportion of high‐quality studies that contributed to themes outweighed moderate quality studies. See Table 17. Last, the number of excerpts identified during coding that were considered challenges (*k* = 168) were much greater than those coded as successes (*k* = 120). This may be an indication of the quality of programs surveyed, or may indicate reporting biases, wherein program failures are more likely to be reported than smooth program functioning.

**Table 17:** Descriptive characteristics of qualitative synthesis findings.

	Number of themes studies are present in				
First author/implementation site	Overall findings, *N* (%)	Pre‐release, *N* (%)	Intensive transition, *N* (%)	Post‐release, *N* (%)	Total
Abrams, 2008 (H)	2 (2.2%)		2 (7.7%)	1 (5.6%)	5 (3%)
Barton, 2008 (H)					
– Alabama – Alaska – Arkansas – Wisconsin	6 (6.7%)	2 (6,3%)	1 (3.8%)	1 (5.6%)	10 (6%)
	6 (6.7%)	1 (3.1%)	1 (3.8%)		8 (4.8%)
	4 (4.4%)	2 (6.3%)	1 (3.8%)	1 (5.6%)	8 (4.7%)
	6 (6.7%)	1 (3.1%)	2 (7.7%)	2 (11.1%)	11 (6.6%)
Citizens, 2000 (M)	2 (2.2%)		1 (3.8%)	1 (5.6%)	4 (2.4%)
Dum, 2013 (M)	6 (6.7%)		1 (3.8%)	2 (11.1%)	9 (5.4%)
Flynn, 2003 (H)	5 (5.6%)		1 (3.8%)		6 (3.6%)
Hazel, 2008 (H)	6 (6.7%)	2 (6.3%)	1 (3.8%)	1 (5.6%)	10 (6%)
Hazel, 2012 (M)	1 (1.1%)	1 (3.1%)	2 (7.7%)	2 (11.1%)	6 (3.6%)
Hazel, 2015 (H)	2 (2.2%)	2 (6.3%)	1 (3.8%)	1 (5.6%)	6 (3.6%)
Ipsos, 2012 (H)	3 (3.3%)		1 (3.8%)	1 (5.6%)	5 (3%)
Iutcovich, 1998 (H)					
– Allegheny – Philadelphia	4 (4.4%)	2 (6.3%%)		1 (5.6%)	7 (4.2%)
	4 (4.4%)	2 (6.3%%)	2 (7.7%)		8 (4.8%)
Jain, 2018 (H)	3 (3.3%)		2 (7.7%)		5 (3%)
Liberman, 2021 (H)					
– California – Oklahoma – Texas – Virginia	2 (2.2%)	3 (9.4%)			5 (3%)
	1 (1.1%)	2 (6.3%)			3 (1.8%)
	4 (4.4%)	2 (6.3%)			6 (3.6%)
	2 (2.2%)	2 (6.3%)			4 (2.4%)
Green Reentry^a^	4 (4.4%)				7 (4.2%)
– Lindquist, 2014 (H) – McKay, 2014 (M)	4		1 (3.8%)	2 (11.1%)	7
	1				1
Miller, 2002 (M)	4 (4.4%)		2 (7.7%)		6 (3.6%)
Sinclair, 2021 (H)	1 (1.1%)		2 (7.7%)	1 (5.6%)	4 (2.4%)
Vogel, 2014 (M)	4 (4.4%)				4 (2.4%)
Wiebush, 2005 (H)					
– Colorado – Nevada – Virginia	2 (2.2%)	1 (3.1%)			3 (1.8%)
	2 (2.2%)	2 (6.3%)	1 (3.8%)		5 (3%)
	2 (2.2%)	2 (6.3%)			4 (2.4%)
Wright, 2012 (M)	2 (2.2%)	3 (9.4%)	1 (3.8%)	1 (5.6%)	7 (4.2%)
Total *N*	90	32	26	18	166
Bias rating	*k* = 91^b^				*k* = 167^b^
– High quality – Moderate quality	71 (78%)	28 (87.5%)	19 (73.1%)	12 (66.7%)	130 (77.8%)
	20 (22%)	4 (12.5%)	7 (26.9%)	6 (33.3%)	37 (22.2%)

^a^ If both Lindquist et al. (2014) and McKay et al. (2014) were coded in the same theme, they were only counted as a single study in theme ratios.

^b^ Lindquist et al. (2014) and McKay et al. (2014) were counted separately in bias ratings to better understand their effects on data analysis.

### 5.5 Summary of findings from theory of change literature

Objective 5 focused on updating the theory of change model presented in the protocol for this study; this model shows how aftercare/resettlement programming might work. Figure 1 follows the three stages of aftercare, and summarizes program inputs during each stage, as well as the expected proximal and distal program outcomes. While nothing was removed from the model presented in the protocol, which proved to be an accurate reflection of the programs analyzed in this review, a number of additions were made to provide a more thorough understanding of the causal pathways between program inputs and expected outcomes.

First, a commonly noted focus in the evaluation studies included in this review was the relationship between case managers or aftercare/resettlement workers and youth participants. As the reintegration process should start at the beginning of a custodial sentence, it is important that introductions happen early, so that youth have time to build strong relationships with their case managers or transition teams (Bateman et al., 2013; Hazel et al., 2017). By fostering trust, engagement, and individual responsibility, support workers can help youth feel empowered and motivated in their transition towards a pro‐social life (Bateman et al., 2013; Hazel et al., 2017). Motivation to change is a common barrier to engagement in program activities and conditions, as well as a barrier for meaningful internal change (Barton et al., 2008; Citizens' Committee for Children of New York, NY, 2000; Dum & Fader, 2013; Ipsos MORI, 2012; Iutcovich & Pratt, 1998; Lindquist et al., 2014). By strengthening a participant's confidence in the process of reentry through open communication, trust, and collaborative and tailored transition planning, youth may be more motivated to engage with aftercare/resettlement programming (Hagell et al., 2000; Hazel et al., 2017).

Related to relationship‐building is the concept of gradual transition processes. Some studies included in the implementation synthesis found that program fidelity was compromised due to rushed or poorly executed transitions from custody to the community (Abrams et al., 2008; Barton et al., 2008; Sinclair et al., 2021; Wiebush et al., 2005). The intensive transition process of aftercare/resettlement should ideally be gradual, allowing youth time to adjust to schedule changes, added responsibilities, new relationships, and life “outside.” Further, community partners and transition teams should be aware of release dates in advance so that programming can commence as soon as the youth is released.

### 5.6 Summary of findings from cost assessment studies

Objective 6 focused on synthesizing information related to the costs and savings associated with youth aftercare/resettlement programs. The search resulted in a total of 7 studies which met inclusion criteria, with 8 program sites providing some form of cost assessment (see Table 18). However, the type of assessment conducted (e.g., cost‐effectiveness assessment, comparison of the cost of aftercare vs. no aftercare/previous treatment, etc.), and the amount of detail and information reported varied considerably across the 7 studies, precluding any meaningful synthesis. For example, Bergseth and McDonald (2007) reported one of the more detailed and comprehensive cost–benefit analyses, in which they compared program costs and impacts for both treatment and comparison group youth to various justice system cost estimates to determine whether the program resulted in overall cost savings. Findings noted that youth who participated in the program had fewer justice system contacts and days in restrictive custody than comparison youth, resulting in reductions in system processing costs. Estimates also determined that program costs ($4415 per youth) were fully recovered by approximately 2 years post release, with further system processing cost reductions ($7600 in reduced processing costs were noted at 3 years post release).

**Table 18:** Summary of cost assessment findings.

Author (year)	Program name	Assessment reported	Findings
Beausoleil et al. (2017)	Redemption Reintegration Services (RRS)	Compared cost of service usage between treatment and comparison groups	Program participants generated fewer costs per year than comparison youth.
Bergseth and McDonald (2007)	Reentry Services Project (RSP)	Cost‐benefit analysis comparing program impact to costs related to justice system processing across treatment and comparison group youth	Program participants experienced fewer justice system contacts and days in restrictive placement, resulting in fewer costs; costs associated with implementing the program were fully recovered within three years of release due to the reduction in days in custody.
Cowell et al. (2010)	Serious and Violent Offender Reentry Initiative (SVORI)	Cost‐benefit analysis assessing costs of pre‐release services	No significant difference in associated costs for program participants or comparison youth.
Greenwood et al. (1993)	The Skillman Aftercare Experiment	Compared costs of providing aftercare services via treatment program with standard aftercare services	The implementation of aftercare services in the Detroit site resulted in an overall increase in costs per placement compared to the standard services.
			The implementation of aftercare services in the Pittsburgh site resulted in slightly lower costs compared to standard services.
Hazel et al. (2012)	North West Resettlement Consortium	Cost‐benefit analysis assessing costs related to reoffending, housing, and education of program youth	Savings estimated based on a 20% reduction in reoffending would lead to a 20% savings in the cost per person related to offending. The savings per person are estimated to outweigh the overall costs of the program per year.
Renshaw (2007)	RESET	Provided estimated costs and savings with associated estimates in reduced offending	Proposed estimates suggest between £10,863 and £14,291 of savings over a 9 month period.
Wright et al. (2012)	South West Resettlement Consortium	Cost‐benefit analysis assessing costs related to reoffending, housing, and education of program youth	No significant difference in reoffending found between program and comparison youth. There was a slight decrease in reoffending, which, if assumed to be the result of the program would lead to some degree of cost savings per year (though these do not outweigh program operational costs).

Similarly, Hazel et al. (2012) and Wright et al. (2012) also conducted detailed cost assessment analyses, in which program costs were compared to estimated costs of offending post program participation. Both studies also assessed costs related to reduced likelihood of homelessness and not in education, employment, or training. Overall, Hazel et al. (2012) reported savings of over £9000 per person, per year, primarily due to a reduction in offending. Wright et al. (2012) reported lesser impacts of the program on participant behaviors, leading to lesser cost savings. Though some savings were found (approximately £1959 per person over a year), the savings did not outweigh the program cost per person.

Comparatively, Greenwood et al. (1993) provided only two paragraphs of cost‐related data, and compared the youth placement costs for those in the evaluated programs versus treatment as usual. Findings indicate that the Detroit program site had slightly higher placement costs (approx. $82,030) than their standard services (approx. $76,500), while the Pittsburgh site had slightly lower costs ($37,140 compared to the standard services cost of $37,440). These findings suggest minimal differences in cost compared to standard service provision, but do not provide detailed insight.

## 6 DISCUSSION

### 6.1 Summary of main results

#### 6.1.1 Objective 1

No significant treatment effects were found for studies assessing the outcome of arrest (*k* = 14, OR = 1.044, 95% prediction interval [0.527, 02.075], or incarceration (*k* = 8, OR = 0.806, 95% prediction interval [2.203, 1.433]). Studies examining the outcome of conviction resulted in a positive, significant pooled effect size (*k* = 13, OR = 3.287, 95% prediction interval [1.000, 1.462]. See Table 14. The pooled effect on conviction was not robust; the statistical significance of the aggregate effect was contingent on the inclusion/exclusion of any one of eight studies. Importantly, the number of studies in each outcome group was small, and the set of included studies included primarily non‐peer‐reviewed studies using moderately rigorous research designs and small sample sizes.

#### 6.1.2 Objectives 2 and 3

With respect to study, sample, program component, and treatment delivery as potential moderators of intervention impact, no meaningful pattern of results across outcomes was found (see Table 15). For the arrest outcome, studies rated as having serious bias concerns were related to a negative, statistically significant effect (LOR = −0.360, *z* = −2.099, *p* < 0.05), while studies rated as low bias were related to a positive, nonsignificant effect. The between‐group *Q*‐statistic was significant (*Q*_*B*_ = 7.60, *p* < 0.01), indicating that study bias is an important moderator of treatment impact.

For the conviction outcome, none of the between group heterogeneity statistics were significant, which suggests that none of the variables are important moderators of treatment impact. Subgroup analyses did suggest that several study and sample characteristics were found to have differential subgroup impacts, with positive, significant treatment effects found for studies using a non‐randomized design, studies with sample sizes of 75 or more participants, and studies using primarily ethnic minority samples. In addition, programs that were delivered (at least in part) by community service providers were found to have a significant effect, while those delivered without probations officers were related to a positive effect.

For the outcome of incarceration, no between group heterogeneity statistics were uncovered. Subgroup analyses found that studies rated as having serious bias were related to a statistically significant, negative effect, as were interventions delivered to all male samples.

#### 6.1.3 Objective 4

The qualitative synthesis of process data resulted in 15 themes related to aftercare/resettlement program implementation. Challenges to implementation were presented in included studies much more frequently, resulting in findings that speak more to the failures of aftercare/resettlement programs than to their successes. Overall, findings suggest that aftercare/resettlement programs often suffer from poor communication, coordination, data sharing, and role definition across agencies, leading to a lack of throughcare and ineffective programming. For example, poor communication between custodial staff, aftercare workers/case managers, and community supervision staff meant that youth were less able to prepare for their release, engage in appropriate rehabilitation programming, gain security in the community, and stay motivated to lead pro‐social lives. Aftercare/resettlement programs can aim to reduce these issues by ensuring that relationships between youth and transition teams are consistent (i.e., minimizing changes to team members), developed throughout the entire reentry process (i.e., starting at the beginning of a sentence and lasting through the post‐release phase), and formed on principles of trust and empathy. Further, gaps in services were exacerbated by poor coordination between aftercare/resettlement staff, community partners, parents and families, and justice personnel (in custody and in the community). By increasing data sharing, meetings, and administrative support, these gaps in service may be reduced.

#### 6.1.4 Objective 5

Findings from the analysis of impact evaluations, the qualitative synthesis of implementation findings, and a review of the literature resulted in minor revisions to the theory of change presented in the protocol. Most prominently were the additions of two program components occurring in the pre‐release and intensive transition phases of aftercare/resettlement, as well as additions to the expected proximal and distal outcomes of aftercare/resettlement programming. First, the development of trusting support relationships, which should start at the beginning of a custodial sentence, is represented in themes 2, 4, and 10 of the qualitative synthesis of implementation studies, wherein strong relationships between case managers and youth are needed to create motivation and engagement in youth. Further, emphasis on gradual transition processes, which allow youth to appropriately prepare for life in the community, are echoed by themes 10, 11, and 12 of the qualitative synthesis, which show that overall program implementation is negatively affected when release processes are not coordinated across agencies. Last, expected proximal outcomes were updated to reflect the motivation to maintain prosocial behaviors that results from positive support relationships, and expected distal outcomes now include the overall well‐being of youth.

#### 6.1.5 Objective 6

The systematic search revealed very few studies addressing the issue of cost effectiveness in relation to youth reentry programs. The few studies that were deemed relevant and eligible for inclusion had high heterogeneity with respect to the extent and type of assessment conducted, and the amount of information and detail provided. This precluded any formal analysis and indicates a gap in the existing literature. The lack of existing cost assessment studies may be due to the challenges associated with conducting a full cost‐effectiveness assessment of these types of programs. Youth aftercare/resettlement programs often involve many different services, organizations, and partnerships, with varying frequencies and levels of service usage, in addition to varying program administration costs (Cowell et al., 2010). As well, the costs associated with youth crime can be difficult to estimate due to varying contributing factors, and it is challenging to estimate costs for incidents which have not happened or account for crimes that go undetected by the justice system (Cary et al., 2013). It is often difficult to obtain accurate measures for the numerous cost factors due to the volume and complexity of program and system operations; many cost effectiveness assessments may also occur as an add‐on or afterthought to an impact evaluation, meaning relevant data often are not collected at the outset (Cowell et al., 2010).

The lack of cost assessment data in the literature prevents a complete understanding of the effectiveness and utility of youth aftercare/resettlement programs. While understanding program impacts in relation to recidivism would be beneficial, it is also necessary to assess these impacts in the context of costs. Resources for reentry and rehabilitative services are often limited and must be allocated fairly and efficiently. Understanding if associated program costs are proportionate to achieved outcomes (and potential savings) would be beneficial for program administrators and policymakers.

### 6.2 Overall completeness and applicability of evidence

The resulting intervention analytic sample provided a wide range of information with respect to aftercare/resettlement program characteristics; however, it included a narrow scope of studies with respect to certain characteristics. The included programs were almost exclusively implemented in the United States. Notable differences across countries in terms of legal and justice systems, in addition to cultural and societal distinctions, suggest that program impacts may differ based on geographic location. Further, it is likely that “treatment as usual” groups (i.e., youth who do not receive an aftercare/resettlement intervention or enhanced reentry services) are quite different across countries, states, or sites. These differences may have led to the small proportion of European or UK studies that met our inclusion criteria; as such, the results and recommendations of this study may be less useful in areas where the types of programs that would meet our criteria are not common. The lack of variation in location precluded a detailed investigation of potential geopolitical differences. Additionally, the included studies primarily assessed samples of male youth, with few female participants. Young female offenders may have different experiences and different needs related to their justice system and subsequent reintegration journeys (Espinosa et al., 2013; Jones et al., 2014). As such, aftercare/resettlement programs may have different impacts for female and male youth.

As well, the analyses are exclusively based on official data sources, which may inaccurately represent true rates of recidivism; future evaluations should consider alternative measures of recidivism. Relatedly, the review focuses solely on measures of recidivism as a measure of program success; however there are other aspects of the resettlement experience that may also indicate positive program impacts on factors such as self‐esteem, identity, employment, education, life skills, emotion or behavior management. The completeness of the evidence was also impacted by the considerable heterogeneity observed across the included programs with respect to overall designs, formats, and program components; as such, the conclusions that can be drawn from the pooled analysis are somewhat limited. Nevertheless, the systematic search was designed to be comprehensive and rigorous to uncover a broad scope of evaluation studies in the field, and did yield a large number of studies for potential selection. As such, the findings suggest that the analytic sample reflects the currently available evaluative research, and that more research is needed in the field. Specifically, evaluations should be conducted in varying locations (e.g., the UK, Canada, Australia) and with multiple population types to allow for a more complete assessment of program effectiveness.

With respect to the process/implementation studies, the search was designed to be comprehensive and wide‐reaching. Included studies reported on a variety of implementation factors, in the contexts of both strengths and challenges, with many providing detailed descriptions of the program implementation process and various staff and stakeholder perspectives. Yet, most of the data were derived from staff and program administrators, with minimal input from participant perspectives, creating a gap in the current understanding of program operations. Program participants may have additional insights to offer concerning aftercare/resettlement programs as a result of their receipt of service experiences. Further, other than two studies which discussed language barriers when working with parents, studies generally did not report on barriers or strengths to program implementation as they related to different types of participants, such as ethnic minority youth, immigrant youth, or youth with specific mental or physical health needs. Future process evaluations should aim to examine how service delivery can be optimized for young people/families with differing needs or characteristics, so that aftercare/resettlement programming can be accessible to all types of participants.

As well, we were unable to complete objective 6 with respect to synthesizing cost assessments of aftercare/resettlement programs. Very few studies were determined to have assessed any related costs or savings, and those that did provided a range of assessments and detail, with minimal data provided overall. This resulted in an inability to determine whether programs are cost effective, and whether there is a relationship between program costs and program impacts.

### 6.3 Quality of the evidence

#### 6.3.1 Objectives 1–3

Overall, the set of studies used for the intervention analysis was fairly small, particularly with respect to sample sizes for subgroup analyses and moderator analyses. Moderator analyses typically relied on only 4 or 5 effect sizes for one of the groups, and were not always possible for certain outcomes given particularly small subgroup sample sizes (e.g., only 1 or 2 effect sizes in a subgroup). As well, the set of studies examining the outcome of incarceration was small (*k* = 8 effect sizes).

Within‐study sample size was generally small across the included studies, with total sample sizes at baseline ranging from 61 to 850 participants, and most studies (52%) having 150 participants or less. As such, the set of aftercare/resettlement evaluations may have lacked statistical power to detect true program effects.

As discussed previously, the included studies represent a somewhat narrow focus with respect to participant population and program location. Studies were mostly implemented in the United States and had primarily male samples, impeding the generalizability to non‐U.S. locations/populations and female youth. Given high incarceration rates in the U.S. compared to other western countries, it is perhaps not surprising that aftercare/resettlement programs designed as a follow‐up to a custodial component are more common in the United States. As contextual differences across populations and the various factors that can influence individual experiences, it is possible that programs outside of the U.S. may have differential effects. However, given many of the programs are based on an individual case‐management approach, the programs and services provided are highly individualized, suggesting that generalizability may be possible when translating to other populations.

With respect to risk of bias, all four RCT intervention studies were deemed to be of moderate (“some concerns”) risk, with none receiving a judgment of high, indicating no significant concerns of bias or methodological quality. Of the non‐randomized studies, five studies received a judgment of moderate, four of serious, and two of critical risk of bias. However, these judgments were primarily influenced by concerns related to confounding, as it is not feasible to control for all, or even most potential confounds present for the aftercare/resettlement interventions. As such, the methodological quality of the included studies was strong overall. It is noteworthy that the risk of bias ratings are subject to the information that is reported in the available documents. Some studies did not discuss certain items included in the risk of bias tools, or lacked clarity surrounding certain items, which negatively impacted the overall risk of bias assessments. It is possible that the level of potential bias was overestimated in some studies.

#### 6.3.2 Objective 4

The majority of studies included in the qualitative synthesis of implementation studies were considered to be of high quality, based on ratings on the CASP (2018) qualitative study checklist. Seven studies were rated as moderate quality, while no studies were rated as low in quality. Further, the themes developed based on the qualitative evidence synthesis relied mostly on high quality studies, with approximately 78% of the data contributing to themes coming from high quality studies and approximately 22% coming from moderate quality studies.

#### 6.3.3 Objective 5

An assessment of study quality was not deemed relevant for Objective 5 (theory of change).

#### 6.3.4 Objective 6

Although study quality was not formally assessed with respect to cost assessments, the quality of available data appears to be highly variable; evidence regarding cost effectiveness is severely lacking with very few studies examining economic factors. Of those, the quality is mixed with some providing comprehensive and detailed cost assessment analyses, while others report very brief comparisons of program costs. As such, there is insufficient data with which to draw any formal or useful conclusions regarding the utility of aftercare/resettlement programs in the context of program costs and savings. More rigorous and thorough cost assessments/research is needed to ensure program administrators are using their limited resources in the most efficient way possible and to assist young people who have offended in their community transition.

### 6.4 Potential biases in the review process

The systematic search was designed to be comprehensive and to capture all relevant published and non‐published evaluations. However, in any systematic review there is always a risk of some studies being missed. This can be due to technical errors or nuances in the various databases that results in certain hits not being properly identified, abstracts which fail to accurately describe a study's aims, or human error in missing the selection of a relevant study. Additionally, the gray literature presents other challenges with respect to scope. There may be numerous websites, organizations, journals, etc., that we were unaware of that could have contained relevant studies. Given the scope of these potential sources, it is not feasible to know of and search every possible option. However, the present search did exhaust multiple gray literature options, including 23 journals, 4 meeting archives, 11 organization websites, 3 open access journal websites, and the CVs of 8 known researchers in the field.

As well, 51 studies identified for retrieval were unable to be collected. Though many of these appear to be conference presentations or theses/dissertations related to documents we did retrieve, there may be others that might have met the inclusion criteria had our inter‐library loans department been able to obtain the document. Unobtained studies are listed under “References to studies awaiting classification” in the reference list.

While coding studies that presented implementation data, some decisions may have been subjective; to combat potential biases, the set of studies was split in half and each coder conducted initial data extraction on one half of the sample, then validated coding on the other half. To address similar concerns in the qualitative analysis and promote replicability and validity, descriptive characteristics of each theme and study were reported and a sensitivity analysis was conducted to ensure that findings were not skewed by lower quality, sparse, or “thin” data. Last, it is possible that implementation studies were more likely to report negative findings, rather than findings that suggest no problems, perhaps resulting in themes that were skewed toward program challenges.

### 6.5 Agreements and disagreements with other studies or reviews

As described earlier, three prior reviews have presented meta‐analyses on the impacts of aftercare/resettlement programs (Bouchard & Wong, 2018; James et al., 2013; Weaver & Campbell, 2015). Table 19 presents an overview of these three studies and their main findings, in comparison to the current research. The four studies used somewhat different inclusion criteria, although all required comparison group designs and an aftercare/resettlement program that occurred during or following a custodial stay. The earliest study (James et al., 2013) found a small, positive, significant overall treatment impact, while Bouchard and Wong (2018) found a positive and significant impact of programs on alleged offenses (i.e., negative police contacts, arrest, charges). Conversely, Weaver and Campbell (2015) reported a positive but non‐significant impact of treatment, while Bouchard and Wong (2018) found a negative, non‐significant impact of programs on convicted offenses (convictions and incarcerations).

**Table 19:** Comparison of the current study to prior meta‐analyses.

Study	# of program sites included (effect sizes)	Aftercare program definition	Key inclusion/exclusion criteria	Search sources	Outcomes	Findings
James et al. (2013)	22 (22)	Re‐entry or aftercare interventions that included a treatment modality (skills training, counseling, cognitive behavioral therapy).	– RCT or quasi‐experiment designs – Included a custodial stay – Max. mean age of 25 – No intensive supervision programs – Reported recidivism data – No restrictions on publication type	20 sources (databases, Google, websites); specialist online search engines, reference lists, journals, dissertation abstracts	Any new conviction/adjudication or arrest	Small, positive, significant effect
Weaver and Campbell (2015)	30 (30)	Aftercare consisted of monitoring, supervision, and various services in the community post‐release from custody. Must have included a community‐based component.	– Included a control group – Youth were committed to youth facility or detention center – Reported recidivism data	18 electronic sources, reference lists	Any committed offense	Positive, but non‐significant main finding
Bouchard and Wong (2018)	15 (24)	Program must have included a community‐based component.	– Included a control group – Published 1990–2015 – Reported recidivism measures – Included a custodial stay – Not targeting a specific subpopulation – Comparison group not a standard probation program	20 electronic databases, journals, author CVs, websites	Alleged offenses (arrests, charges, referrals, court/police contacts)Convicted offenses (convictions, sustained petitions, adjudication, incarceration)	Positive, significant effect for alleged offenses Negative, non‐significant effect for convicted offenses
Current study	19 (33)	Aftercare services provided at any of the pre, during, or post‐transition phases; must have included a custodial stay.	– Published 1992–2023 – Included a control group – Reported recidivism measures – Mean age <18 – Not targeting a specific population – Implemented in North America, Western Europe, Australia, New Zealand	26 electronic databases, 23 journals, author CVs, websites, meeting archives, open access search engines	Any new arrest, conviction, or incarceration	Positive, non‐significant findings for arrest and conviction; negative, non‐significant finding for incarceration

In comparison, the current review finds a positive, non‐significant overall effect, and positive, non‐significant effects for outcomes of arrest and conviction. Treatment impacts on conviction were significant but volatile, with findings contingent on the inclusion or exclusion of 62% of the sample of studies. A negative, non‐significant treatment effect was found for the outcome of incarceration.

Taken together, the current study fits into the context of the current literature by re‐establishing the generally positive, but overall small and lackluster results of aftercare/resettlement programs on recidivism outcomes. The importance of internal change throughout the transition process has been stressed in the literature (Bateman & Hazel, 2018; Hazel et al., 2017, 2022). This is echoed by the findings of our qualitative synthesis analysis of process evaluation studies, which found that participant motivation and engagement was a notable challenge during program implementation. As noted by all prior reviews, additional evaluations of (ideally well‐implemented) aftercare/resettlement programs, using rigorous research designs, is essential.

## 7 AUTHOR'S CONCLUSIONS

### 7.1 Implications for practice

Current evidence suggests no notable effects of youth aftercare/resettlement interventions on outcomes of recidivism. The current data do not provide insight regarding the importance of specific intervention components (e.g., housing, individual therapy); the exceptions were that programs involving the provision of services from community‐based providers, and those *not* involving probation officers, resulted in reduced rates of re‐convictions. The lack of consistent pattern of results with respect to both overall treatment effects and moderators of treatment impact lead to a lack of notable recommendations for practice with respect to program design/components. Based on the many challenges uncovered in the analysis of implementation studies, it is not surprising that aftercare/resettlement programs fail to demonstrate strong evidence of effectiveness. Greater emphasis on internal youth processes, such as motivation to change and improved self‐esteem, the development of relationships between transition teams and youth, as well as enhanced coordination and cooperation across agencies, and attention to social and environmental service needs of the youth (e.g., housing, employment/income support, food security), is vital for future practice.

### 7.2 Implications for research

Results of the current study indicate that additional work is needed for a more thorough understanding of the impact of youth aftercare/resettlement programs on recidivism. This is particularly true with respect to locations outside of the U.S. and for female populations; research in these areas is lacking and the field would benefit from additional insight. For example, rigorous research in the UK context would be a valuable addition to the field. Relatedly, increased research focusing on specific program components or approaches would provide greater information regarding what is important for program success. Existing aftercare/resettlement programs take a variety of approaches and utilize varying formats and services, which results in considerable levels of heterogeneity in pooled analyses. Greater evaluation research in the field will allow for a larger sample size and the ability to pool programs based on approach, format, or component and provide a more detailed level of understanding.

Future research should also consider the implementation process, as few evaluations included process evaluations. Understanding the strengths and challenges encountered during program implementation and the extent of implementation fidelity is necessary for a greater understanding of program impacts, and why or why not a program is successful. Additional research might include quantitative analyses of the frequency of contacts and services received; it may be that program success is moderated by the intensity of services received by participants. Further, qualitative impact findings should be reviewed to better understand the internal processes of change that youth experience during reentry, and the impact that programming has on overall well‐being and security. This might include qualitative interviews with program participants to understand their specific experiences and how the program may have helped (or not) in their resettlement process. While recidivism is an important goal of aftercare/resettlement efforts, these programs can also have various impacts on other factors related to youths' transitions back to the community. Though some evaluations do assess various outcomes other than recidivism (e.g., self‐esteem, identity, employment, education, life skills, emotion or behavior management), these are reported on less consistently than offending outcomes, making them difficult to synthesize. Examining additional outcomes is important for gaining an understanding of the wider‐reaching impacts (or lack thereof) of these programs. Last, future evaluation studies, whether they focus on impact or implementation, should aim to include data on program theory and to further develop the theory of change for aftercare.

## CONTRIBUTIONS OF AUTHORS

The protocol was developed by Jennifer S. Wong (JSW) and Chelsey Lee (CL) with contributions from Natalie Beck (NB). CL and NB conducted the systematic literature search. Decisions on inclusion for impact evaluation studies were made by all three team members, with conflicts resolved through discussion until consensus is reached. Quantitative and qualitative study coding was carried out by CL and NB. The estimation of effect sizes was done by CL and NB and validated by JSW. Quantitative analyses were conducted by JSW and validated by CL. Qualitative analyses were conducted by NB and validated by CL. Report writing was conducted by all three team members.

## DECLARATIONS OF INTEREST

The lead author has been involved in a prior systematic review on this topic. Otherwise, there are no known conflicts of interest for the research team. Forms attached.

## PRELIMINARY TIMEFRAME

Following is a timetable presenting dates by which the key tasks were completed:

- Searches for eligible studies: December 29, 2022–January 13, 2023
- Screening/compiling results from the literature search and document retrieval: February 11, 2023
- Extraction of data from eligible research reports: March 15, 2023
- Statistical analysis: April 30, 2023
- Preparation of the final review report: June 5, 2023
- Preparation of the revised review: October 2, 2023

## PLANS FOR UPDATING THE REVIEW

The rate of publication of rigorous studies in this field is likely to be slow. JSW will monitor the literature in the field every 5 years and will coordinate an update of the review once sufficient high‐quality studies become available.

## DIFFERENCES BETWEEN PROTOCOL AND REVIEW

Few differences occurred in the final review compared to the initial protocol. We were unable to conduct any formal summative analysis regarding programs costs due to a lack of data; had the search resulted in a larger sample, more analysis would have been possible. In addition, while we anticipated identifying research focused on antisocial behavior and aggression, only one study was found meeting inclusion criteria that examined this outcome.

Additionally, we did not contact first authors of included studies; many of the authors' CVs were hand searched and additional contact was deemed unnecessary. Two journals were unavailable for searching (Canadian Journal of Criminology and Canadian Journal of Criminology and Corrections). The use of NVivo was added to the coding and analysis process to help facilitate the qualitative analysis. Relatedly, the coding schemes for each analysis changed slightly through the removal or addition of several variables to better capture the specific data available.

## SOURCES OF SUPPORT

### Internal sources

None.

### External sources

We are grateful to the Youth Endowment Fund for financial support for this review.

## Supporting information

Supporting information.

## Footnotes

1 We note that while North American literature typically uses the terms “youth” and “adolescent,” UK literature more frequently uses the terms “children” and “young person” to refer to individuals in the same general age category.

2 In the UK, a young person's “transition from custody to the community is commonly known as ‘resettlement’, with practitioner involvement known as resettlement support” (Hazel, 2022). In North American literature, this process is typically referred to as “aftercare” or “reentry,” and is often framed as a program, practice, or intervention. In an effort to be inclusive, herein we use all terms synonymously.

3 While this review primarily examines program effectiveness with respect to recidivism, we note that recidivism is not a comprehensive measure of effectiveness; programs often target other outcomes such as self‐esteem, self‐identity, housing, employment, education, relationships, non‐criminal behaviors, etc.

4 While outcomes of violent behavior and self‐reported criminal behavior were eligible for inclusion, no studies reporting on these types of outcome measures met the other review inclusion criteria.

5 As per Bouchard and Wong (2022), meta‐analyses of correctional intervention effectiveness that use aggregated measures of crime/recidivism (i.e., multiple measures combined) versus disaggregated measures (e.g., arrest, conviction, incarceration) may provide an incomplete and potentially misleading assessment of treatment effectiveness.

6 Once data were extracted from studies and coded into a priori variables in Excel, NVivo 12 was used to further code the data (where emergent variables, or “buckets,” were created) and to conduct a thematic analysis.

7 Although the quantitative studies were scored separately on either the RoB‐2 or ROBINS‐I tools, risk of bias was collapsed into a single variable for the purpose of subgroup analysis. Categories across the two tools were recoded to correspond to low, moderate, and high risk of bias. As the ROBINS‐I has five categories compared to the three in the RoB‐2, “serious” and “critical” were combined. No studies had an overall assessment of “no information,” and as such, this category was not included.

8 Specifically, 31%–100% vs. <30% White.

9 Other intervention characteristics coded but not used in the analyses due to having fewer than 3 effect sizes per group included: behavioral/emotional skills, academic skills, vocational skills, and parental involvement.

10 Other program delivery options coded but not used in the analyses due to having fewer than 3 effect sizes per group included: case managers, counselors, police, and volunteers.

11 For example, in synthesizing qualitative findings regarding interagency communication, one might conclude that case management data are/are not shared consistently between stakeholders within aftercare/reentry programming. However, in assessing the coded data which contribute to this finding, one might find that a single study provided most of the evidence regarding interagency communication, and that the removal of this study would alter the conclusion.

12 This excludes sources we were unable to achieve total hit counts for, such as programs of conference proceedings, author CVs, and included study reference lists.

13 These include Bergseth (2010), Bergseth and McDonald (2007), Bouffard and Bergseth (2009), James (2015), Lattimore et al. (2012), Lattimore and Visher (2009), and Liberman et al. (2021).

14 Four studies reported on multiple implementation sites. Barton et al. (2008) reported on four program sites, Iutcovich and Pratt (1998) reported on two program sites, Liberman et al., 2021 reported on two program sites, and Wiebush et al. (2005) reported on three program sites.

15 Further, four studies conducted implementation evaluations quantitatively, with no qualitative component. These studies were ultimately excluded as they did not measure commensurate outcomes that could be synthesized in a rigorous manner.

16 The 19 publications included in the process analysis represented 18 implementation studies. One study, an evaluation of the Green Reentry program, was presented in two separate publications: Lindquist et al. (2014) and McKay et al. (2014). Each publication was rated separately on the CASP qualitative study checklist.

17 More specifically, the study examines a sample of offenders (both treatment and control) living in cottages (as opposed to a more typical closed custody setting) for 9–12 months; all offenders participated in rehabilitative programming. The resettlement program involved a 6‐week period in which the treatment group participants were permitted to leave to go to work/school during the day. In other words, this program occurs while offenders are in custody, with a goal to helping them more successfully reintegrate upon release. While this intervention meets the meta‐analysis criteria for inclusion, it is the only program in the set that does not also include a post‐release component.
