# Supplementary material for: The effects of aftercare/resettlement services on crime and violence in children and youth: A systematic review
Source: Campbell Syst Rev. 2024 May 25;20(2):e1404. doi: 10.1002/cl2.1404 (PMC11128035; doi:10.1002/cl2.1404)
Supplement: Supplementary file 1 — Supporting information. [file CL2-20-e1404-s001.docx]

## APPENDICES

Following is a draft coding form for the review; to be revised as necessary during the coding process.

**Appendix 1. CASP Qualitative Study Assessment**

Studies were rated on each question as one of the following: (1) yes, (2) can’t tell, or (3) no. Ratings decisions were made using additional guidelines, found in the CASP (2018) checklist document.

SECTION A: Are the results valid?

1. Was there a clear statement of the aims of the research?
2. Is a qualitative methodology appropriate?
3. Was the research design appropriate to address the aims of the research?
4. Was the recruitment strategy appropriate to the aims of the research?
5. Was the data collected in a way that addressed the research issue?
6. Has the relationship between researcher and participants been adequately considered?

SECTION B: What are the results?

1. Have ethical issues been taken into consideration?
2. Was the data analysis sufficiently rigorous?
3. Is there a clear statement of findings?

SECTION C: Will the results help locally?

1. How valuable is the research?

**Appendix 2. Draft Coding Form**

| **Publication characteristics** | |
| --- | --- |
| *Variable* | *Coding details* |
| Author (date) | String variable |
| Outcome number | No specification (e.g., 1, 2, 3, etc.) used when one study contributed more than one outcome effect) |
| Study publication year | No specification (e.g., 1995, 2001, etc.) |
| Publication type | 0 = Journal article  1 = Book chapter  2 = Report  3 = Dissertation/thesis |
| Peer review | 0 = No  1 = Yes |
| Year(s) of study | No specification (e.g., 2002-2006, 2015, etc.) |
| Source of funding | String variable |
| General intervention characteristics | |
| *Variable* | *Coding details* |
| Program name | String variable |
| Program delivery year(s) | No specification (e.g., 1995, 2001, etc.) |
| Program delivery start year | No specification (e.g., 2004, 2013, etc.) |
| Location | 0 = North America  1 = UK  2 = Western Europe  3 = Australia/New Zealand |
| Target population | 0 = Referred/selected for the program  1 = Open to all youth |
| General program description | String variable |
| Program theory of change | String variable; any information concerning proposed pathways to desired outcomes for participants |
| Intervention components | |
| *Variable* | *Coding details* |
| Program contained behavioral/emotional skill component? | 0 = No  1 = Yes |
| Program contained academic component? | 0 = No  1 = Yes |
| Program contained vocational component? | 0 = No  1 = Yes |
| Program contained life skills training component? | 0 = No  1 = Yes |
| Program contained housing component? | 0 = No  1 = Yes |
| Program contained individual therapy component? | 0 = No  1 = Yes |
| Program contained group therapy component? | 0 = No  1 = Yes |
| Program contained family therapy component? | 0 = No  1 = Yes |
| Program contained mentoring component? | 0 = No  1 = Yes |
| Program contained case management component? | 0 = No  1 = Yes |
| Program contained substance use treatment component? | 0 = No  1 = Yes |
| Any other program components not listed | String variable |
| Program duration | No specification (e.g., 8 weeks) |
| Parent/family involvement | 0 = No  1 = Yes |
| Setting | 0 = Community only  1 = Custody only  2 = Custody and community component |
| Delivered by probation officers? | 0 = No  1 = Yes |
| Delivered by case managers? | 0 = No  1 = Yes |
| Delivered by police? | 0 = No  1 = Yes |
| Delivered by counsellors? | 0 = No  1 = Yes |
| Delivered by program staff? | 0 = No  1 = Yes |
| Delivered by students? | 0 = No  1 = Yes |
| Delivered by volunteers? | 0 = No  1 = Yes |
| Delivered by various community service providers? | 0 = No  1 = Yes |
| Delivered by others? | 0 = No  1 = Yes |
| Risk assessment tool used? | 0 = No  1 = Yes |
| Specific risk assessment tool | No specification (e.g., Youth Level of Service/Case Management Inventory (YLS/CMI)) |
| Participation mandatory? | 0 = No  1 = Yes  2 = Mixed |
| Type of offender/risk level | 1 = Low risk  2 = Medium risk  3 = High risk  4 = Medium/high risk  5 = Low/medium/high risk |
| Timing of services | 1 = Post-release only  2 = Pre-release only  3 = Both pre- and post-release |
| Cost data | |
| Cost of previous services/treatment as usual | String variable |
| Annual program cost | No specification ($) |
| Number of clients served | No specification (n) |
| Staffing costs | No specification ($) |
| Average cost per participant | No specification ($) |
| Reported estimates of incarceration costs per person | No specification ($) |
| Other reported criminal justice system costs | No specification ($) |
| Cost-benefit analysis findings | String variable re: main outcomes of CBA |
| Level of savings | String variable re: any savings determined by the CBA |
| Notes re. cost data and any potential for bias | String variable re. any potential bias re data/methods used to estimate intervention cost, participants served, intervention benefits, or anything else |
| CHEC-list score | Score on the Consensus on Health Economic Criteria (CHEC-list) and number of applicable checklist items |
| % on the CHEC-list | Score/# of applicable items |
| Additional notes re. program characteristics | String variable |
| Qualitative study characteristics | |
| *Variable* | *Coding details* |
| Author (date) | String variable |
| Program name | String variable |
| General program description | String variable |
| Sample information | String variable (e.g., types of participants, number of participants) |
| Timing of services | 1 = Post-release only  2 = Pre-release only  3 = Both pre- and post-release |
| Data collection method: qualitative interviews | 0 = No  1 = Yes |
| Data collection method: program materials | 0 = No  1 = Yes |
| Data collection method: program case records | 0 = No  1 = Yes |
| Data collection method: questionnaires | 0 = No  1 = Yes |
| Data collection method: focus groups | 0 = No  1 = Yes |
| Data collection method: observations | 0 = No  1 = Yes |
| Method of data analysis for process evaluation | String variable (e.g., ethnographic analysis, thematic analysis) |
| Study design | 1 = qualitative  2 = mixed methods  3 = quantitative |
| Data sources: program participants | 0 = No  1 = Yes |
| Data sources: case managers | 0 = No  1 = Yes |
| Data sources: probation officers | 0 = No  1 = Yes |
| Data sources: community partners | 0 = No  1 = Yes |
| Data sources: counsellors | 0 = No  1 = Yes |
| Data sources: parents | 0 = No  1 = Yes |
| Data sources: program administration | 0 = No  1 = Yes |
| Data sources: employers | 0 = No  1 = Yes |
| Data sources: school staff | 0 = No  1 = Yes |
| Data sources: custodial staff | 0 = No  1 = Yes |
| Data sources: policy maker | 0 = No  1 = Yes |
| Main process outcome assessed | String variable (e.g., implementation, case management) |
| Process evaluation quality appraisal (CASP checklist) | |
| *Variable* | *Coding details* |
| Was there a clear statement of the aims of the research? | 1 = Yes  2 = Can’t tell  3 = No |
| Is a qualitative methodology appropriate? | 1 = Yes  2 = Can’t tell  3 = No |
| Was the research design appropriate to address the aims of the research? | 1 = Yes  2 = Can’t tell  3 = No |
| Was the recruitment strategy appropriate to the aims of the research? | 1 = Yes  2 = Can’t tell  3 = No |
| Was the data collected in a way that addressed the research issue? | 1 = Yes  2 = Can’t tell  3 = No |
| Has the relationship between researcher and participants been adequately considered? | 1 = Yes  2 = Can’t tell  3 = No |
| Have ethical issues been taken into consideration? | 1 = Yes  2 = Can’t tell  3 = No |
| Was the data analysis sufficiently rigorous? | 1 = Yes  2 = Can’t tell  3 = No |
| Is there a clear statement of findings? | 1 = Yes  2 = Can’t tell  3 = No |
| How valuable is the research? | 1 = High value  2 = Moderate value  3 = Low value |
| Overall quality of the study | 1 = Low quality  2 = Moderate quality  3 = High quality |
| Limitations noted | String variable (e.g., staff members reluctant to share data with evaluators) |
| Process evaluation findings | |
| *Variable* | *Coding details* |
| Communication | String variable (e.g., lack of communication between service providers) |
| Continuity of care | String variable (e.g., gaps in services noted) |
| Service provision | String variable (e.g., intervention programming delivered) |
| Service/organization partnerships | String variable (e.g., partnerships with employment offices in the community) |
| Safety concerns | String variable (e.g., caseworker visits to high-risk areas) |
| Underutilized/overtaxed services | String variable (e.g., underutilization of community partners) |
| Contacts | String variable (e.g., contacts between aftercare worker and participant) |
| Other challenges | String variable |
| Leadership | String variable (e.g., strong leadership in aftercare program) |
| Staffing | String variable (e.g., inadequate training noted; clear roles/duties/responsibilities laid out) |
| Case management | String variable (e.g., quality of case plans) |
| Resources/funding | String variable (e.g., caseworker to participant ratios, staff turnover and retention) |
| Casework feasibility | String variable (e.g., number of cases per caseworker) |
| Administrative/documentation | String variable (e.g., paperwork expectations) |
| Any additional qualitative findings | String variables (coded inductively) |
| Impact evaluation study characteristics | |
| *Variable* | *Coding details* |
| Research design | 0 = RCT  1 = QE w/matched comparison group  2 = QE w/weakly matched comparison group |
| Random assignment | 0 = No  1 = Yes |
| Control group type | 0 = RCT control  1 = Waitlist control  2 = Matched control |
| Control group description | String variable (e.g., youth returning from custody without any reentry services) |
| Pretest | 0 = No  1 = Yes |
| Quality of implementation: Any problems noted | 0 = No problems  1 = Minor problems  2 = Major problems |
| Researcher involvement | 0 = Evaluation only  1 = Involved in delivering intervention  2 = Involved in developing intervention  3 = Developed and delivered intervention |
| Declarations of interest among primary researchers | 0 = No  1 = Yes |
| Notes re. research design and any potential for bias | String variable describing any potential concerns with the research design that may lead to biased findings. |
| Score on the ROBINS-I or ROB-2 | 0 = low risk  1 = medium risk  2 = high risk |
| Sample characteristics | |
| *Variable* | *Coding details* |
| Total N at baseline | No specification (e.g., n=64) |
| Description of treatment group | String variable |
| N of treatment group at baseline | No specification (e.g., n=64) |
| Description of control group | String variable |
| N comparison group at baseline | No specification (e.g., n=23) |
| Participant age range | No specification (e.g., 14-21 years) |
| Approximate mean age | No specification (e.g., 18.2 years) |
| SD of mean age | No specification (e.g., 0.9 years) |
| Gender mix | No specification (e.g., 74% male) |
| Gender | 1 = All female (90%+)  2 = Mostly female (70-89%)  3 = Mixed (31-69%)  4 = Mostly male (70-89%)  5 = All male (90%+) |
| Racial mix | No specification (e.g., 17% Black, 24% Hispanic) |
| Ethnicity mix | No specification (e.g., 82% Chinese) |
| Ethnicity | 1 = All white (90%+)  2 = Mostly white (70-89%)  3 = Mixed (31-69% white)  4 = Mostly minority (<30% white) |
| Unit of assignment | 0 = Individual  1 = Group |
| Outcomes adjusted for pre-test differences? | 0 = No  1 = Yes |
| Direction and magnitude of initial differences between treatment and control group | String variable (e.g., treatment group significantly higher in proportion male than control group) |
| Attrition from treatment and control groups on recidivism outcome | String variable (e.g., 26% from pretest to post-test) |
| Notes re. study sample and any potential for bias | String variable describing any potential concerns with the study sample that may lead to biased findings (e.g., major attrition in the control group.) |
| Outcome measure | |
| *Variable* | *Coding details* |
| Outcome measure name | String variable (e.g., self-reported crime, Y-OQ, police reports) |
| Type of outcome | 1 = Conviction  2 = Incarceration  3 = Arrests/police contact  4 = Self-reported delinquency |
| Source of measure | 0 = Official report  1 = Self-report  2 = Other report (parent, case manager, etc.) |
| Direction of measure | 0 = Increase in score is good  1 = Increase in score is bad |
| Measurement | 0 = Dichotomous  1 = Continuous |
| Findings | String variable (e.g., significant reduction in social problems from pre-test to post-test, no significant differences from post-test to 6-month follow-up) |
| Notes re. outcome measures and any potential for bias | String variable describing any potential concerns with the study outcome measures that may lead to biased findings (e.g., change in measurement approach from pretest to post-test). |
| Time of post test | String variable (e.g., on last day of program) |
| Time of follow-up(s) | String variable (e.g., 6 months following post-test) |
| Time used for effect size calculation | 0 = Post-test  1 = Follow-up |
| Category of time used for effect size calculation | 1 = Less than 6 months  2 = 6-11 months  3 = 12-17 months  4 = 18+ months |
| Impact evaluation findings | |
| *Variable* | *Coding details* |
| Treatment group mean at pretest | No specification (e.g., 4.7) |
| Treatment group SD at pretest | No specification (e.g., 1.2) |
| Treatment group % at pretest | No specification (e.g., 21%) |
| Treatment group N at pretest | No specification (e.g., 144) |
| Treatment group mean at post-test | No specification (e.g., 4.7) |
| Treatment group SD at post-test | No specification (e.g., 1.2) |
| Treatment group % at post-test | No specification (e.g., 21%) |
| Treatment group N at post-test | No specification (e.g., 144) |
| Pre to post effect on treatment group | 0 = Negative  1 = Positive  2 = Null |
| Which group has better outcomes? | 0 = Treatment  1 = Control  2 = Neither |
| Between-group post-test analysis | No specification (e.g., *t* = 2.66, *p*<.01) |
| Control group mean at pretest | No specification (e.g., 4.7 (1.2)) |
| Control group SD at pretest | No specification (e.g., 1.2) |
| Control group % at pretest | No specification (e.g., 21%) |
| Control group N at pretest | No specification (e.g., 144) |
| Control group mean at post-test | No specification (e.g., 4.7 (1.2)) |
| Control group SD at post-test | No specification (e.g., 1.2) |
| Control group % at post-test | No specification (e.g., 21%) |
| Control group N at post-test | No specification (e.g., 144) |
| For Odds ratio calculation (if applicable) | |
| *Variable* | *Coding details* |
| Treatment group yes | No specification (e.g., 64) |
| Treatment group no | No specification (e.g., 36) |
| Control group yes | No specification (e.g., 64) |
| Control group no | No specification (e.g., 36) |
| Effect size calculation method | |
| *Variable* | *Coding details* |
| Type of data ES based on | No specification (e.g., *t*, means/SDs, etc.) |
| Method of ES calculation | No specification (e.g., Wilson’s OR calculator) |
| Effect size calculated | |
| *Variable* | *Coding details* |
| Odds ratio/*d* | No specification (e.g., 0.3967) |
| Log odds ratio | No specification (e.g., -0.9246) |
| Standard error | No specification (e.g., 0.6542) |
| Variance | No specification (e.g., 0.4280) |
| 95% CI lower | No specification (e.g., 0.1100) |
| 95% CI upper | No specification (e.g., 1.4300) |
| Effect size overview | |
| *Variable* | *Coding details* |
| Pre-test adjusted | 0 = No  1 = Yes |
| Sign correct | 0 = No  1 = Yes |
| Type of data ES based on | 1 = pre & post means & SDs - 2 groups  2 = pre & post means & SDs & t-statistic - 1 group  3 = pre & post means & SDs & F-statistic - 1 group  4 = post-only means & SDs - 2 groups  5 = B, SD of DV, n's - 2 groups  6 = % yes pre/post - 2 groups  7 = % yes pre/post = 1 group  8 = post Ns (unequal) + F - 2 group  9 = post Ns (unequal) + t - 2 group  10 = % yes post - 2 groups |
| Needs transformation | 0 = No  1 = Yes |

**Appendix 3. Search strategies**

**Table A1. Electronic Platform Searches**

| **Electronic Platforms** | | | | | |
| --- | --- | --- | --- | --- | --- |
| **EBSCO** | **Included databases:**   - Academic Search Premier - Criminal Justice Abstracts - Education Source - ERIC - Medline - PsycArticles - PsycBooks - PsycInfo - Social Sciences Abstracts - Social Sciences Full Text | **Search fields:**  Title, abstract, subject terms | **Search limiters**:  Date: January 1992 – January 2023 | **Date searched:** January 11-12, 2023 | **Total hits**: 19,684 |
|  | **EBSCO search string/terms used**  (AB ( youth* or juvenile* or adolesc* or teen* or "young offender*" or "young people" or "young person*" or child* or "early offender" ) OR TI ( youth* or juvenile* or adolesc* or teen* or "young offender*" or "young people" or "young person*" or child* or "early offender" ) OR SU ( youth* or juvenile* or adolesc* or teen* or "young offender*" or "young people" or "young person*" or child* or "early offender" )) AND (AB ( crime* or criminal* or devian* or violen* or delinquen* or offend* or offense* or offence* or recidiv* or reoffen* or breach* or "technical violation*" or arrest* or convict* or charge* or incarcer* or petition* or adjudicat* or caution* or "compliance during supervision" or "return to custody" ) OR TI ( crime* or criminal* or devian* or violen* or delinquen* or offend* or offense* or offence* or recidiv* or reoffen* or breach* or "technical violation*" or arrest* or convict* or charge* or incarcer* or petition* or adjudicat* or caution* or "compliance during supervision" or "return to custody" ) OR SU ( crime* or criminal* or devian* or violen* or delinquen* or offend* or offense* or offence* or recidiv* or reoffen* or breach* or "technical violation*" or arrest* or convict* or charge* or incarcer* or petition* or adjudicat* or caution* or "compliance during supervision" or "return to custody" )) AND (AB (diversion* or divert* or probat* or parole or aftercare or resettlement or reentry or "re-entry" or "after custod*" or supervis* or "graduated sanction*" or "intermediate sanction*" or "early release" or "pretrial release" or "supervised release" or wraparound or reintegrat* or throughcare or "local authority care" or "security training cent*" or "care leaver*" or "detention and training order" or "youth offending team") OR TI (diversion* or divert* or probat* or parole or aftercare or resettlement or reentry or "re-entry" or "after custod*" or supervis* or "graduated sanction*" or "intermediate sanction*" or "early release" or "pretrial release" or "supervised release" or wraparound or reintegrat* or throughcare or "local authority care" or "security training cent*" or "care leaver*" or "detention and training order" or "youth offending team") OR SU (diversion* or divert* or probat* or parole or aftercare or resettlement or reentry or "re-entry" or "after custod*" or supervis* or "graduated sanction*" or "intermediate sanction*" or "early release" or "pretrial release" or "supervised release" or wraparound or reintegrat* or throughcare or "local authority care" or "security training cent*" or "care leaver*" or "detention and training order" or "youth offending team")) AND (AB (evaluat* or effect* or impact* or outcome* or trial* or treat* or program* or randomi* or experiment* or assess* or process or implement* or fidelity or "proof of concept" or "case study" or "focus group" or "pilot study" or "qualitative" or "formative evaluation" or "cost benefit" or "cost effectiveness" or "cost analysis" or "benefit cost" or "theory of change" or "program* theory" or "program model" or "logic model" or "action theory" or "causal map") OR TI (evaluat* or effect* or impact* or outcome* or trial* or treat* or program* or randomi* or experiment* or assess* or process or implement* or fidelity or "proof of concept" or "case study" or "focus group" or "pilot study" or "qualitative" or "formative evaluation" or "cost benefit" or "cost effectiveness" or "cost analysis" or "benefit cost" or "theory of change" or "program* theory" or "program model" or "logic model" or "action theory" or "causal map") OR SU (evaluat* or effect* or impact* or outcome* or trial* or treat* or program* or randomi* or experiment* or assess* or process or implement* or fidelity or "proof of concept" or "case study" or "focus group" or "pilot study" or "qualitative" or "formative evaluation" or "cost benefit" or "cost effectiveness" or "cost analysis" or "benefit cost" or "theory of change" or "program* theory" or "program model" or "logic model" or "action theory" or "causal map")) | | | | |
| **EBSCO Open Dissertations** | **Included databases:**  EBSCO Open Dissertations | **Search fields:**  Title, abstract, subject terms | **Search limiters**:  Date: January 1992 – January 2023 | **Date searched:** January 10, 2023 | **Total hits**: 428 |
|  | **EBSCO Open Dissertations search string/terms used**  AB ( (youth* or juvenile* or adolesc* or teen* or "young offender*" or "young people" or "young person*" or child* or "early offender") and (crime* or criminal* or devian* or violen* or delinquen* or offend* or offense* or offence* or recidiv* or reoffen* or breach* or "technical violation*" or arrest* or convict* or charge* or incarcer* or petition* or adjudicat* or caution* or "compliance during supervision" or "return to custody") and (diversion* or divert* or probat* or parole or aftercare or resettlement or reentry or "re-entry" or "after custod*" or supervis* or "graduated sanction*" or "intermediate sanction*" or "early release" or "pretrial release" or "supervised release" or wraparound or reintegrat* or throughcare or "local authority care" or "security training cent*" or "care leaver*" or "detention and training order" or "youth offending team") and (evaluat* or effect* or impact* or outcome* or trial* or treat* or program* or randomi* or experiment* or assess* or process or implement* or fidelity or "proof of concept" or "case study" or "focus group" or "pilot study" or "qualitative" or "formative evaluation" or "cost benefit" or "cost effectiveness" or "cost analysis" or "benefit cost" or "theory of change" or "program* theory" or "program model" or "logic model" or "action theory" or "causal map") ) OR TI ( (youth* or juvenile* or adolesc* or teen* or "young offender*" or "young people" or "young person*" or child* or "early offender") and (crime* or criminal* or devian* or violen* or delinquen* or offend* or offense* or offence* or recidiv* or reoffen* or breach* or "technical violation*" or arrest* or convict* or charge* or incarcer* or petition* or adjudicat* or caution* or "compliance during supervision" or "return to custody") and (diversion* or divert* or probat* or parole or aftercare or resettlement or reentry or "re-entry" or "after custod*" or supervis* or "graduated sanction*" or "intermediate sanction*" or "early release" or "pretrial release" or "supervised release" or wraparound or reintegrat* or throughcare or "local authority care" or "security training cent*" or "care leaver*" or "detention and training order" or "youth offending team") and (evaluat* or effect* or impact* or outcome* or trial* or treat* or program* or randomi* or experiment* or assess* or process or implement* or fidelity or "proof of concept" or "case study" or "focus group" or "pilot study" or "qualitative" or "formative evaluation" or "cost benefit" or "cost effectiveness" or "cost analysis" or "benefit cost" or "theory of change" or "program* theory" or "program model" or "logic model" or "action theory" or "causal map") ) OR SU ( (youth* or juvenile* or adolesc* or teen* or "young offender*" or "young people" or "young person*" or child* or "early offender") and (crime* or criminal* or devian* or violen* or delinquen* or offend* or offense* or offence* or recidiv* or reoffen* or breach* or "technical violation*" or arrest* or convict* or charge* or incarcer* or petition* or adjudicat* or caution* or "compliance during supervision" or "return to custody") and (diversion* or divert* or probat* or parole or aftercare or resettlement or reentry or "re-entry" or "after custod*" or supervis* or "graduated sanction*" or "intermediate sanction*" or "early release" or "pretrial release" or "supervised release" or wraparound or reintegrat* or throughcare or "local authority care" or "security training cent*" or "care leaver*" or "detention and training order" or "youth offending team") and (evaluat* or effect* or impact* or outcome* or trial* or treat* or program* or randomi* or experiment* or assess* or process or implement* or fidelity or "proof of concept" or "case study" or "focus group" or "pilot study" or "qualitative" or "formative evaluation" or "cost benefit" or "cost effectiveness" or "cost analysis" or "benefit cost" or "theory of change" or "program* theory" or "program model" or "logic model" or "action theory" or "causal map") ) | | | | |
| **Scopus** | **Included databases:**  Scopus | **Search fields:**  Title, abstract, keyword | **Search limiters**:  Date: 1992 – January 2023 | **Date searched:** January 3, 2023 | **Total hits**: 4,952 |
|  | **Scopus search string/terms used**  TITLE-ABS-KEY ( ( youth* OR juvenile* OR adolesc* OR teen* OR "young offender*" OR "young people" OR "young person*" OR child* OR "early offender" ) AND ( crime* OR criminal* OR devian* OR violen* OR delinquen* OR offend* OR offense* OR offence* OR recidiv* OR reoffen* OR breach* OR "technical violation*" OR arrest* OR convict* OR charge* OR incarcer* OR petition* OR adjudicat* OR caution* OR "compliance during supervision" OR "return to custody" ) AND ( diversion* OR divert* OR probat* OR parole OR aftercare OR resettlement OR reentry OR "re-entry" OR "after custod*" OR supervis* OR "graduated sanction*" OR "intermediate sanction*" OR "early release" OR "pretrial release" OR "supervised release" OR wraparound OR reintegrat* OR throughcare OR "local authority care" OR "security training cent*" OR "care leaver*" OR "detention and training order" OR "youth offending team" ) AND ( evaluat* OR effect* OR impact* OR outcome* OR trial* OR treat* OR program* OR randomi* OR experiment* OR assess* OR process OR implement* OR fidelity OR "proof of concept" OR "case study" OR "focus group" OR "pilot study" OR "qualitative" OR "formative evaluation" OR "cost benefit" OR "cost effectiveness" OR "cost analysis" OR "benefit cost" OR "theory of change" OR "program* theory" OR "program model" OR "logic model" OR "action theory" OR "causal map" ) ) AND ( PUBYEAR > 1991) | | | | |
| **Networked Digital Library of Theses and Dissertations (NDLTD)** | **Included databases:**  NDLTD | **Search fields:**  Title, subject | **Search limiters**:  Date: 1992 - 2023 | **Date searched:** January 9, 2023 | **Total hits**: 367 |
|  | **NDLTD search string/terms used:**  (aftercare OR resettlement OR reentry) AND (youth OR child OR juvenile OR adolescent OR offender) | | | | |
| **Open Access Theses and Dissertations** | **Included databases:**  Open Access Theses and Dissertations | **Search fields:**  Title, abstract, subject keyword | **Search limiters**:  Date: 1992-present (Jan 2023)  Language: English | **Date searched:** January 4, 2023 | **Total hits**: 298 |
|  | **Open Access Theses and Dissertations search string/terms used:**  (young OR youth OR child OR adolescent OR juvenile) AND (aftercare OR reentry OR "re-entry" OR resettlement) | | | | |
| **Ovid** | **Included databases:**   - Cochrane Central Register of Controlled Trials - Cochrane Database of Systematic Reviews - Database of Abstracts of Reviews of Effects | **Search fields:**  Advanced keyword (included abstract, title, and subject heading) | **Search limiters**:  Date: ‘last 31 years’ | **Date searched:**  January 2, 2023 | **Total hits**:  859 |
|  | **Ovid search string/terms used:**  ((youth* or juvenile* or adolesc* or teen* or "young offender*" or "young people" or "young person*" or child* or "early offender") and (crime* or criminal* or devian* or violen* or delinquen* or offend* or offense* or offence* or recidiv* or reoffen* or breach* or "technical violation*" or arrest* or convict* or charge* or incarcer* or petition* or adjudicat* or caution* or "compliance during supervision" or "return to custody") and (diversion* or divert* or probat* or parole or aftercare or resettlement or reentry or "re-entry" or "after custod*" or supervis* or "graduated sanction*" or "intermediate sanction*" or "early release" or "pretrial release" or "supervised release" or wraparound or reintegrat* or throughcare or "local authority care" or "security training cent*" or "care leaver*" or "detention and training order" or "youth offending team") and (evaluat* or effect* or impact* or outcome* or trial* or treat* or program* or randomi* or experiment* or assess* or process or implement* or fidelity or "proof of concept" or "case study" or "focus group" or "pilot study" or "qualitative" or "formative evaluation" or "cost benefit" or "cost effectiveness" or "cost analysis" or "benefit cost" or "theory of change" or "program* theory" or "program model" or "logic model" or "action theory" or "causal map")).mp. [mp=ti, ot, tx, kw, ab, fx, sh, hw, ct] | | | | |
| **Proquest** | **Included databases:**   - - Applied Social Sciences Index and Abstracts (ASSIA)   - Canadian Research Index   - National Criminal Justice Reference Service (NCJRS)   - ProQuest Dissertations and Theses   - Social Services Abstracts   - Sociological Abstracts   - Sociology Database | **Search fields:**  Title, abstract, subject | **Search limiters**:  Date: 1992 - 2023 | **Date searched:**  January 2, 2023 | **Total hits**:  6,889 |
|  | **Proquest search string/terms used:**  (abstract(youth* OR juvenile* OR adolesc* OR teen* OR "young offender*" OR "young people" OR "young person*" OR child* OR "early offender") OR title(youth* OR juvenile* OR adolesc* OR teen* OR "young offender*" OR "young people" OR "young person*" OR child* OR "early offender") OR subject(youth* OR juvenile* OR adolesc* OR teen* OR "young offender*" OR "young people" OR "young person*" OR child* OR "early offender")) AND (abstract(crime* or criminal* or devian* or violen* or delinquen* or offend* or offense* or offence* or recidiv* or reoffen* or breach* or "technical violation*" or arrest* or convict* or charge* or incarcer* or petition* or adjudicat* or caution* or "compliance during supervision" or "return to custody") OR title(crime* or criminal* or devian* or violen* or delinquen* or offend* or offense* or offence* or recidiv* or reoffen* or breach* or "technical violation*" or arrest* or convict* or charge* or incarcer* or petition* or adjudicat* or caution* or "compliance during supervision" or "return to custody") OR subject(crime* or criminal* or devian* or violen* or delinquen* or offend* or offense* or offence* or recidiv* or reoffen* or breach* or "technical violation*" or arrest* or convict* or charge* or incarcer* or petition* or adjudicat* or caution* or "compliance during supervision" or "return to custody")) AND (abstract(diversion* or divert* or probat* or parole or aftercare or resettlement or reentry or "re-entry" or "after custod*" or supervis* or "graduated sanction*" or "intermediate sanction*" or "early release" or "pretrial release" or "supervised release" or wraparound or reintegrat* or throughcare or "local authority care" or "security training cent*" or "care leaver*" or "detention and training order" or "youth offending team") OR title(diversion* or divert* or probat* or parole or aftercare or resettlement or reentry or "re-entry" or "after custod*" or supervis* or "graduated sanction*" or "intermediate sanction*" or "early release" or "pretrial release" or "supervised release" or wraparound or reintegrat* or throughcare or "local authority care" or "security training cent*" or "care leaver*" or "detention and training order" or "youth offending team") OR subject(diversion* or divert* or probat* or parole or aftercare or resettlement or reentry or "re-entry" or "after custod*" or supervis* or "graduated sanction*" or "intermediate sanction*" or "early release" or "pretrial release" or "supervised release" or wraparound or reintegrat* or throughcare or "local authority care" or "security training cent*" or "care leaver*" or "detention and training order" or "youth offending team")) AND (abstract(evaluat* or effect* or impact* or outcome* or trial* or treat* or program* or randomi* or experiment* or assess* or process or implement* or fidelity or "proof of concept" or "case study" or "focus group" or "pilot study" or "qualitative" or "formative evaluation" or "cost benefit" or "cost effectiveness" or "cost analysis" or "benefit cost" or "theory of change" or "program* theory" or "program model" or "logic model" or "action theory" or "causal map") OR title(evaluat* or effect* or impact* or outcome* or trial* or treat* or program* or randomi* or experiment* or assess* or process or implement* or fidelity or "proof of concept" or "case study" or "focus group" or "pilot study" or "qualitative" or "formative evaluation" or "cost benefit" or "cost effectiveness" or "cost analysis" or "benefit cost" or "theory of change" or "program* theory" or "program model" or "logic model" or "action theory" or "causal map") OR subject(evaluat* or effect* or impact* or outcome* or trial* or treat* or program* or randomi* or experiment* or assess* or process or implement* or fidelity or "proof of concept" or "case study" or "focus group" or "pilot study" or "qualitative" or "formative evaluation" or "cost benefit" or "cost effectiveness" or "cost analysis" or "benefit cost" or "theory of change" or "program* theory" or "program model" or "logic model" or "action theory" or "causal map")) | | | | |
| **Theses Canada** | **Included databases:**  Theses Canada | **Search fields:**  No specifications possible | **Search limiters:**  n/a | **Date searched:**  December 29, 2022 | **Total hits:**  234 |
|  | **Theses Canada search string/terms used:**  Aftercare AND youth  Resettlement AND youth  Reentry AND youth  Reentry AND juvenile  Aftercare AND juvenile  Resettlement AND juvenile  Aftercare AND offender  Resettlement AND offender  Reentry AND offender | | | | |
| **Web of Science** | **Included databases:**  Web of Science | **Search fields:**  Topic (title, abstract, keyword plus, author keywords) | **Search limiters**:  Date: January 1992 – January 2023 | **Date searched:**  January 3, 2023 | **Total hits**:  3,368 |
|  | **Web of Science search string/terms used:**  (((TS=(youth* OR juvenile* OR adolesc* OR teen* OR "young offender*" OR "young people" OR "young person*" OR child* OR "early offender")) AND TS=(crime* OR criminal* OR devian* OR violen* OR delinquen* OR offend* OR offense* OR offence* OR recidiv* OR reoffen* OR breach* OR "technical violation*" OR arrest* OR convict* OR charge* OR incarcer* OR petition* OR adjudicat* OR caution* OR "compliance during supervision" OR "return to custody")) AND TS=(diversion* OR divert* OR probat* OR parole OR aftercare OR resettlement OR reentry OR "re-entry" OR "after custod*" OR supervis* OR "graduated sanction*" OR "intermediate sanction*" OR "early release" OR "pretrial release" OR "supervised release" OR wraparound OR reintegrat* OR throughcare OR "local authority care" OR "security training cent*" OR "care leaver*" OR "detention and training order" OR "youth offending team")) AND TS=(evaluat* OR effect* OR impact* OR outcome* OR trial* OR treat* OR program* OR randomi* OR experiment* OR assess* OR process OR implement* OR fidelity OR "proof of concept" OR "case study" OR "focus group" OR "pilot study" OR "qualitative" OR "formative evaluation" OR "cost benefit" OR "cost effectiveness" OR "cost analysis" OR "benefit cost" OR "theory of change" OR "program* theory" OR "program model" OR "logic model" OR "action theory" OR "causal map") | | | | |
| **Open access search engines** | | | | | |
| **Directory of Open Access Journals (DOAJ)** | **Included databases:**  DOAJ | **Search fields:**  ‘all fields’ (includes title, abstract, subject, author) | **Search limiters**:  Searched for ‘articles’ | **Date searched:**  January 3, 2023 | **Total hits**:  Full terms returned 0 hits.  Modified terms returned 22 hits |
|  | **DOAJ search string/terms used:**  (youth* OR juvenile* OR adolesc* OR teen* OR "young offender*" OR "young people" OR "young person*" OR child* OR "early offender" ) AND ( crime* OR criminal* OR devian* OR violen* OR delinquen* OR offend* OR offense* OR offence* OR recidiv* OR reoffen* OR breach* OR "technical violation*" OR arrest* OR convict* OR charge* OR incarcer* OR petition* OR adjudicat* OR caution* OR "compliance during supervision" OR "return to custody" ) AND ( diversion* OR divert* OR probat* OR parole OR aftercare OR resettlement OR reentry OR "re-entry" OR "after custod*" OR supervis* OR "graduated sanction*" OR "intermediate sanction*" OR "early release" OR "pretrial release" OR "supervised release" OR wraparound OR reintegrat* OR throughcare OR "local authority care" OR "security training cent*" OR "care leaver*" OR "detention and training order" OR "youth offending team" ) AND ( evaluat* OR effect* OR impact* OR outcome* OR trial* OR treat* OR program* OR randomi* OR experiment* OR assess* OR process OR implement* OR fidelity OR "proof of concept" OR "case study" OR "focus group" OR "pilot study" OR "qualitative" OR "formative evaluation" OR "cost benefit" OR "cost effectiveness" OR "cost analysis" OR "benefit cost" OR "theory of change" OR "program* theory" OR "program model" OR "logic model" OR "action theory" OR "causal map")  **Modified terms:** ‘youth aftercare’; ‘juvenile reentry’; ‘adolescent resettlement’ | | | | |
| **Bielefeld Academic Search Engine (BASE)** | **Included databases:**  BASE | **Search fields:**  Title, subject headings | **Search limiters:**  Text documents | **Date searched:**  January 3, 2023 | **Total hits:**  Full terms returned 0 hits.  Modified terms returned 127 hits |
|  | **BASE search string/terms used:** (youth* OR juvenile* OR adolesc* OR teen* OR "young offender*" OR "young people" OR "young person*" OR child* OR "early offender" ) AND ( crime* OR criminal* OR devian* OR violen* OR delinquen* OR offend* OR offense* OR offence* OR recidiv* OR reoffen* OR breach* OR "technical violation*" OR arrest* OR convict* OR charge* OR incarcer* OR petition* OR adjudicat* OR caution* OR "compliance during supervision" OR "return to custody" ) AND ( diversion* OR divert* OR probat* OR parole OR aftercare OR resettlement OR reentry OR "re-entry" OR "after custod*" OR supervis* OR "graduated sanction*" OR "intermediate sanction*" OR "early release" OR "pretrial release" OR "supervised release" OR wraparound OR reintegrat* OR throughcare OR "local authority care" OR "security training cent*" OR "care leaver*" OR "detention and training order" OR "youth offending team" ) AND ( evaluat* OR effect* OR impact* OR outcome* OR trial* OR treat* OR program* OR randomi* OR experiment* OR assess* OR process OR implement* OR fidelity OR "proof of concept" OR "case study" OR "focus group" OR "pilot study" OR "qualitative" OR "formative evaluation" OR "cost benefit" OR "cost effectiveness" OR "cost analysis" OR "benefit cost" OR "theory of change" OR "program* theory" OR "program model" OR "logic model" OR "action theory" OR "causal map")  **Modified terms:** ‘youth aftercare’; ‘juvenile reentry’; ‘adolescent resettlement’ | | | | |
| **CrimRxiv** | **Included databases:**  CrimRxiv | **Search fields:**  No specifications possible | **Search limiters:**  n/a | **Date searched:**  January 3, 2023 | **Total hits:**  Full terms returned 0 hits.  Modified terms returned 10 hits |
|  | **CrimRxiv search string/terms used:** (youth* OR juvenile* OR adolesc* OR teen* OR "young offender*" OR "young people" OR "young person*" OR child* OR "early offender" ) AND ( crime* OR criminal* OR devian* OR violen* OR delinquen* OR offend* OR offense* OR offence* OR recidiv* OR reoffen* OR breach* OR "technical violation*" OR arrest* OR convict* OR charge* OR incarcer* OR petition* OR adjudicat* OR caution* OR "compliance during supervision" OR "return to custody" ) AND ( diversion* OR divert* OR probat* OR parole OR aftercare OR resettlement OR reentry OR "re-entry" OR "after custod*" OR supervis* OR "graduated sanction*" OR "intermediate sanction*" OR "early release" OR "pretrial release" OR "supervised release" OR wraparound OR reintegrat* OR throughcare OR "local authority care" OR "security training cent*" OR "care leaver*" OR "detention and training order" OR "youth offending team" ) AND ( evaluat* OR effect* OR impact* OR outcome* OR trial* OR treat* OR program* OR randomi* OR experiment* OR assess* OR process OR implement* OR fidelity OR "proof of concept" OR "case study" OR "focus group" OR "pilot study" OR "qualitative" OR "formative evaluation" OR "cost benefit" OR "cost effectiveness" OR "cost analysis" OR "benefit cost" OR "theory of change" OR "program* theory" OR "program model" OR "logic model" OR "action theory" OR "causal map")  **Modified terms:** ‘youth aftercare’; ‘juvenile reentry’; ‘adolescent resettlement’ | | | | |

**Table A2. Grey Literature Search**

| **Website** | **Search terms used** | **Search fields** | **Search limiters** | **Date searched** | **Total hits** |
| --- | --- | --- | --- | --- | --- |
| Ministry of Justice UK | Modified terms: ‘aftercare’; ‘re-entry’; ‘resettlement’ | Keyword search bar | Filtered by ‘crime, justice, and law’  Content types: “research and statistics”, “services”, and “policy papers and consultations” | January 5, 2023 | 39 |
| Home Office UK | Modified terms: ‘Youth aftercare program’; ‘Juvenile offender reentry’;  ‘Adolescent resettlement’ | Keyword search bar | Filtered by ‘crime, justice, and law’  Content types: “research and statistics”, “services”, and “policy papers and consultations” | January 3, 2023 | 1,308 |
| National Institute of Justice | Modified terms: ‘aftercare’; ‘re-entry’; ‘resettlement’ | Keyword search bar | Searched under ‘publications’ | January 5, 2023 | 24 |
| New South Wales Bureau of Crime Statistics and Research | Modified terms: ‘aftercare’; ‘re-entry’; ‘resettlement’ | Keyword search bar  Browsed topic pages | Topic pages: Children, juveniles, and young people  Recidivism/Re-offending | January 5, 2023 | 130 |
| Office of Juvenile Justice and Delinquency Prevention | Modified terms: ‘aftercare’; ‘re-entry’; ‘resettlement’ | Keyword search bar | Searched under ‘publications’ | January 5, 2023 | 20 |
| US Department of Justice | Modified terms: ‘aftercare’ | Keyword search bar | Searched under ‘publications’ | January 5, 2023 | 318 |
| Youth Justice Board | None (hand-searched publications page) | Browsed ‘research and statistics’ page | Filtered documents published by Youth Justice Board | January 5, 2023 | 21 |
| Philadelphia Opportunities Industrialization Center, Youth Advocacy Plus Program (OIC-YAPP) | None (hand-searched website)  Modified terms: ‘youth advocacy plus program’; youth advocacy plus program AND Philadelphia’ | Google search | n/a | January 10, 2023 | No hits on website  Google: first 100 hits per terms |
| Abraxas Non-Residential Care (Abraxas NRC) | Modified terms: ‘Preparation for adolescent transition to home’ | Keyword search bar; hand-searched webpage | n/a | January 10, 2023 | 3 |
| Children's Aftercare Reentry Experience (CARE) | Modified terms: ‘Children’s Aftercare Reentry Experience’ | No website available; Google searched program | n/a | January 10, 2023 | 9 |
| Skillman Aftercare Experiment | Modified terms:  ‘Skillman Aftercare Experiment’ | No website available; Google searched program | n/a | January 10, 2023 | 100 |
| Intensive Parole Program (IPP) | Modified terms:  ‘Virginia Intensive Parole Program’ | No website available; Google searched program | n/a | January 10, 2023 | 100 |
| Australian Institute of Criminology | Modified terms:  ‘Youth aftercare program’; ‘Juvenile offender reentry’;  ‘Adolescent resettlement’ | Keyword search bar | Filtered by ‘publications’ | January 3, 2023 | 0 |
| Confederation of European Probation | Modified terms:  ‘Youth aftercare program’; ‘Juvenile offender reentry’;  ‘Adolescent resettlement’ | Keyword search bar  Hand-searched ‘knowledge base’ and ‘juvenile offender’ pages | n/a | January 3, 2023 | 1 |
| Government of Canada   - - Department of Justice   - Public Safety Canada | Modified terms:  ‘Youth aftercare program’; ‘Juvenile offender reentry’;  ‘Adolescent resettlement’ | Keyword search bar | n/a | January 3, 2023 | 486 |
| European Crime Prevention Network | Modified terms:  ‘Youth aftercare program’; ‘Juvenile offender reentry’;  ‘Adolescent resettlement’ | Keyword search bar  Hand-searched ‘knowledge center’ and ‘research’ pages | Knowledge center filtered by ‘youth crime’ | January 3, 2023 | 170 |
| Boys and Girls Clubs of America | None (hand-searched website)  Modified terms (Google): ‘Boys and Girls Clubs of America Targeted Re-entry’ | Google search | n/a | January 3, 2023 | 375 |
| Clay County Reentry Services Project | Modified terms:  ‘Clay County Reentry Services Project’ | Google search | n/a | January 3, 2023 | 240 |
| Westchester County Aftercare Program | Modified terms: ‘Westchester County Aftercare Program’ | Google search | n/a | January 3, 2023 | 294 |
| The Serious and Violent Offender Reentry Initiative (SVORI) | Modified terms:  ‘Serious and Violent Offender Reentry Initiative’ | Google search | n/a | January 3, 2023 | 341 |
| **Meeting archives** | **Search terms used** | **Search fields** | **Search limiters** | **Date searched** | **Total hits** |
| American Society of Criminology | None | Hand-searched conference programs | Programs available 1999-2022 | January 3, 2023 | n/a |
| British Society of Criminology | None | Hand-searched conference programs | Programs available 2015-2022 | January 3, 2023 | n/a |
| European Society of Criminology | None | Hand-searched conference programs | Programs available 2001-2021 | January 3, 2023 | n/a |
| **Academic journals** | **Search terms used** | **Search fields** | **Search limiters** | **Date searched** | **Total hits** |
| British Journal of Criminology | Full search terms | Hand-searched tables of contents | Date: January 2021 – January 2023 | January 5, 2023 | 463 |
| Canadian Journal of Criminology and Criminal Justice | Full search terms | Hand-searched tables of contents | Date: January 2021 – January 2023 | January 5, 2023 | 43 |
| Corrections: Policy, Practice, & Research | Full search terms | Hand-searched tables of contents | Date: January 2021 – January 2023 | January 5, 2023 | 49 |
| Crime & Delinquency | Full search terms | Hand-searched tables of contents | Date: January 2021 – January 2023 | January 5, 2023 | 399 |
| Crime Prevention & Community Safety | Full search terms | Hand-searched tables of contents | Date: January 2021 – January 2023 | January 5, 2023 | 58 |
| Criminal Justice and Behavior | Full search terms | Hand-searched tables of contents | Date: January 2021 – January 2023 | January 5, 2023 | 228 |
| Criminal Justice Review | Full search terms | Hand-searched tables of contents | Date: January 2021 – January 2023 | January 5, 2023 | 157 |
| Criminology & Public Policy | Full search terms | Hand-searched tables of contents | Date: January 2021 – January 2023 | January 5, 2023 | 77 |
| Criminology and Criminal Justice | Full search terms | Hand-searched tables of contents | Date: January 2021 – January 2023 | January 5, 2023 | 199 |
| European Journal of Crime, Criminal Law, and Criminal Justice | Full search terms | Hand-searched tables of contents | Date: January 2021 – January 2023 | January 5, 2023 | 42 |
| European Journal of Criminology | Full search terms | Hand-searched tables of contents | Date: January 2021 – January 2023 | January 5, 2023 | 223 |
| European Journal on Criminal Policy and Research | Full search terms | Hand-searched tables of contents | Date: January 2021 – January 2023 | January 5, 2023 | 64 |
| Federal Probation | Full search terms | Hand-searched tables of contents | Date: January 2021 – January 2023 | January 5, 2023 | 40 |
| International Journal of Offender Therapy and Comparative Criminology | Full search terms | Hand-searched tables of contents | Date: January 2021 – January 2023 | January 5, 2023 | 339 |
| Journal of Community Corrections | Full search terms | Hand-searched tables of contents | Date: January 2021 – January 2023 | January 5, 2023 | 44 |
| Journal of Experimental Criminology | Full search terms | Hand-searched tables of contents | Date: January 2021 – January 2023 | January 5, 2023 | 74 |
| Journal of Offender Rehabilitation | Full search terms | Hand-searched tables of contents | Date: January 2021 – January 2023 | January 5, 2023 | 53 |
| Journal of Research Crime and Delinquency | Full search terms | Hand-searched tables of contents | Date: January 2021 – January 2023 | January 5, 2023 | 72 |
| Juvenile and Family Court Journal | Full search terms | Hand-searched tables of contents | Date: January 2021 – January 2023 | January 5, 2023 | 40 |
| Probation Journal | Full search terms | Hand-searched tables of contents | Date: January 2021 – January 2023 | January 5, 2023 | 102 |
| Residential Treatment for Children & Youth | Full search terms | Hand-searched tables of contents | Date: January 2021 – January 2023 | January 5, 2023 | 54 |
| Youth Justice | Full search terms | Hand-searched tables of contents | Date: January 2021 – January 2023 | January 5, 2023 | 61 |
| Youth Violence and Juvenile Justice | Full search terms | Hand-searched tables of contents | Date: January 2021 – January 2023 | January 5, 2023 | 51 |

**Appendix 4. Characteristics of studies awaiting classification**

| **Study ID** | **Reason for exclusion** |
| --- | --- |
| Beckworth-Belford (2015) | Requested from InterLibrary Loan service, could not retrieve full text |
| Bergseth (2009) | Conference presentation; published papers corresponding to study were retrieved |
| Bowers & Flynn (2002) | Conference presentation; published papers corresponding to study were retrieved |
| Cantora & White (2008) | Conference presentation; no corresponding documents available |
| Clemons (2013) | Requested from InterLibrary Loan service, could not retrieve full text |
| Correira (2014) | Requested from InterLibrary Loan service, could not retrieve full text |
| Dawes & Winterfield (2008) | Conference presentation; no corresponding documents available |
| Fischer (2013) | Requested from InterLibrary Loan service, could not retrieve full text |
| Gretak et al. (2017) | Requested from InterLibrary Loan service, could not retrieve full text |
| Hazel (2010) | Conference presentation; published papers corresponding to study were retrieved |
| Hine (2017) | Conference presentation; no corresponding documents available |
| Hutchins (2015) | Requested from InterLibrary Loan service, could not retrieve full text |
| Jainchill et al. (2007) | Conference presentation; published papers corresponding to study were retrieved |
| Jarjoura (2002) | Conference presentation; published papers corresponding to study were retrieved |
| Jarjoura (2004) | Requested from InterLibrary Loan service, could not retrieve full text |
| Jarjoura (2007) | Requested from InterLibrary Loan service, could not retrieve full text |
| Jarjoura et al. (2006) | Conference presentation; published papers corresponding to study were retrieved |
| Jarjoura et al. (2007) | Conference presentation; published papers corresponding to study were retrieved |
| Jarjoura et al. (2008) | Conference presentation; published papers corresponding to study were retrieved |
| Kaczynski & Lersch (2001) | Conference presentation; no corresponding documents available |
| Lattimore & Steffey (2008) | Conference presentation; no corresponding documents available |
| Liberman & Hussemann (2017) | Conference presentation; published papers corresponding to study were retrieved |
| Liddle (2007) | Conference presentation; published papers corresponding to study were retrieved |
| Long (2013) | Requested from InterLibrary Loan service, could not retrieve full text |
| Machkovitz (1993) | Requested from InterLibrary Loan service, could not retrieve full text |
| Malone & Harris (2013) | Conference presentation; published papers corresponding to study were retrieved |
| McCrary (2003) | Requested from InterLibrary Loan service, could not retrieve full text |
| McDonald & Jonson (2013) | Conference presentation; no corresponding documents available |
| McNulty et al. (2000) | Conference presentation; published papers corresponding to study were retrieved |
| Meisel (1999) | Conference presentation; published papers corresponding to study were retrieved |
| Meisel (2000) | Conference presentation; published papers corresponding to study were retrieved |
| Meisel (2001) | Conference presentation; published papers corresponding to study were retrieved |
| Meisel (2003) | Conference presentation; published papers corresponding to study were retrieved |
| Meisel (2008) | Conference presentation; no corresponding documents available |
| Mendenhall et al. (1999) | Conference presentation; no corresponding documents available |
| Mendenhall & Armstrong (2002) | Conference presentation; published papers corresponding to study were retrieved |
| Moore & Hobbs (2017) | Conference presentation; published papers corresponding to study were retrieved |
| NACRO (1996) | Requested from InterLibrary Loan service, could not retrieve full text |
| Ndrecka & Harbinson (2011) | Conference presentation; no corresponding documents located |
| Parker (2013) | Requested from InterLibrary Loan service, could not retrieve full text |
| Pinanzu (2017) | Requested from InterLibrary Loan service, could not retrieve full text |
| Ramos (2022) | Requested from InterLibrary Loan service, could not retrieve full text |
| Seymour (2003 | Requested from InterLibrary Loan service, could not retrieve full text |
| Slavin (2022) | Requested from InterLibrary Loan service, could not retrieve full text |
| Timpanaro (2020) | Requested from InterLibrary Loan service, could not retrieve full text |
| Wagner et al. (2001) | Conference presentation; published papers corresponding to study were retrieved |
| Watkins et al. (2019) | Conference presentation; no corresponding documents available |
| Weissman & Wolf (2005) | Conference presentation; no corresponding documents available |
| West & Comer-Cook (2005) | Requested from InterLibrary Loan service, could not retrieve full text |
| Will et al. (2002) | Conference presentation; no corresponding documents available |
| Young et al. (2003) | Requested from InterLibrary Loan service, could not retrieve full text |
